# Computerized cognitive training in attention-deficit/hyperactivity disorder (ADHD): a meta-analysis of randomized controlled trials with blinded and objective outcomes

**DOI:** 10.1038/s41380-023-02000-7

**Published:** 2023-03-29

**Authors:** Samuel J. Westwood, Valeria Parlatini, Katya Rubia, Samuele Cortese, Edmund J. S. Sonuga-Barke, European ADHD Guidelines Group (EAGG), T. Banaschewski, D. Baeyens, S. Bölte, D. Brandeis, J. Buitelaar, S. Carucci, D. Coghill, D. Daley, M. Döpfner, M. Ferrin, C. Galera, C. Hollis, M. Holtmann, D. Purper-Ouakil, P. Nagy, P. Santosh, E. Simonoff, E. J. Sonuga-Barke, C. A. Soutullo, A. Stringaris, A. Thapar, S. van der Oord, B. J. van den Hoofdakker, A. Zuddas

**Affiliations:** 1 Department of Psychology, Institute of Psychiatry, Psychology, Neuroscience, King’s College London, London, UK; 2 School of Social Science, University of Westminster, London, UK; 3 Department of Child and Adolescent Psychiatry, Institute of Psychiatry, Psychology and Neuroscience, King’s College London, London, UK; 4 Centre for Innovation in Mental Health, School of Psychology, Faculty of Environmental and Life Sciences, University of Southampton, Southampton, UK; 5 Clinical and Experimental Sciences (CNS and Psychiatry), Faculty of Medicine, University of Southampton, Southampton, UK; 6 Solent NHS Trust, Southampton, UK; 7 Hassenfeld Children’s Hospital at NYU Langone, New York University Child Study Center, New York City, New York USA; 8 Division of Psychiatry and Applied Psychology, School of Medicine, University of Nottingham, Nottingham, UK; 9 Child and Adolescent Psychiatry and Psychotherapy, Central Institute of Mental Health, Medical Faculty Mannheim, University of Heidelberg, Mannheim, Germany; 10 Research Unit Parenting and Special Education, Faculty of Psychology and Educational Sciences, Katholieke Universiteit Leuven, Leuven, Belgium; 11 Center of Neurodevelopmental Disorders (KIND), Centre for Psychiatry Research, Stockholm, Sweden; 12 Department of Women’s and Children’s Health, Karolinska Institutet & Stockholm Health Care Services, Region Stockholm, Stockholm, Sweden; 13 Child and Adolescent Psychiatry, Stockholm Health Care Services, Region Stockholm, Stockholm, Sweden; 14 Curtin Autism Research Group, Curtin School of Allied Health, Curtin University, Perth, WA Australia; 15 Department of Child and Adolescent Psychiatry and Psychotherapy, University Hospital of Psychiatry, Zurich, Switzerland; 16 Neuroscience Center Zurich, University of Zurich, Zurich, Switzerland; 17 Center for Integrative Human Physiology, University of Zurich, Zurich, Switzerland; 18 ETH Zurich, Zurich, Switzerland; 19 Department of Cognitive Neuroscience, Donders Institute for Brain, Cognition and Behaviour, Radboudumc, Nijmegen, Netherlands; 20 Karakter Child and Adolescent Psychiatry, Nijmegen, Netherlands; 21 Department of Biomedical Sciences, Section of Neuroscience & Clinical Pharmacology, University of Cagliari, Cagliari, Italy; 22 Child & Adolescent Neuropsychiatry Unit, “A.Cao” Paediatric Hospital, “G.Brotzu”, Hospital Trust, Cagliari, Italy; 23 Departments of Paediatrics and Psychiatry, University of Melbourne, Melbourne, VIC Australia; 24 NTU Psychology, School of Social Science, Nottingham Trent University, Nottingham, UK; 25 Department of Child and Adolescent Psychiatry, Psychosomatics and Psychotherapy, Faculty of Medicine and University Hospital Cologne, University of Cologne, Cologne, Germany; 26 School of Child and Adolescent Cognitive Behavior Therapy (AKiP), Faculty of Medicine and University Hospital Cologne, University of Cologne, Cologne, Germany; 27 Department of Psychology, Faculty of Human Sciences, University of Cologne, Cologne, Germany; 28 Barnet Enfield and Haringey NHS Trust and Recognition Health London, London, UK; 29 University of Bordeaux, Bordeaux, France; 30 INSERM, Bordeaux Population Health Center, UMR1219 Bordeaux, France; 31 Centre Hospitalier Perrens, Bordeaux, France; 32 Research Unit on Children’s Psychosocial Maladjustment, Montreal, QC Canada; 33 Division of Psychiatry and Applied Psychology, School of Medicine and National Institute for Health Research MindTech Mental Health MedTech Cooperative and Centre for ADHD and Neurodevelopmental Disorders Across the Lifespan, Institute of Mental Health, University of Nottingham, Nottingham, UK; 34 LWL-University Hospital for Child and Adolescent Psychiatry, Ruhr-University Bochum, Hamm, Germany; 35 CHU Montpellier, St Eloi University Hospital, Child and Adolescent Psychiatry (MPEA1) 80, avenue Augustin Fliche, 34295 Montpellier, France; 36 CESP INSERM U1018, UVSQ Psychiatry, Development and Trajectories, Villejuif, France; 37 Division of Neurodevelopmental Disorders, Bethesda Children’s Hospital, Budapest, Hungary; 38 South London and Maudsley NHS Foundation Trust, Maudsley Hospital, Denmark Hill, London, UK; 39 Department of Child & Adolescent Psychiatry, Aarhus University, Aarhus, Denmark; 40 ADHD Outpatient Program, Louis A. Faillace MD Department of Psychiatry and Behavioral Sciences, The University of Texas Health Science Center, McGovern Medical School, Houston, TX USA; 41 Division of Psychiatry and Department of Clinical, Educational & Health Psychology, University College London, 1-19 Torrington Place, London, WC1E 6HB UK; 42 Department of Psychological Medicine and Clinical Neurosciences, MRC Centre for Neuropsychiatric Genetics and Genomics, Cardiff University, Cardiff, UK; 43 Clinical Psychology, KU Leuven, Leuven, Belgium; 44 Developmental Psychology, University of Amsterdam, Amsterdam, Netherlands; 45 Department of Child and Adolescent Psychiatry, University of Groningen, University Medical Center, Groningen, The Netherlands; 46 Accare Child Study Center, Groningen, The Netherlands; 47 Department of Clinical Psychology and Experimental Psychopathology, University of Groningen, Groningen, The Netherlands; 48 Child & Adolescent Neuropsychiatry, Cagliari, Italy

**Keywords:** ADHD, Neuroscience

## Abstract

This meta-analysis investigated the effects of computerized cognitive training (CCT) on clinical, neuropsychological and academic outcomes in individuals with attention-deficit/hyperactivity disorder (ADHD). The authors searched PubMed, Ovid, and Web of Science until 19th January 2022 for parallel-arm randomized controlled trials (RCTs) using CCT in individuals with ADHD. Random-effects meta-analyses pooled standardized mean differences (SMD) between CCT and comparator arms. RCT quality was assessed with the Cochrane Risk of Bias 2.0 tool (PROSPERO: CRD42021229279). Thirty-six RCTs were meta-analysed, 17 of which evaluated working memory training (WMT). Analysis of outcomes measured immediately post-treatment and judged to be “probably blinded” (PBLIND; trial *n* = 14) showed no effect on ADHD total (SMD = 0.12, 95%CI[−0.01 to −0.25]) or hyperactivity/impulsivity symptoms (SMD = 0.12, 95%[−0.03 to−0.28]). These findings remained when analyses were restricted to trials (*n*: 5–13) with children/adolescents, low medication exposure, semi-active controls, or WMT or multiple process training. There was a small improvement in inattention symptoms (SMD = 0.17, 95%CI[0.02–0.31]), which remained when trials were restricted to semi-active controls (SMD = 0.20, 95%CI[0.04–0.37]), and doubled in size when assessed in the intervention delivery setting (*n* = 5, SMD = 0.40, 95%CI[0.09–0.71]), suggesting a setting-specific effect. CCT improved WM (verbal: *n* = 15, SMD = 0.38, 95%CI[0.24–0.53]; visual-spatial: *n* = 9, SMD = 0.49, 95%CI[0.31–0.67]), but not other neuropsychological (e.g., attention, inhibition) or academic outcomes (e.g., reading, arithmetic; analysed *n*: 5–15). Longer-term improvement (at ~6-months) in verbal WM, reading comprehension, and ratings of executive functions were observed but relevant trials were limited in number (*n*: 5–7). There was no evidence that multi-process training was superior to working memory training. In sum, CCT led to shorter-term improvements in WM, with some evidence that verbal WM effects persisted in the longer-term. Clinical effects were limited to small, setting specific, short-term effects on inattention symptoms.

## Introduction

Attention-deficit/hyperactivity disorder (ADHD) is a neurodevelopmental condition characterized by developmentally-inappropriate, persistent, pervasive and impairing inattention and/or hyperactivity/impulsivity symptoms [1]. ADHD medications, particularly psychostimulants, provide both clinically significant symptomatic relief and reduction of impairment, at least in the short term [2–6], and are recommended as part of multi-modal treatment strategies alongside psycho-social therapies (e.g., parent training) and/or psychoeducation programs [7].

Computerized cognitive training (CCT) has also been investigated as a treatment option for those with ADHD. This is motivated by the notion that ADHD is potentially the result of weaknesses in neuropsychological processes thought to mediate causal pathways between originating causes (i.e., genes and environment) and symptom expression [6]. Indeed, at the group level, individuals with ADHD typically perform worse than neurotypical individuals on computerized neuropsychological measures of a wide-range of cognitive processes, especially motor and interference inhibition, sustained attention and vigilance, switching, working memory (WM), and time perception [6, 8]. Neuroimaging evidence suggests these abnormalities are underpinned by structural and functional alterations across a wide range of cortical and subcortical brain circuits [6, 9, 10]. However, the search for a neuropsychologically-based intervention for ADHD is complicated by several factors. First, neuropsychological heterogeneity, because different individuals may be affected by deficits in different cognitive processes and brain networks [6]. Second, the lack of correlation between treatment related changes in ADHD symptoms and improvements in neuropsychological functioning [2, 11]. Third, the overlap of neurophysiological profiles between ADHD and other conditions (e.g., learning disabilities, conduct disorder, and oppositional defiant disorder) [2].

CCT programs are designed to target relevant brain systems, strengthen cognitive skills and processes, and therefore reduce ADHD symptoms and associated impairment. To do this, they aim to exploit the brain’s inherent neuroplasticity [8, 12, 13]. They usually target one or more cognitive processes (e.g., motor inhibition, interference inhibition, sustained attention, and/or WM) via an adaptive protocol (i.e., task difficultly increases as performance improves) to enhance and promote longer-term neuroplastic changes [8]. On behalf of the European ADHD Guidelines Group (EAGG), we assessed the efficacy of CCT for ADHD in two meta-analyses of randomised controlled trials - one published in 2013 (Sonuga-Barke et al. 2013) [14] and an update in 2015 (Cortese et al. 2015) [15]. To address the paucity of well-blinded outcomes, both meta-analyses estimated effect sizes across the range of degrees of blinding – i.e., comparing effects from outcomes we judged to be *most proximal* (MPROX) to the intervention setting (and therefore the likely least blinded, e.g., parent rating in a home-delivered intervention) and outcomes we judged to be *probably blinded* (PBLIND; i.e., the most blinded outcome assessor). Interestingly, in Cortese et al. (2015) [15], statistically significant moderate improvements in MPROX measures of ADHD total and inattentive symptoms dropped substantially to marginal or non-significant levels with PBLIND measures. This is consistent with the notion that MPROX ratings are potentially subject to outcome assessor bias. Further, interventions targeting multiple cognitive processes showed encouraging effects, but analyses were mainly based on non-PBLIND outcomes. Finally, laboratory measures of visual or verbal WM showed significant small-to-moderate improvements. Other meta-analyses reported similarly small improvements in ADHD symptoms [16, 17] and neuropsychological functions [17–19], but these included randomized and non-randomized trials [17], individuals with and without an ADHD diagnosis or who met cut-off on validated questionnaire measures [16], only four trials [16, 18], or collapsed computerized with non-computerized or multi-modal cognitive training [19], all of which limit the interpretability of their findings.

Because of the relatively small number of high-quality trials with PBLIND outcomes at that time, our prior meta-analyses were unable to provide a solid estimate of the efficacy of CCT, an analysis of whether certain intervention types were better than others, or whether they improved different neuropsychological and academic outcomes. However, since Cortese et al. [15], which included 16 RCTs, there have been a considerable number of new RCTs published, many with larger samples, well-controlled designs and blinded outcomes. Therefore, we report an updated systematic review and meta-analysis that allowed us to focus on ADHD symptom improvement using PBLIND measures as our primary outcome. We also address several outstanding questions about different neuropsychological and academic outcomes with a greater degree of granularity of analysis. We were especially interested to further test, using PBLIND outcomes, the provisional finding derived from non-PBLIND analyses that multi-process training (MPT) is superior to single process training [15]. We also addressed the issue of the setting of measurement. This is because in our prior analysis PBLIND outcomes were mainly measured in a setting *different* from the intervention setting (e.g., teacher ratings of school-related behaviours with a home-based intervention), which confounds the blinded status of ratings with the setting of intervention delivery, meaning that PBLIND outcomes could be indexing the issue of generalisation, not just outcome assessor bias. Our update addressed this issue through a sensitivity analysis that included only trials with PBLIND outcomes that were measured in the intervention setting. Finally, and importantly, to extend the analyses by Cortese et al. [15], we aimed to include participants of all ages, to capture the growing interest in studies in pre-school and adult samples with ADHD.

## Materials & methods

This study followed a preregistered protocol (PROSPERO ID: CRD42021229279; for deviations, see Supplement), and was reported in line with PRISMA 2020 [20] (see Supplementary Table 1) and PRISMA-S [21] (see Supplementary Table 2).

### Eligibility criteria

We included parallel-arm RCTs with participants of any age that had a clinical ADHD, or equivalent hyperkinetic syndrome, diagnosis as defined by DSM-III/ICD-9 onwards (any subtype/presentation) or were above cut-off on validated ADHD rating scales, regardless of the presence of common-comorbidities. CCTs must have been implemented using fully computer-based procedures with an adaptive component – i.e., training difficulty increased as performance improved. Eligible comparator arms were semi-active (non-adaptive CCT), non-active (treatment as usual [TAU], wait list control [WLC]), or placebo pill. Where trials had two comparator conditions (e.g., WLC and training control as well as the active treatment) the condition representing the most rigorous control was selected (e.g., attention control over a WLC). All RCTs must have been peer-reviewed and published in an academic journal, and reported a validated outcome measure of ADHD symptoms, neuropsychological processes, and/or academic outcomes. We excluded RCTs with participants with ADHD with a rare comorbidity (e.g., Fragile X syndrome) that was used as a trial inclusion criterion (i.e., all participants had that comorbidity), or when CCT was only delivered in combination with or adjunct to another distinctive planned active treatment that was administered as part of the trial (e.g., parent training, neurofeedback, or ADHD medication plus cognitive training).

### Information sources

We searched PubMed (MEDLINE), OVID (PsycInfo, Medline, Embase+Embase Classic), and Web of Science (science citation index expanded, Biological abstracts, Biosis, Food science and technology abstracts) until 19th January 2022 using variations of terms for ADHD, RCTs, and cognitive training (see Supplement). Database searches were supplemented by hand searching of published relevant systematic reviews or meta-analyses or of references in individual papers.

### Selection, data collection, risk of bias procedures

Two authors (SJW, VP) independently i) screened all article titles and abstracts; ii) read the full text of articles that passed title/abstract screening to determine final inclusion; iii) extracted relevant data (see Data Items section below) and iv) assessed all eligible reports (i.e., all peer-reviewed publications from eligible RCTs) with the Risk of Bias (RoB) 2.0 tool [22]. The RoB tool was used to evaluate each RCT across 5 domains (i.e., randomisation process; deviations from intended interventions; missing outcome data; measurement of the outcome; selection of the reported result), with signalling questions used to evaluate each domain as having either “low risk”, “some concerns”, or “high risk” of bias relating to a rater’s confidence in the reported results. The overall RoB for each RCT was derived from the highest (i.e., most severe) RoB level in any of the five domains. In some cases, the same outcome from an RCT was reported in several papers that had different sample sizes. In these cases, we selected data from the outcome based on the largest sample size only. Authors were systematically contacted for unpublished information and data (at least two e-mail contacts separated by at least two to three weeks). ESB and SC resolved disagreements.

### Data extraction

Means and standard deviations at all available time points were extracted from validated rating scales/subscales that directly measured ADHD total symptoms or sub-dimensions (hyperactivity/impulsivity, inattention) or ADHD-relevant neuropsychological outcomes, and academic outcomes. Consistent with previous EAGG protocols, if multiple ADHD outcomes were reported for each assessor, we selected the one for analysis based on the following hierarchy; i) ADHD Rating Scale [23], ii) Swanson, Nolan, and Pelham Rating Scale (SNAP) ADHD (any version) [24, 25], iii) Conners’ rating scale (any version; ADHD-Index or DSM-subscales) (for list of versions, see 28), iv) or other ADHD scales. If such diagnostic criteria-based measures were unavailable, alternatives including ratings of ADHD behaviours were selected with the following order; i) Conners’ non-DSM subscales; ii) Rutter Scale (hyperactivity subscale) [26] or Strengths and Difficulties Questionnaire (hyperactivity/inattention items) [27]; and iii) Child Behaviour Checklist (CBCL; attention problems) [28]. In an extension of EAGG protocols, in trials with adult participants, self-ratings were allowed in the place of parent or teacher ratings (when applicable). Self-ratings were not selected in trials with children/adolescents as this age group were judged to be relatively less reliable and may underestimate ADHD severity [29].

For ADHD-related outcomes we distinguished MPROX from PBLIND ratings. MPROX were ratings from individuals judged to be most proximal to the intervention, which were typically least blinded to intervention allocation (e.g., parents if home-based; teachers if school-based; investigators or clinicians if lab or clinic-based; or self-ratings by adults regardless of intervention setting). PBLIND outcomes were the most blinded outcome where assessors were judged to be probably unaware of treatment allocation. We judged an outcome assessor to be PBLIND if they were a) distal from the intervention setting, b) independent coders rating in the intervention setting, or c) could be blinded by design even if proximal to the treatment setting (e.g., blinded parent if home-based with a non-adaptive CCT control arm). Where multiple PBLIND outcomes were available, we selected the one more distal to the treatment setting (e.g., teacher if home-based; parent if school-based) or an independent coder. Outcome assessors were judged to not be PBLIND if the trial design prevented concealment of group allocation (e.g., trials with a WLC, TAU, or treatment control) or there was evidence that blinding broke during the trial (e.g., authors confirmed blinding integrity failed either in the manuscript or via personal communication). MPROX was by definition available for all trials that met the inclusion criteria, while PBLIND was only available for some trials. In some trials, MPROX and PBLIND outcomes were based on the same assessment (i.e., where there was only one outcome measure, and the assessor was probably blinded).

### Statistical analysis

For ADHD symptoms, we analysed MPROX and PBLIND outcomes, but our primary analyses were PBLIND outcomes because they provide a more robust and bias-free estimate of CCT effects. For other outcomes (i.e., ratings of executive function and academic outcomes), we report MPROX outcomes if sufficient (i.e., 5 trials or more) PBLIND trials were not available. Our primary outcome was PBLIND outcomes of ADHD symptoms (total combined) measured at the first time point after the final CCT session (i.e., post-assessment). We also report PBLIND assessments of inattention and hyperactivity/Impulsivity symptoms separately. Other outcomes were neuropsychological and academic outcomes at post-assessment or longer-term follow-up (≥3-months after the final CCT session). Given the variety of neuropsychological/cognitive outcome measures, wherever possible, we took the pragmatic decision to group outcomes together that we judged to tap the same or similar core constructs, with judgments informed by factor analyses and meta-analytic evidence where possible [6, 19, 30]. Where multiple measures were available for a single outcome (as was sometimes the case for laboratory measures), the measure most frequently reported across all included trials was selected for analysis. If multiple longer-term follow-up assessments were reported, we selected the outcome measured at the timepoint most frequently assessed across the included trials.

Effect size estimates were based on standardized mean differences (SMDs), which were calculated as mean baseline- to post-assessment (or follow-up) change in the intervention group minus the mean baseline- to post-assessment (or follow-up) change in the control group divided by the pooled baseline standard deviation with Hedges’ *g* small sample bias adjustment [31, 32]. We conducted random effects models meta-analyses for all outcomes at all available time points (i.e., baseline, post-assessment, and follow-up). Outcome domains were analysed only if five or more relevant RCTs were available per outcome, in order to be consistent with previous EAGG meta-analyses [15, 33, 34] and to reduce between-SMD heterogeneity [35]. SMDs were combined using the inverse variance method [31, 36], and the presence of between-SMD heterogeneity was tested using *Q* – i.e., chi-squared test – and the magnitude of true heterogeneity relative to random heterogeneity was estimated using the *I*^*2*^ statistic [31].

We conducted pre-specified sensitivity analyses where at least five relevant trials met inclusion criteria. Separate analyses were conducted that included only trials where: (i) there was a semi-active comparator; ii) only a minority (i.e., <30%) of participants were receiving medication; iii) only WM was targeted; iv) multiple cognitive processes were targeted; v) participants were preselected based on impairment in the trained cognitive domain (e.g., WM, attention, inhibition); vi) with children/adolescents (<18-years-old); vii) with adults (>18-years); or viii) PBLIND assessment was conducted in the intervention setting. We ran pre-specified meta-regressions with the predictors: mean age (in trials with children/ adolescents only) or overall RoB (RoB defined as low, some concerns, or high according to the Cochrane RoB 2.0 Tool) [22]. Post-hoc meta-regressions with publication year as a predictor also tested whether SMD sizes reduced as study rigour improved over time. To conduct a meta-regression, there had to be at least 10 relevant trials per predictor. All the above analyses were pre-specified in our protocol. We also carried out three additional exploratory (not pre-specified) analyses in response to Reviewer comments. For the first, we conducted post-hoc sensitivity analyses excluding trials that were funded by companies with a commercial interest in the outcome of the RCT (i.e., Akili Interactive Labs Ltd, Kollins et al., 2020 [37]; Cogmed, Klingberg et al., 2005, Sandberg & McAuley 2021 [38, 39]; NeuroCog Solutions Pty Ltd [Australia], Johnstone et al., 2012 [40]; NeuroScouting, Meyer et al., 2020 [41]; Sincrolab, Medina et al., 2021 [42]). The findings did not change when these studies were excluded. For the second, we analysed the PBLIND outcomes recorded most proximally to the intervention setting. For the third, we added relevant outcomes from a paper by Kofler et al. (2020) [43] which compared two cognitive training interventions - Inhibitory Control Training (ICT) versus Central Executive Training (CET) – and so was judged not to meet our inclusion criteria. However, it was pointed out by the Reviewer (and confirmed by M. Kofler, via personal communication, 7th January 2023) that the ICT was designed as the comparator arm and CET the treatment arm for ADHD symptom outcomes and vice versa for motor inhibition outcomes.

Finally, publication bias was assessed using Egger’s regression test of small study effects and was carried out only for significant results with all trials included.

We used RevMan [36] to calculate SMDs and to run meta-analyses, Stata [44] to run meta-regressions, and Jamovi [45] to run publication bias analysis.

## Results

Of the 359 non-duplicate reports found, 233 were removed at title and abstract screening, leaving 136 to be assessed at full-text for eligibility. Of these, 94 reports were excluded (i.e., 36 used non-CCT; 17 had no control arm; 13 were non-randomised; 7 with no parallel arms; 7 had no ADHD diagnosis meeting our criteria; 3 were registered protocols; 2 re-analysed data already included in our meta-analysis; 2 included participants with non-eligible neurodevelopmental disorders; 2 did not share data; 2 did not report relevant data; 1 did not report participant eligibility criteria; 1 was a conference poster; and 1 did not answer our data request). This left 42 eligible reports derived from 36 RCTs (PRISMA Flowchart, see Supplementary Fig. 1; included reports, see Supplementary Table 3; excluded reports with reasons, see Supplementary Table 4) giving a total sample of 2234 participants, double the number of RCTs and triple the sample size reported in Cortese et al. (2015) (RCTs=16; sample=759). Of those 36 RCTs, the most common comparator was a semi-active control (*n* = 22), followed by WLC (*n* = 11) and TAU (*n* = 3). Most trials recruited children (5–12-years-old; *n* = 26), followed by adults (>18-years; *n* = 8), and adolescents (13-18-years-old; *n* = 2) (see Supplementary Table 2). Most trials evaluated working memory training (WMT) (*n* = 17), followed by multi-process training (MPT) (*n* = 13), attention training (*n* = 5), and inhibitory control training (*n* = 1). Most trials administered CCT at home (*n* = 21), followed by at school (*n* = 5), in a laboratory setting (*n* = 3), the clinic/hospital (*n* = 2), and a mixed setting (i.e., home, lab, library, clinic, school; *n* = 5). Several outcomes were excluded from five out of the 36 eligible RCTs. We excluded i) independent evaluator-rated ASRS from Virta et al (2010) [46] because the author had no access to data; ii) ADHD-RS from Bikic et al. (2017) [47] because total scores were conflated with oppositional defiant disorder symptoms; subscales were unavailable following personal communication; iii) Conners’ rating scale or CBCL from Johnstone et al. (2012; 2010) [40, 48] because they were not reported; the author declined to share data following personal communication; iv) Conners’ rating scale, counting span, digit span, GNG Task from Johnstone et al. (2012) [40] because they were not reported; the author declined to share data following personal communication; and v) ADHD-RS and TOVA from Kollins et al. (2020) [37] because only data from all available cases at baseline and post-assessment or change scores were reported, so no effect size could be calculated. The required data were only accessible via Akili Interactive Labs, Inc (Boston, MA, USA), who declined to share data following personal communication. Of the 36 RCTs assessed, overall RoB was judged “high-risk” in 25 and as being of “some concerns” in 10 RCTs (see Supplementary Fig. 2). These judgments were driven mainly by failures to conceal group assignment to the outcome assessor (Domain 4, 17 out of 36 RCTs) or because they had high levels of missing outcome data (Domain 3, 14 out of 36 reported >20% drop-outs). Regarding bias in selection of the reported results (Domain 5), 32 RCTs were judged to be of ‘some concern’ as these did not pre-register a trial protocol and/or statistical analysis plan. Bias arising from poor randomisation processes (Domain 1) or due to deviations of allocation to the intended intervention (Domain 2) were rated infrequent across most RCTs.

### Post-treatment outcomes

#### ADHD symptoms

**Fig. 1:**
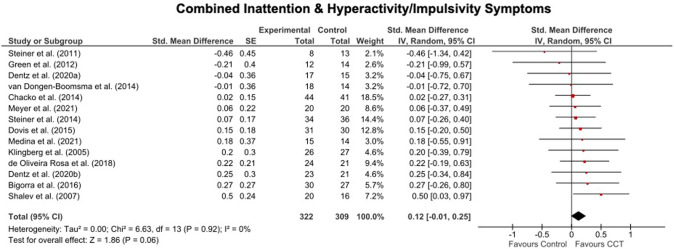
Forest plots for meta-analysis of effects of PBLIND outcome measures of ADHD total symptoms. Note. CCT Computerized Cognitive Training, SE Standard Error, Std. Standardised.

**Fig. 2:**
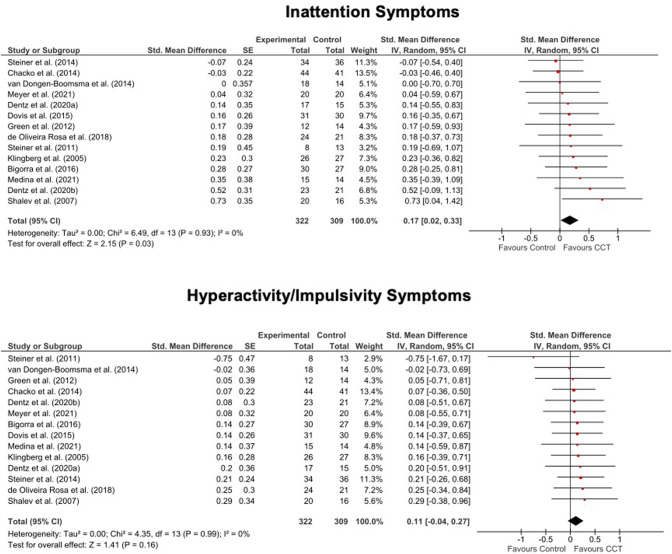
Forest plots for meta-analysis of effects PBLIND outcome measures of Inattention or Hyperactivity/Impulsivity symptoms. Note. CCT Computerized Cognitive Training, SE Standard Error, Std. Standardized.

Of the 36 included trials, 14 reported PBLIND ADHD outcomes (24 reported MPROX outcomes; for MPROX/PBLIND outcomes, see Supplementary Table 5; for MPROX results, see Supplementary Table 6 and Supplementary Fig. 3 and 4). There were small and marginally significant improvements favouring CCT relative to control in inattention (SMD = 0.17, 95%CI[0.02 to 0.33), but no significant effects were seen for ADHD total (SMD = 0.12, 95%CI[−0.01to 0.25]) or hyperactivity/impulsivity (SMD = 0.11, 95%CI[−0.04 to 0.27]) symptoms. The improvement in inattention symptoms remained significant when analyses were restricted to trials with semi-active controls (SMD = 0.20, 95%CI[0.04 to 0.37]), and doubled in size when restricted to PBLIND outcomes measured in the intervention setting (SMD = 0.40, 95%CI[0.09 to 0.71]). MPT was equivalent to WMT (MPT, SMD range=0.11 to 0.12, 95%CI range [−0.15 to 0.38]); WMT, SMD range = 0.08 to 0.17, 95%CI range [−0.05 to 0.39]). Heterogeneity was low and non-significant in all analyses (see Figs. 1 and 2, Table 1).

**Table 1:** Summary of results showing pooled standardized mean differences (SMD; with Hedges’ *g* adjustment) between treatment and control arms for PBLIND measures of ADHD symptoms at the first assessment after the final CCT session. Significant values are bolded.

				Effect size estimate			Heterogeneity	
Outcome	Trials Included	Study *N*	Total *N*	SMD	95%CI	*p*	I^2^	p*
*ADHD Symptoms*								
ADHD Total	All	14	631	0.12	−0.01 to 0.25	0.06	0	0.92
	Semi-active control	13	561	0.13	−0.01 to 0.27	0.07	0	0.89
	MED	6	324	0.16	−0.02 to 0.34	0.07	0	0.66
	WMT	7	329	0.08	−0.12 to 0.27	0.43	0	0.93
	MPT	5	226	0.11	−0.09 to 0.31	0.27	0	0.73
	Children/adolescents	13	587	0.11	−0.02 to 0.24	0.08	0	0.89
	Adults	1	na	na	na	na	na	na
	PBLIND assessed in intervention setting	5	167	0.22	−0.05 to 0.50	0.12	0	0.54
	PBLIND not assessed in intervention setting	9	464	0.09	−0.05 to 0.23	0.20	0	0.94
	Non-commercially funded	11	509	0.12	−0.02 to 0.26	0.09	0	0.78
Inattention	All	14	631	**0.17**	**0.02 to 0.33**	**0.03**	0	0.93
	Semi-active control	13	561	**0.20**	**0.04 to 0.37**	**0.02**	0	0.95
	MED	6	258	0.29	−0.04 to 0.41	0.11	0	0.58
	WMT	7	329	0.17	−0.05 to 0.39	0.13	0	0.86
	MPT	5	226	0.12	−0.14 to 0.38	0.37	0	0.90
	Children/adolescents	13	587	0.15	−0.02 to 0.31	0.08	0	0.95
	Adults	1	na	na	na	na	na	na
	PBLIND in intervention setting	5	167	**0.40**	**0.09 to 0.71**	0.01	0	0.74
	PBLIND not assessed in intervention setting	9	464	0.09	−0.09 to 0.28	0.32	0	0.99
	Non-commercially funded	11	509	0.17	−0.01 to 0.34	0.06	0	0.81
Hyperactivity/ Impulsivity	All	14	631	0.11	−0.04 to 0.27	0.16	0	0.99
	Semi-active control	13	561	0.1	−0.07 to 0.27	0.24	0	0.98
	MED	6	324	0.13	−0.10 to 0.35	0.27	0	0.99
	WMT	9	405	0.11	−0.08 to 0.31	0.26	0	1.00
	MPT	5	226	0.11	−0.15 to 0.28	0.41	0	0.44
	Children/adolescents	13	587	0.12	−0.05 to 0.28	0.17	0	0.98
	Adults	1	na	na	na	na	na	na
	PBLIND in intervention setting	5	167	0.15	−0.15 to 0.46	0.33	0	0.99
	PBLIND not assessed in intervention setting	9	464	0.10	−0.09 to 0.28	0.29	0	0.86
	Non-commercially funded	11	509	0.11	−0.07 to 0.29	0.22	0	0.93

*ADHD* attention-deficit/hyperactivity disorder, *CI* Confidence Intervals, *I*^*2*^ percentage of between-study variation across SMDs that is due to heterogeneity rather than chance, *MED* only a minority (i.e., <30%) of participants were receiving medication, *MPT* multi-process training, *N* sample size, *PBLIND* probably blinded, *SMD* Hedges’ g, *WMT* working memory training.

^*^p-values from Q – i.e., the chi-squared test statistic.

#### Neuropsychological outcomes

##### Ratings

Executive functioning based on the Global Executive Composite of the Behaviour Rating Inventory of Executive Function (BRIEF) were rated by PBLIND assessors in only four trials and MPROX assessors in thirteen trials (see Supplementary Table 4). MPROX outcomes showed no benefit for CCT. Heterogeneity was non-significant (see Supplementary Table 6 and Supplementary Fig. 5).

##### Laboratory measures

Thirty-two trials included at least one laboratory measure of neuropsychological outcomes (see Supplementary Table 8). We summarise results from all analyses below.

##### WM outcomes

**Fig. 3:**
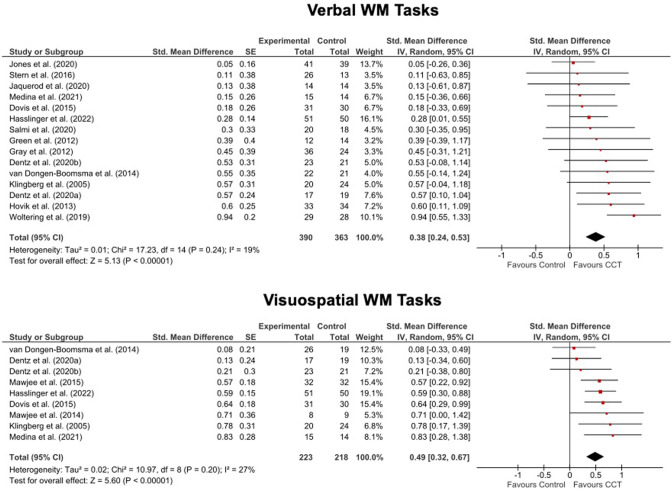
Forest plots for meta-analysis of effects on verbal and visual-spatial short-term and working memory tasks. CCT Computerized Cognitive Training, SE Standard Error, Std. Standardized.

Outcomes were based on forward and backward versions of verbal and visuospatial span tasks. Preliminary analyses showed that our findings did not change whether analysing scores from each version separately or in aggregate, so for simplicity we report only the aggregated scores below. Results for both verbal and visuospatial WM tasks favoured CCT with highly significant but mainly moderate effects across the board. Effects were generally larger for visuospatial than verbal WM. Heterogeneity was low-to-moderate but non-significant (see Table 2 and Fig. 3).

**Table 2:** Summary of results showing pooled standardized mean differences (SMD; with Hedges’ *g* adjustment) between treatment and control arms for laboratory measures of neuropsychological and academic outcomes measured at the first assessment after the final CCT session. Significant values are bolded.

				Effect size estimate			Heterogeneity	
Outcome	Trials Included	Study N	Total N	SMD	95%CI	*p*	I^2^	p*
*Neuropsychological*								
Attention	All	17	836	0.14	−0.04 to 0.32	0.13	**39**	**0.05**
	Semi-active control	12	500	0.14	−0.05 to 0.32	0.14	6	0.38
	MED	6	297	0.04	−0.22 to 0.30	0.76	18	0.29
	WMT	9	543	0.05	−0.12 to 0.23	0.56	5	0.39
	MPT	6	226	0.36	−0.08 to 0.80	0.11	**60**	**0.03**
	Children/adolescents	14	701	0.1	−0.10 to 0.30	0.32	36	0.11
	Adults	3	na	na	na	na	na	na
	Non-commercially funded	15	765	0.08	−0.08 to 0.23	0.33	14	0.30
Interference Inhibition	All	8	356	0.10	−0.32 to 0.51	0.65	**73**	**<0.001**
	Semi-active control	7	289	0.12	−0.38 to 0.61	0.65	**77**	**<0.001**
	MED	4	na	na	na	na	na	na
	WMT	4	na	na	na	na	na	na
	MPT	4	na	na	na	na	na	na
	Children/adolescents	7	326	−0.05	−0.40 to 0.31	0.80	**61**	**0.02**
	Adults	1	na	na	na	na	na	na
	Non-commercially funded	5	242	0.13	−0.38 to 0.64	0.61	**73**	**0.005**
Motor Inhibition	All	15	827	0.15	−0.03 to 0.33	0.10	37	0.07
	Semi-active control	11	533	**0.24**	**0.05 to 0.43**	**0.01**	13	0.32
	MED	6	315	0.18	−0.09 to 0.46	0.19	30	0.21
	WMT	8	520	0.14	−0.04 to 0.33	0.13	13	0.33
	MPT	6	272	0.15	−0.27 to 0.57	0.49	65	0.01
	Children/adolescents	12	692	0.18	−0.02 to 0.39	0.08	44	0.5
	Adults	3	na	na	na	na	na	na
	Non-commercially funded	14	798	0.12	−0.06 to 0.29	0.18	32	0.12
Non-Verbal Reasoning	All	6	312	0.05	−0.17 to 0.28	0.63	0	0.92
	Semi-active control	6	312	0.05	−0.17 to 0.28	0.63	0	0.92
	MED	3	na	na	na	na	na	na
	WMT	5	251	0.07	−0.18 to 0.31	0.59	0	0.85
	MPT	1	na	na	na	na	na	na
	Children/adolescents	5	268	0.05	−0.19 to 0.29	0.7	0	0.85
	Adults	1	na	na	na	na	na	na
	Non-commercially funded	4	268	0.03	−0.21 to 0.27	0.83	0	0.91
Processing Speed	All	8	393	−0.18	−0.40 to 0.03	0.1	10	0.35
	Semi-active control	5	166	−0.33	−0.73 to 0.06	0.1	35	0.19
	MED	3	na	na	na	na	na	na
	WMT	3	na	na	na	na	na	na
	MPT	4	na	na	na	na	na	na
	Children/adolescents	6	302	−0.15	−0.38 to 0.08	0.19	0	0.73
	Adults	2	na	na	na	na	na	na
	Non-commercially funded	7	364	−0.16	−0.40 to 0.07	0.17	17	0.30
Set-Shifting	All	5	247	0.22	−0.09 to 0.53	0.17	30	0.22
	Semi-active control	4	na	na	na	na	na	na
	MED	4	na	na	na	na	na	na
	WMT	2	na	na	na	na	na	na
	MPT	2	na	na	na	na	na	na
	Children/adolescents	4	na	na	na	na	na	na
	Adults	1	na	na	na	na	na	na
	Non-commercially funded	0	na	na	na	na	na	na
Verbal WM	All	15	753	**0.38**	**0.24 to 0.53**	**<0.001**	19	0.24
	Semi-active control	12	528	**0.28**	**0.12 to 0.44**	**<0.001**	0	0.83
	MED	5	253	**0.31**	**0.05 to 0.57**	**0.02**	0	0.86
	WMT	12	624	**0.43**	**0.26 to 0.60**	**<0.001**	28	0.17
	MPT	2	na	na	na	na	na	na
	Children/adolescents	10	547	**0.31**	**0.16 to 0.45**	**<0.001**	0	0.62
	Adults	5	206	**0.49**	**0.13 to 0.84**	**0.007**	42	0.12
	Non-commercially funded	13	680	**0.39**	**0.23 to 0.55**	**<0.001**	25	0.19
Visuospatial WM	All	9	441	**0.49**	**0.31 to 0.67**	**<0.001**	27	0.20
	Active control	6	247	**0.40**	**0.14 to 0.65**	**0.002**	38	0.16
	MED	3	na	na	na	na	na	na
	WMT	7	351	**0.43**	**0.23 to 0.63**	**<0.001**	29	0.20
	MPT	2	na	na	na	na	na	na
	Children/adolescents	6	316	**0.49**	**0.25 to 0.73**	**<0.001**	48	0.09
	Adults	3	na	na	na	na	na	na
	Non-commercially funded	7	368	**0.44**	**0.25 to 0.63**	**<0.001**	29	0.91
*Academic*								
Arithmetic ability	All	9	516	0	−0.11 to 0.11	0.98	0	0.73
	Semi-active control	5	295	0.1	−0.13 to 0.33	0.4	0	0.62
	MED	na	na	na	na	na	na	na
	WMT	7	430	0.07	−0.12 to 0.26	0.48	0	0.81
	MPT	na	na	na	na	na	na	na
	Children/adolescents	8	452	0.00	−0.11 to 0.11	0.99	0	0.63
	Adults	1	na	na	na	na	na	na
	Non-commercially funded	8	476	0.01	−0.11 to 0.12	0.93	0	0.65
Reading comprehension	All	8	450	0.18	−0.01 to 0.36	0.06	0	0.43
	Semi-active control	6	343	0.12	−0.09 to 0.33	0.27	0	0.51
	MED	3	na	na	na	na	na	na
	WMT	7	414	0.14	−0.05 to 0.34	0.15	0	0.46
	MPT	0	Na	na	na	na	na	na
	Children/adolescents	8	450	0.18	−0.01 to 0.36	0.06	0	0.43
	Adults	0	na	na	na	na	na	na
	Non-commercially funded	7	410	0.19	−0.02 to 0.40	0.07	0.35	11
Reading fluency	All	7	445	0.03	−0.06 to 0.11	0.55	0	0.55
	Semi-active control	2	na	na	na	na	na	na
	MED	na	na	na	na	na	na	na
	WMT	6	395	0.05	−0.15 to 0.25	0.59	0	0.43
	MPT	na	na	na	na	na	na	na
	Children/adolescents	6	381	0.04	−0.05 to 0.13	0.44	0	0.58
	Adults	1	na	na	na	na	na	na
	Non-commercially funded	6	405	0.02	−0.07 to 0.11	0.64	0	0.53

*ADHD* attention-deficit/hyperactivity disorder, *CI* Confidence Intervals, *I*^*2*^ percentage of between-study variation across SMDs that is due to heterogeneity rather than chance, *MED* only a minority (i.e., <30%) of participants were receiving medication, *MPT* multi-process training, *N* sample size, *SMD* Hedges’ *g*, *WM* working memory, *WMT* working memory training.

^*^p-values from Q – i.e., the chi-squared test statistic.

##### Other neuropsychological outcomes

There were no benefits of CCT for measures of attention, interference control, non-verbal reasoning, processing speed, or set-shifting either in analysis with all trials or in the sensitivity analyses. Heterogeneity was significant for attention and interference control. For motor inhibition there was significant benefit of CCT but only in the subsample of trials with a semi-active control and with <30% medicated participants. Heterogeneity was non-significant (see Table 2 and Supplementary Fig. 5).

#### Academic outcomes

There were no significant effects of CCT on any academic outcome (trials: arithmetic ability, *n* = 9; reading comprehension, *n* = 8; reading fluency, *n* = 7). Heterogeneity was zero and non-significant (see Table 2, Supplementary Table 8, and Supplementary Fig. 6).

#### Meta-regression

Effect size estimates were not significantly predicted by mean age, publication year, or overall RoB for any outcome except for neuropsychological measures of attention where SMDs got smaller over time (see Supplementary Table 10).

#### Adult findings

Only eight trials enrolled adult samples. We only had sufficient trials to analyse MPROX self-ratings of ADHD total symptoms (trials, *n* = 5) or laboratory measures of verbal WM (trials, *n* = 5), with only the latter showing a benefit in favour of CCT (SMD = 0.49, 95%CI[0.13–0.84]) (see Table 2).

### Follow-up outcomes

Eight trials reported follow-up outcomes (mean, 6-months; range, 3–6-months), all of which recruited children aged 9–12-years. Due to an insufficient number of trials, it was not possible to analyse PBLIND outcomes in any one domain or laboratory measures beyond motor inhibition, attention, verbal WM, and reading comprehension. A significant and small benefit favouring CCT relative to control was found in the analysis of MPROX ratings of executive functions (but not ADHD symptoms) (*n* = 6; SMD = 0.24, 95%CI[0.02–0.45]); laboratory measures of motor inhibition (*n* = 7; SMD = 0.24, 95%CI[0.05–0.43]) and verbal WM (*n* = 6; SMD = 0.32, 95%CI[0.15–0.49]), and reading comprehension only (*n* = 5; SMD = 0.26, 95%CI[0.00–0.52]). Heterogeneity was small and non-significant in all cases (see Supplementary Table 6-7, Supplementary Figs. 4-6).

### Publication bias

There was no evidence of publication bias in any analysis (see Supplementary Fig. 7).

### Additional exploratory analyses

#### PBLIND measured most proximally to intervention setting

This involved replacing most distal PBLIND outcomes, with most proximal PBLIND outcomes in eight trials. Notably, this did not materially change the interpretation of the findings of any of our previous analyses (for results, see Supplementary Table 11).

#### Including data from Kofler et al. (2020)

When Kofler et. al. 2020 PBLIND parent ratings were added, the ADHD total symptoms became marginally significant (SMD = 0.13, 95%CI [0.01 to 0.25]) while the significant effects for inattention symptoms increased slightly, remaining significant (SMD = 0.18, 95%CI[0.03 to 0.34]). The intervention effect for hyperactivity/impulsivity remained non-significant (SMD = 0.12, 95%CI[−0.03 to −0.27]). In these exploratory analyses effects sizes remained small.

## Discussion

This comprehensive and well-powered meta-analysis found no evidence to support the use of CCT in its current forms as a *stand-alone* treatment for core ADHD symptoms. In our pre-registered analyses, effects on PBLIND measures of core ADHD features were limited to a significant, but small, short-term effect on inattention symptoms, which was substantially unchanged with variations in experimental control arm. This finding has possible clinical relevance given that inattention may become a more important issue in adulthood [6]. However, the small size of the effect suggested limited clinical significance when compared to the size of short-term effects of methylphenidate on ADHD core symptoms reported in a recent meta-analysis (SMD range = −0·49 to −0.82, 95%CI range [−1.16 to −0·62]) [5]. In general, the effects reported in this meta-analysis were smaller than in our previous meta-analyses – with, for instance, the SMD for PBLIND inattention symptoms dropping from 0.32 in 2015 to 0.17 in this analysis [14, 15]. Equivalent drops in MPROX ratings reported in our supplementary analysis section (0.47 to 0.27, respectively) suggest that even trials with inadequately blinded outcomes now provide little support for the use of CCT as a treatment for ADHD symptoms. The drop in effect size could be the result of better blinding by design in more recent trials, but there are other possible explanations. For instance, our update excluded non-computerized cognitive training and non-validated measures of ADHD symptoms to improve homogeneity of included studies and outcomes, which meant two relatively large effects included in our 2015 paper were not carried forward to our update (SMD = 0.82 [49] and SMD = 0.98 [48]). Positive effects seen on visuospatial and verbal WM performance did not transfer to other neuropsychological processes and/or academic outcomes and in some cases were no longer statistically significant when analyses were limited to trials with semi-active control arms.

The scale of the current meta-analysis - made possible by the large number of new trials published in recent years - provided scope to explore a range of aspects of the effects of CCT for ADHD not possible before. First, against expectation, we found no evidence that MPT was superior to WMT when PBLIND outcomes were employed. Cortese et al. [15] had found a moderate-to-large effect of MPT on MPROX outcomes. This led us to hypothesize that, consistent with the notion that ADHD is a neuropsychologically heterogeneous condition, interventions that target a range of different cognitive processes would be more effective at the overall group level because some sub-groups of people with ADHD would benefit from one form of training, while others would benefit from a different one. Second, our analysis of trials where PBLIND measures collected in the same setting in which the intervention was delivered (e.g., both at school) produced effects sizes that were twice the size than those seen for the analysis of all trials and four times the size of the analysis when PBLIND assessments were collected in a different setting to the intervention (e.g., at home versus at school). The latter was by far the most common arrangement but confounded “blindedness” of ratings with setting. By disentangling this confound in the current meta-analysis, we provide evidence that CCT may produce significant effects of moderate size with minimal heterogeneity, but this improvement is likely to be setting-specific with little generalisation beyond the training setting to other settings. Given the small sample of RCTs (*N* = 5) in this sensitivity analysis we encourage future trials to explore this issue systematically by varying blinded clinical outcomes measured in the same or different setting to the intervention.

Third, with respect to evidence of the longer-term benefits of CCT, positive effects on WM waned and became non-significant for some outcomes. However, in contrast, there were a number of outcomes where significant, though still small, effects emerged only at follow-up – e.g., objective measures of motor inhibition and reading comprehension and MPROX ratings of executive functioning. These later effects, if confirmed, might represent “sleeper effects” – i.e., effects of CCT become consolidated over time. Such an interpretation remains speculative at this point given the limited number of high risk-of-bias trials on which the findings are based. One other explanation could be that the short-term effects found in WM were in fact later transferred to a positive effect on associated functions (e.g., academic skills) later down the line. These tentative explanations could be a focus in future research along with investigating whether “top-up” interventions are required to prolong the short-term benefit we found.

Fourth, as previously reported in our 2015 paper [15], WMT (implemented in 17 out of 36 trials) had limited benefits, despite being the most widely available commercial form of CCT. It did not significantly reduce PBLIND measures of ADHD symptoms, while its effects on WM were no greater than for other training types and there was no evidence of generalisation to other outcomes. WMT in people with ADHD appears to produce practice-like gains with little transfer away from the trained cognitive domain.

Fifth, there were too few relevant trials to draw any strong conclusions about the value of CCT for adults with ADHD. One speculation is that the modest, short-term improvements found for inattention symptoms, but not combined ADHD, in children and adolescents might indicate possible benefits to adults for whom inattention becomes especially relevant. However, this would need to be tested specifically in future trials.

Sixth, the three exploratory analyses requested during the review process – i.e., restricting analysis to non-commercially funded trials, analysing the PBLIND data collected most proximally rather than most distally, and adding PBLIND parent-rated outcomes from Kofler et al. (2020) – did not change our interpretation of the results, highlighting the robustness of our analyses.

We believe that the translational logic of targeting neuropsychological processes thought to mediate ADHD pathophysiology to improve symptoms and/or neuropsychological processes with related improvement in functioning still has viability. However, given the lack of results detailed above, maybe new and innovative training approaches will be required to move the field forward. These may involve targeting different processes, using more focused strategies or different intervention modalities. With regard to the focused strategies, greater benefits may be derived by targeting more basic non-executive functions, like neurophysiological processes that regulate arousal/motivation or emotions [8], which are thought to function atypically in ADHD and are potentially related to symptom expression and cognitive impairment [50]. Support for this approach might come in the form of our finding that performance on forward and backward versions of verbal or visual span tasks were comparably improved with CCT, which – given that these types of memory are underpinned by processes differing in executive demand – would suggest that the impact of CCT may be on more basic non-executive functioning. With regard to different intervention modalities, two options should be explored. First, given that at the individual level there is considerable neuropsychological heterogeneity that might dilute the group level impact of CCT, the first option would be to more precisely match the training to the needs of the patients (e.g., WMT could be given to those with WM difficulties at baseline). However, the evidence from studies investigating moderating/mediating factors for this is limited and conflicting [51–53], and we were unable to explore the impact of baseline cognitive performance on the CCT treatment effect as only one study screened based on impairment in the trained cognitive domain at baseline [46]. Second, because there is greater plasticity earlier in development, the second option could be to focus training on younger age groups than those currently studied (typically between 8 and 14-years or older). For instance, preliminary work has shown gains in neuropsychological and some degree of reduction in ADHD symptoms using play-based interventions for pre-schoolers that train neurocognitive and behavioural domains in real-world settings [54, 55]. Although we aimed to measure the effect of CCT in pre-schoolers with ADHD, there were no RCTs with samples of children with a confirmed ADHD diagnosis. We may also want to focus more on ecologically valid ways of training neuropsychological functioning, and focus on parents/caregivers and teachers as builders of functioning capacities [56]. Finally, approaches that more directly target brain processes are often assumed to be more effective. However, recent meta-analysis do not support the value of either neurofeedback [33] or non-invasive brain stimulation techniques (e.g., repetitive transcranial magnetic stimulation, transcranial direct current stimulation) [57] as ADHD interventions. It may be that our translation model is wrong and improvements in neuropsychological functions and symptoms in fact occur independently, and that CCT effects are going to be limited to the cognitive domain and/or related functional or educational outcomes [2, 58].

There are several limitations to our meta-analysis resulting from the studies available for inclusion. First, the majority of RCTs suffered from biases that were judged to be of some or high risk. For example, high levels of attrition were common - 17 out of 42 reports had dropouts >20% at post-assessment but did not account for this analytically – which may have inflated SMDs. Further, while 14 RCTs reported PBLIND ADHD outcomes, the remaining 22 used WLC/TAU controls and/or MPROX outcomes only, meaning that blinding was unlikely or practically impossible. This raised the risk that outcome assessors were biased by knowledge of group assignment. Future RCTs should improve participant retention or provide evidence that results were not biased by missing data (e.g., running sensitivity analyses to test whether results changed under plausible assumptions about the relationship between missingness in the outcome and its true value) [22]. They should also adopt double-blinded designs and active controls identical to the active intervention but without an adaptive component of the targeted function, while also avoiding making tasks boring and demotivating. This is important as this component is central to the translational logic of CCT [8, 13]. Second, very few studies focused on non-childhood samples, meaning that we could not test whether our findings generalized to pre-school, adolescent, or adult samples. Third, our meta-analyses aggregated data from the group level, which can obscure variability of effects at the level of the individual. We cannot rule out that meta-analyses of individual patient data might provide greater insights into possible moderators of CCT effects to develop more personalized CCT programs. Forth, unfortunately, due to an insufficient number of trials (i.e., less than 5) using MPT, we could not explore whether MPT is superior to WMT in analyses of several of the neuropsychological outcomes (e.g., interference inhibition, processing speed, set-shifting) as well as all academic outcomes, so it remains for future researchers to explore these matters. Sixth, the limited evidence of CCT efficacy we found raises important questions about iatrogenic effects, cost-benefits, and opportunity costs of CCT, none of which were considered in the included studies. Future studies should address these matters. Seventh, given the translational logic of CCT, one would expect CCT effects to generalise to functional impairments, but only a minority of studies measured outcomes beyond ADHD symptoms and/or neuropsychological outcomes, leaving open the possibility that future studies may show that CCT can yield improvements in everyday functioning or quality of life. Fifth, only seven trials tested effects over the longer-term. Eighth, one possible limitation is that for one trial identified as meeting inclusion criteria [37], we were unable to calculate effect size estimates for ADHD-RS and TOVA outcomes because the sponsor (Akili Interactive Labs, Inc) declined to share data for complete cases at each assessment time-point. This trial is noteworthy given its high quality – e.g., unlike any other included study, it had a very large sample (*N* = 348) with a pre-registered trial protocol and analysis plan – so inclusion of these outcomes may have changed our conclusions. The authors found that compared to an semi-active control, CCT targeting attention and cognitive control (i.e., AKL-T01) led to no improvement in blinded-parent ADHD-RS ratings of ADHD symptoms and BRIEF ratings of executive functioning, but significantly improved TOVA performance – a task-based measure of attention. These results point to potential practice-like gains with transfer limited to the trained cognitive domain, supporting the main conclusions from our meta-analyses. Further, given the size of this study, if ADHD-RS outcomes were included in our primary analysis of PBLIND outcomes, it would likely substantially diminish or even abolish the significant CCT-related improvement in inattention symptoms we found, further supporting our conclusion of the limited clinical benefit of CCT as a standalone treatment for ADHD. We agree with the authors, however, that further investigation into the real-world benefit of AKL-T01 is worthwhile. Finally, our protocol precluded trials with head-to-head comparisons of different interventions. Future analyses should focus on these.

## Conclusion

There was no empirical support for the use of CCT as a stand-alone intervention for ADHD symptoms based on the largest and most comprehensive meta-analysis of RCTs conducted to date. Small effects, of likely limited clinical importance, on inattention symptoms were found – but these were limited to the setting in which the intervention was delivered. Robust evidence of small-to-moderate improvements in visual-spatial and verbal STM/WM tasks did not transfer to other domains of executive functions or academic outcomes, but these might take time to become apparent. New interventions targeting different processes using different, more ecologically valid, approaches within more focused intervention strategies are required going forward.

## Supplementary information

Supplementary Material
