# Supplementary material for: Computerized cognitive training in attention-deficit/hyperactivity disorder (ADHD): a meta-analysis of randomized controlled trials with blinded and objective outcomes
Source: Mol Psychiatry. 2023 Mar 29;28(4):1402–14. doi: 10.1038/s41380-023-02000-7 (PMC10208955; doi:10.1038/s41380-023-02000-7)
Supplement: Supplementary file 1 — Supplementary Material [file 41380_2023_2000_MOESM1_ESM.docx]

Supplemental Data

# List of deviations from the pre-specified protocol and Cortese et al. (1) (hereafter Cortese et al.)

Below we list differences from Cortese et al. and deviations from our pre-registered protocol (ID: CRD42021229279), all of which were aimed at ensuring the most accurate and unbiased effect size estimates possible were calculated:

1. We only included validated measures of ADHD symptoms, which meant two effects reported by Johnstone et al. (2,3) were not carried forward from Cortese et al. as they were purpose-built, unvalidated ADHD symptom checklists.
2. We did not include inattention or hyperactivity/impulsivity subscales in analysis of ADHD Total symptoms unless subscales were combined to create an overall score of ADHD Total symptoms. This meant two effects based on inattention subscales from two studies (4,5) were not included in proximally blinded outcomes of ADHD Total symptoms.
3. Cortese et al. judged teacher-rated outcome measures of ADHD symptoms reported by van der Oord et al. (6) to be probably blinded. However, following personal correspondence with Prof van der Oord (31/08/21), we discovered that parents, teachers, participants, and therapists were in fact not blinded. This meant we did not carry forward any outcomes judged to be probably blinded by Cortese et al.
4. We discovered that in Cortese et al. in the analysis of outcomes measuring arithmetic academic the signs of effect size estimates were coded in the opposite direction to what they should have been. This error has now been correct in this update.
5. Following personal correspondence, we have received data from Green et al et al (2012; 03/10/21) that was unavailable at the time to Cortese et al. and is included in our update, which includes raw data for CPRS DSM-Inattention, DSM Hyperactivity/Impulsivity subscales, WISC-IV Digit Span Task.
6. In Cortese et al., the 95%CIs based on the combined parent ADHD-RS Inattention and Hyperactivity-Impulsivity subscales reported in Shalev et al. (2006) were incorrectly calculated. This has been corrected in the update.
7. Unlike Cortese et al., this update only included computerised cognitive training, which meant the exclusion of (7).
8. When available, we included self-ratings unless completed by children or adolescents, as these were deemed less reliable than self-ratings based on adults.
9. We planned run a sensitivity analysis excluding trials that recruited participants based on impairment in the trained neuropsychological domain (e.g., working memory, attention, inhibition) as well as ADHD. As this only applied to two trials(8,9), we decided not to run this sensitivity analysis as it would not be informative.
10. We planned to run a sensitivity analysis excluding trials enrolling only children (<12 years); adolescents (12- to 18-years), and adults (>18 years). However, as only three trials included adolescent participants(10–12), we decided to combine trials with children and adolescents.
11. In our pre-registered protocol, we planned to measure the effects of CCT on oppositional deficient disorder or conduct disorder symptoms. There were insufficient trials (i.e., <5) to conduct an analysis.
12. We pre-registered a sensitivity analysis wherein were analysed trials where the PBLIND measure is derived from the same source as MPROX to explore the potential confounding between blinding and setting. For completeness, we ran additional sensitivity analysis where the PBLIND measures was measured in a setting that was different to the intervention setting.

| **Supplemental Table 1. PRISMA 2020 Checklist** | | | |
| --- | --- | --- | --- |
| **Section and Topic** | **Item #** | **Checklist item** | **Location where item is reported** |
| **TITLE** | | |  |
| Title | 1 | Identify the report as a systematic review. | Title page |
| **ABSTRACT** | | |  |
| Abstract | 2 | See the PRISMA 2020 for Abstracts checklist. | Pg. 1 |
| **INTRODUCTION** | | |  |
| Rationale | 3 | Describe the rationale for the review in the context of existing knowledge. | Pg. 2-5 |
| Objectives | 4 | Provide an explicit statement of the objective(s) or question(s) the review addresses. | Pg. 4-5 |
| **METHODS** | | |  |
| Eligibility criteria | 5 | Specify the inclusion and exclusion criteria for the review and how studies were grouped for the syntheses. | Pg. 5-6 |
| Information sources | 6 | Specify all databases, registers, websites, organisations, reference lists and other sources searched or consulted to identify studies. Specify the date when each source was last searched or consulted. | Pg. 6, Supplement |
| Search strategy | 7 | Present the full search strategies for all databases, registers and websites, including any filters and limits used. | Pg. 6-7, Supplement |
| Selection process | 8 | Specify the methods used to decide whether a study met the inclusion criteria of the review, including how many reviewers screened each record and each report retrieved, whether they worked independently, and if applicable, details of automation tools used in the process. | Pg. 6-8 |
| Data collection process | 9 | Specify the methods used to collect data from reports, including how many reviewers collected data from each report, whether they worked independently, any processes for obtaining or confirming data from study investigators, and if applicable, details of automation tools used in the process. | Pg. 7-9 |
| Data items | 10a | List and define all outcomes for which data were sought. Specify whether all results that were compatible with each outcome domain in each study were sought (e.g. for all measures, time points, analyses), and if not, the methods used to decide which results to collect. | Pg. 7-9 |
|  | 10b | List and define all other variables for which data were sought (e.g. participant and intervention characteristics, funding sources). Describe any assumptions made about any missing or unclear information. | Pg. 7-11, Supplement |
| Study risk of bias assessment | 11 | Specify the methods used to assess risk of bias in the included studies, including details of the tool(s) used, how many reviewers assessed each study and whether they worked independently, and if applicable, details of automation tools used in the process. | Pg. 7-8 |
| Effect measures | 12 | Specify for each outcome the effect measure(s) (e.g. risk ratio, mean difference) used in the synthesis or presentation of results. | Pg. 9-11 |
| Synthesis methods | 13a | Describe the processes used to decide which studies were eligible for each synthesis (e.g. tabulating the study intervention characteristics and comparing against the planned groups for each synthesis (item #5)). | Pg. 9-11 |
|  | 13b | Describe any methods required to prepare the data for presentation or synthesis, such as handling of missing summary statistics, or data conversions. | Pg. 9-11 |
|  | 13c | Describe any methods used to tabulate or visually display results of individual studies and syntheses. | Pg. 9-11 |
|  | 13d | Describe any methods used to synthesize results and provide a rationale for the choice(s). If meta-analysis was performed, describe the model(s), method(s) to identify the presence and extent of statistical heterogeneity, and software package(s) used. | Pg. 9-11 |
|  | 13e | Describe any methods used to explore possible causes of heterogeneity among study results (e.g. subgroup analysis, meta-regression). | Pg. 9-11 |
|  | 13f | Describe any sensitivity analyses conducted to assess robustness of the synthesized results. | Pg. 9-11 |
| Reporting bias assessment | 14 | Describe any methods used to assess risk of bias due to missing results in a synthesis (arising from reporting biases). | Pg. 7-8 |
| Certainty assessment | 15 | Describe any methods used to assess certainty (or confidence) in the body of evidence for an outcome. | Pg. 7-8 |
| **RESULTS** | | |  |
| Study selection | 16a | Describe the results of the search and selection process, from the number of records identified in the search to the number of studies included in the review, ideally using a flow diagram. | Pg. 11-15 |
|  | 16b | Cite studies that might appear to meet the inclusion criteria, but which were excluded, and explain why they were excluded. | Pg. 11-12, Supplement |
| Study characteristics | 17 | Cite each included study and present its characteristics. | Supplement |
| Risk of bias in studies | 18 | Present assessments of risk of bias for each included study. | Pg. 11, Supplement |
| Results of individual studies | 19 | For all outcomes, present, for each study: (a) summary statistics for each group (where appropriate) and (b) an effect estimate and its precision (e.g. confidence/credible interval), ideally using structured tables or plots. | Pg. 11-15, 28-37, Supplement |
| Results of syntheses | 20a | For each synthesis, briefly summarise the characteristics and risk of bias among contributing studies. | Pg. 11-15, 28-37, Supplement |
|  | 20b | Present results of all statistical syntheses conducted. If meta-analysis was done, present for each the summary estimate and its precision (e.g. confidence/credible interval) and measures of statistical heterogeneity. If comparing groups, describe the direction of the effect. | Pg. 11-15, 28-37, Supplement |
|  | 20c | Present results of all investigations of possible causes of heterogeneity among study results. | Pg. 11-15, 28-37, Supplement |
|  | 20d | Present results of all sensitivity analyses conducted to assess the robustness of the synthesized results. | Pg. 11-15, 28-37, Supplement |
| Reporting biases | 21 | Present assessments of risk of bias due to missing results (arising from reporting biases) for each synthesis assessed. | Pg. 11-15, 28-37, Supplement |
| Certainty of evidence | 22 | Present assessments of certainty (or confidence) in the body of evidence for each outcome assessed. | na |
| **DISCUSSION** | | |  |
| Discussion | 23a | Provide a general interpretation of the results in the context of other evidence. | Pg. 15-19 |
|  | 23b | Discuss any limitations of the evidence included in the review. | Pg. 19-20 |
|  | 23c | Discuss any limitations of the review processes used. | Pg. 19-20 |
|  | 23d | Discuss implications of the results for practice, policy, and future research. | Pg. 19-21 |
| **OTHER INFORMATION** | | |  |
| Registration and protocol | 24a | Provide registration information for the review, including register name and registration number, or state that the review was not registered. | Pg. 2, 6 |
|  | 24b | Indicate where the review protocol can be accessed, or state that a protocol was not prepared. | Pg. 2, 6 |
|  | 24c | Describe and explain any amendments to information provided at registration or in the protocol. | Pg. 2, 6, Supplement |
| Support | 25 | Describe sources of financial or non-financial support for the review, and the role of the funders or sponsors in the review. | Pg. 21 |
| Competing interests | 26 | Declare any competing interests of review authors. | Pg. 1, 21 |
| Availability of data, code and other materials | 27 | Report which of the following are publicly available and where they can be found: template data collection forms; data extracted from included studies; data used for all analyses; analytic code; any other materials used in the review. | Pg. 21 |

| **Supplementary Table 2. PRISMA-S Checklist** | | | |
| --- | --- | --- | --- |
| **Section/topic** | **#** | **Checklist item** | **Location(s) Reported** |
| **INFORMATION SOURCES AND METHODS** | | | |
| Database name | 1 | Name each individual database searched, stating the platform for each. | Supplement |
| Multi-database searching | 2 | If databases were searched simultaneously on a single platform, state the name of the platform, listing all of the databases searched. | Supplement |
| Study registries | 3 | List any study registries searched. | Na |
| Online resources and browsing | 4 | Describe any online or print source purposefully searched or browsed (e.g., tables of contents, print conference proceedings, web sites), and how this was done. | Na |
| Citation searching | 5 | Indicate whether cited references or citing references were examined, and describe any methods used for locating cited/citing references (e.g., browsing reference lists, using a citation index, setting up email alerts for references citing included studies). | Pg. 7 |
| Contacts | 6 | Indicate whether additional studies or data were sought by contacting authors, experts, manufacturers, or others. | Pg. 7-8 |
| Other methods | 7 | Describe any additional information sources or search methods used. | Na |
| **SEARCH STRATEGIES** | | | |
| Full search strategies | 8 | Include the search strategies for each database and information source, copied and pasted exactly as run. | Supplement |
| Limits and restrictions | 9 | Specify that no limits were used, or describe any limits or restrictions applied to a search (e.g., date or time period, language, study design) and provide justification for their use. | Supplement |
| Search filters | 10 | Indicate whether published search filters were used (as originally designed or modified), and if so, cite the filter(s) used. | Supplement |
| Prior work | 11 | Indicate when search strategies from other literature reviews were adapted or reused for a substantive part or all of the search, citing the previous review(s). | Pg. 7-8 |
| Updates | 12 | Report the methods used to update the search(es) (e.g., rerunning searches, email alerts). | Pg. 7-8, Supplement |
| Dates of searches | 13 | For each search strategy, provide the date when the last search occurred. | Pg. 7-8, Supplement |
| **PEER REVIEW** | | | |
| Peer review | 14 | Describe any search peer review process. | Pg. 7-8 |
| **MANAGING RECORDS** | | | |
| Total Records | 15 | Document the total number of records identified from each database and other information sources. | Supplement |
| Deduplication | 16 | Describe the processes and any software used to deduplicate records from multiple database searches and other information sources. | Pg. 7-8 |
|  |  |  |  |

**Search Strategy**

- **Search dates: up until 19/01/22**
- **Limits: none**

**PubMed (MEDLINE)**

**Search terms**

(cognitive training [tiab] OR attention training [tiab] OR working memory training [tiab] OR cognitive remediation [tiab] OR executive function training [tiab] OR inhibition training [tiab] or inhibitory training [tiab] OR time training [tiab] or timing training [tiab] or digital intervention [tiab] or digital interventions [tiab] OR video game [tiab] or video games [tiab] OR gamif* [tiab] OR digital health intervention [tiab] or digital health interventions [tiab] OR e-health [tiab] OR m-health [tiab]) AND (ADHD [tiab] OR attention-deficit/hyperactivity disorder [tiab] OR attention-deficit [tiab] OR attention deficit [tiab] OR hyperkinetic syndrome [tiab] or hyperkinetic disorder [tiab]) (random* [tiab] or crossover trial [tiab] OR controlled trial*[tiab])

**OVID databases (PsycInfo, Medline, Embase+Embase Classic)**

(cognitive training OR attention training OR working memory training OR cognitive remediation OR executive function training OR inhibition training or inhibitory training OR time training or timing training or digital intervention or digital interventions OR video game or video games OR gamif* OR digital health intervention or digital health interventions OR e-health OR m-health) AND (ADHD OR attention-deficit hyperactivity disorder OR attention-deficit OR attention deficit OR hyperkinetic syndrome or hyperkinetic disorder) AND (random* or crossover trial OR controlled trial*)

**WEB OF KNOWLEDGE**

**(Web of science (science citation index expanded), Biological abstracts, Biosis, Food science and technology abstracts)**

cognitive training OR attention training OR working memory training OR cognitive remediation OR executive function training OR inhibition training or inhibitory training OR time training or timing training or digital intervention or digital interventions OR video game or video games OR gamif* OR digital health intervention or digital health interventions OR e-health OR m-health

ADHD OR attention-deficit hyperactivity disorder OR attention-deficit OR attention deficit OR hyperkinetic syndrome or hyperkinetic disorder

random* or crossover trial OR controlled trial*


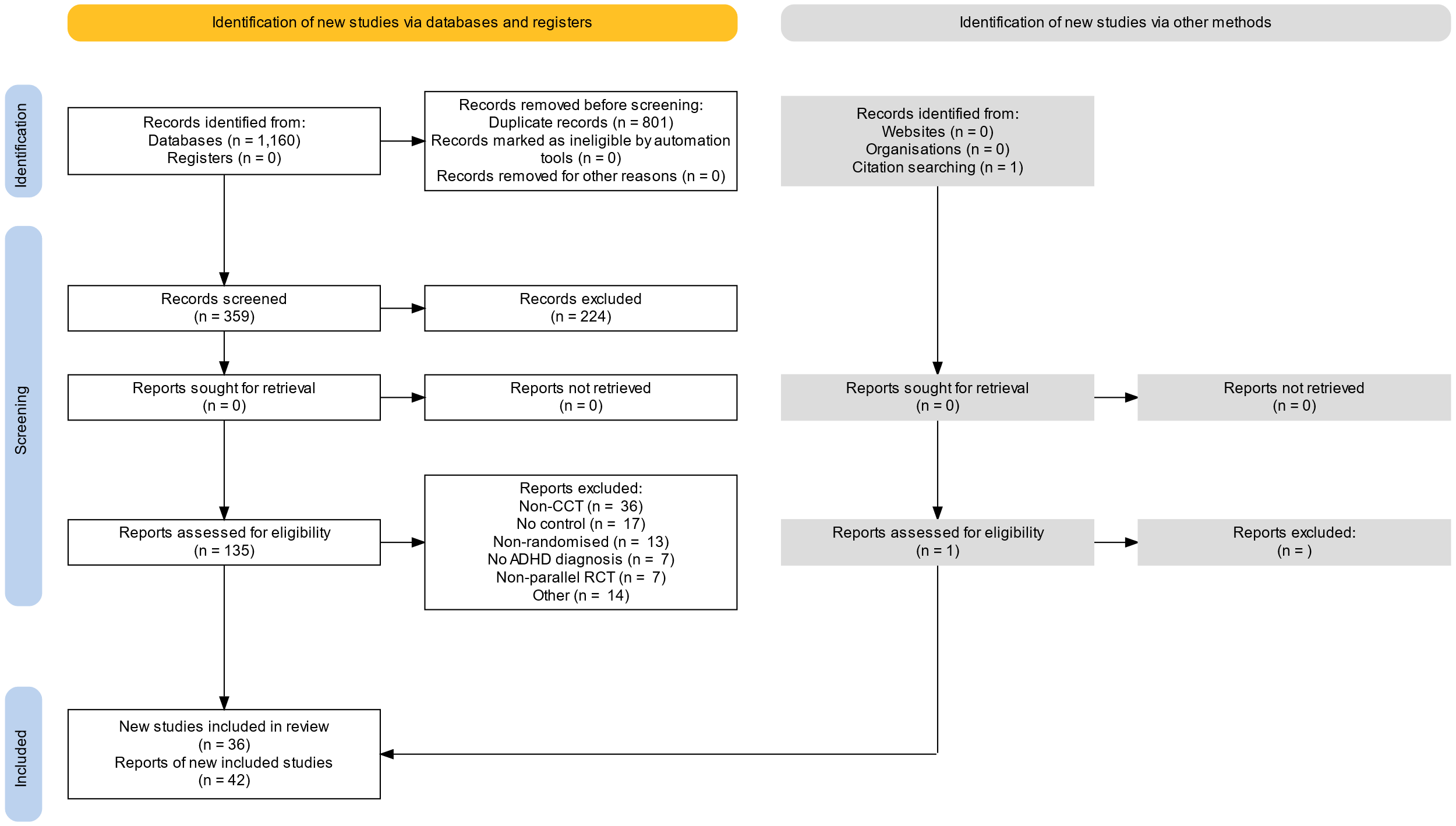


*Supplementary Figure 1. Preferred Reporting Items in Systematic Reviews and Meta-Analyses (PRISMA) 2020 flowchart of study selection (until 19/01/22). For more detailed reasons for exclusion, please Supplement 1 Table 1. Eligible reports based on the same RCT were Rivard reported follow-up data from Dentz et al. (2020a); Hovick et al. (2013) and Egeland et al. (2013); Jaquerod et al. (2020) and Dotare et al. (2020); Hasslinger et al. (2021) and Hasslinger et al. (2022) ; Liu et al. (2017) combined data from Liu et al. (2016), Mawjee et al. (2014), Mawjee et al. (2015); and Woltering et al. (2019) combined data from Mawjee et al. (2014) and Mawjee et al. (2015).*

| Supplementary Table 3. Characteristics of eligible studies | | | | | | | | | | | |  |
| --- | --- | --- | --- | --- | --- | --- | --- | --- | --- | --- | --- | --- |
| **Author** | **Age**  **(yrs; M±SD)^1^** | **N^1^** | **Control** | **CCT** | **CCT Protocol** | **M-Prox** | **P-Blind** | **Clinical Outcomes** | **Cognitive Outcomes** | | **Academic Outcomes** | |
| ***Children and/or adolescents*** | |  |  |  |  |  |  |  |  | |  | |
| Bigorra et al. (2016)(13) | 8.92±1.72 | 65 | NA-CCT (CogMed: MegaMemo) | WMT  (CogMed: RoboMemo) | 24 sessions at home  (5 per week, 30-35-mins each) | Parent | Teacher | CBCL; CRS; BRIEF; SDQ; TRF | CogMed Improvement Index; CPT; Digit Span; Letter-Number Sequencing; Spatial Span; TMT-Part B; Tower of London; WCST | | Canals Reading Comprehension | |
| Bikic et al (2017)(14) | 15.6±.99 | 18 | AC  (Tetris) | MPT (HappyNeuron) | 35 sessions at home  (6 per week, 30-mins each) | Parent | n/a | ADHD-RS^6^; APQ | CANTAB (Rapid Visual Information Processing; Match to Sample Visual Search; Delayed Matching to Sample; Spatial Span; Spatial WM; Stockings of Cambridge; Intra-extra dimensional set shift) | | n/t | |
| Bikic et al (2018)(15) | 9.96±1.75 | 70 | TAU | MPT  C8 Sciences | 48 sessions at home (6 per week, 40-mins each) | Parent | n/a | ADHD-RS; BRIEF; WFI-RS | CANTAB (Attention switching task; Rapid Visual Information Processing; Spatial Working Memory; Stockings of Cambridge; Intra-extra dimensional set shift; Stop signal task; 5-choice reaction time) | | n/t | |
| Bioulac et al. (2020)(16) | 7-11 | 40 | Psychotherapy | AT  (Integrated Media Systems) | 12 sessions at clinic/lab (2 per week, 30-mins each) | Parent | n/a | ADHD-RS | Virtual Classroom CPT; Standard CPT | | n/t | |
| Chacko et al. (2014)(17) | 8.4±1.35 | 81 | NA-CCT | WMT  (CogMed: RoboMemo) | 25 sessions at home (5 per week, 30-45-mins each) | Parent | Teacher | DBD-RS; Actigraph | AWMA (Dot Matrix, Spatial Recall, Digit Recall, Listening Recall); CPT; CogMed Improvement Index | | WRAT (Word Reading, Sentence Completion, Math Computation, Spelling; | |
| Dentz et al. (2020a)(18) | 10.02±1.63 | 52 | NA-CCT | WMT  (CogMed) | 25 sessions at home (5 per week, 30-45-mins each) | Parent | Parent | Conners 3AI; BRIEF | Digit Span; Letter Number Sequencing; Spatial Span; Raven's Matrices; CogMed Improvement Index | | WIAT-II (Reading Comprehension, Mathematical Reasoning) | |
| Dovis et al. (2015)(19) | 10.6±1.4 | 61 | NA-CCT | MPT  (Braingame Brian) | 25 sessions at home (5 per week, 30-mins each) | Parent | Teacher | BRIEF^6^; DBD-RS; PedsQL; SPSRQ ; HSQ | Stop Task; Stroop Task; Corsi Block Tapping Task; Digit Span; TMT; Raven Matrices | | n/t | |
| de Oliveira Rosa et al. (2018)(20) | 10.64±1.80 | 50 | AC  (school work) | MPT  (C8 Sciences) | 48 sessions at home/lab (4 per week, 30-mins each) | Parent | Teacher | SNAP-IV; CGI (I), CGAS (I); Internet Addiction Test (n/r) | NIH Toolbox (Flanker, GNG, WM) | | n/t | |
| Egeland et al. (2013)(21) | 10.5±.75 | 75 | TAU | WMT  (CogMed) | 21-25 sessions at school (n/r, 30-45-mins) | Teacher | n/a | ADHD-RS; SDQ; BRIEF | D-KEFS (Color-Word [Stroop Task], TMT; CPT; Children’s Auditory Verbal Learning Test-2; Benton Visual Retention Test | | Key Math (Mental Computation, Problem-Solving); LOGOS (Reading Fluency, Word Decoding) | |
| Gray et al. (2012)(10) | 14.3±1.2 | 60 | AC  (Math Training) | WMT  (CogMed) | 20-25 sessions at school (4-5 per week, 45-mins each) | Teacher | n/a | SWAN; IOWA Conners | CANTAB (Spatial WM; Spatial Span); D2 Test of Attention; Digit Span; CogMed Improvemend Index | | WM-RS; WRAT-4 (Word Reading, Sentence Comprehension, Spelling, Mathematics) | |
| Green et al. (2012)(22) | 9.75±2.2 | 30 | NA-CCT | WMT  (CogMed) | 25 sessions at home (n/r, 25-45-mins each) | Parent | Parent | CRS-R | Digit-Span; Letter-Number Sequence; Restricted Academic Situations Task | | n/t | |
| Hasslinger et al. (2021)(23) | 12.41±2.58 | 101 | TAU | WMT  (Minneslek Flex^TM^) | 25 session at clinic (5 per week, 45-mins each) | Parent | n/a | CRS; BRIEF | Reported in Hasslinger et al. (2022) | | n/t | |
| Hasslinger et al. (2022)(24) | 12.41±2.58 | 101 | TAU | WMT  (Minneslek Flex^TM^) | 25 session at clinic (5 per week, 45-mins each) | n/a | n/a | Reported in Hasslinger et al. (2021) | Block Tapping Task; CPT; Digit Span Task; Letter-Number Sequencing Task; Spatial WM Task; Time Anticipation Task; Tapping Task | | n/t | |
| Hovik et al. (2013)(25) | 10.5±.75 | 75 | TAU | WMT  (CogMed) | 25 sessions at school (n/r, 30-45-mins) | Teacher | n/a | n/r | Digit Span; Letter-Number Sequencing Task; Sentence Span Task; LIPS-R (Remembering Game, Backwards Game; CogMed Improvement Index | | n/r | |
| Johnstone et al. (2010)(3) | 10.7±1.4 | 20 | NA-CCT | MPT  (In-house GNG & VS-WM) | 25 sessions at home (5 per weekly, 30-mins each) | n/a | n/a | ADHD symptom questionnaire; CBCL; CRS^6^ | GNG Task | | n/t | |
| Johnstone et al. (2012)(26) | 10.0±2.2 | 42 | WLC | MPT  (In-house GNG & VS-WM) | 25 sessions at home (n/r, 15-20-mins each) | n/a | n/a | ADHD symptom questionnaire; CRS^6^ | Counting Span; Digit Span; Flanker Task; GNG Task; Oddball Task; Trained GNG and WM Tasks | | n/t | |
| Jones et al. (2020)(27) | 10.14±2.04 | 80 | AC  (Adaptive knowledge task) | WMT  (In-house n-back) | 20 sessions at home/lab/library/ public (n/r, 15-mins each) | n/a | n/a | BRIEF^4^; CBCL^4^; CRS^4^ | CPT; Digit Span; Following Directions; Raven Matrices; TONI-Matrix Reasoning; 2-back | | WJ-II (Passage Comprehension, Math Applied Problem Solving, Reading Fluency) | |
| Klingberg et al. (2005)(28) | 9.9±1.3 | 53 | NA-CCT | WMT  (CogMed) | 25 sessions at home/school  (n/r, 40-mins) | Parent | Teacher | CRS _;_ DSM-IV Symptom Checklist | Digit-Span; Span-Board; Stroop Task; Raven Matrices | | n/t | |
| Kollins et al (2020)(29) | 8-12 | 348 | AC  (adaptive word game) | MPT  (AKL-T01) | 100 sessions at home (n/r, 5-mins each) | Parent | n/a | ADHD-RS^6^; BRIEF ; Impairment Rating Scale | TOVA | | n/t | |
| Medina et al. (2021)(30) | 8-11 | 40 | AC | MPT (KAD_SCL_01) | 36 sessions at home (3 per week, 15-mins each) | Parent | Parent | CRS ; BRIEF | CPT; Auditory Attention Test; Cognitive Flexibility; Digit Span; Semantic Fluency; Verbal Fluency; Stroop Task; Corsi Block Tapping; Card Classification; Symbol Search Test; Digit Symbol Substitution | | n/t | |
| Meyer et al. (2020)(31) | 10.33±1.33 | 40 | NAC | IT (NeuroScouting) | 20 sessions at home (5 per week, 15-mins each) | Parent | Teacher | CRS ; SNAP-IV | Trained Stop Task | | n/t | |
| Rabiner et al. (2010)(4)^2^ | 5-6 | 50 | WLC | AT  (Braintrain) | 28 sessions at school (2 per week, 60-mins each) | Teacher | n/a | CRS | n/t | | APRS(T); DIBELS Fluency; WJ-III^7^ (Letter-word identification, Word Attack, Math Calculation, Math Fluency, Reading Fluency, Passage Comprehension, Applied Problems) | |
| Rivard et al. (2020)(32) | 7-13 | 61 | NA-CCT | WMT  (CogMed) | 25 sessions at home (5 per week, 30-45-mins each) | Parent | Parent | Conners 3AI; BRIEF | Digit Span; Letter Number Sequencing; Spatial Span; Raven's Matrices | | WIAT-II (Reading Comprehension, Mathematical Reasoning) | |
| Sandberg & McAuley (2021)(33) | 11.59±2.19 | 40 | WLC | WMT  (CogMed) | 30 sessions at hospital (3 per week, 35-mins each) | Parent | n/a | CBCL ; TRF | AWMA (Spatial Recall, Listening Recall); TOVA | | n/t | |
| Shalev et al. (2007)(34) | 6-13 | 36 | AC  (computer games, paper-pencil tasks) | AT  (in house attention tasks) | 16 sessions at lab (2 per week, 60-mins each) | Parent | Parent | ADHD-RS | n/t | | In-house passage copying, math exercises, reading comprehension measures | |
| Simone et al. (2018)(35) | 11.19±2.49 | 20 | AC  (reading exercises) | AT (Attention Processing Training) | 24 sessions at home (2 per week, 60-mins each) | n/a | n/a | n/t | Rao Brief Repeatable Battery (Selective Reminding; Spatial Recall; Symbol Digit Modalities); Semantic Fluency; Tower of London | | n/t | |
| Steiner et al. (2011)(36) | 12.4±.9 | 28 | WLC | MPT  (Braintrain) | 32 sessions at school (2 per week, 30-mins each) | Parent | Teacher | BASC; BRIEF; CRS | CPT^5^ | | n/t | |
| Steiner et al. (2014)(37) | 8.65±1.05 | 70 | WLC | MPT  (Braintrain) | 40 sessions at school (2 per week, 45-mins each) | Parent | Observer | BRIEF ; BOSS; CRS; SKAMP | n/t | | n/t | |
| Tucha et al., (2011)(38) | 11±2 | 32 | AC  (perceptual training) | AT  (AixTent) | 8 sessions at lab (2 per week, 45-mins each) | n/a | n/a | n/t | TAP Battery (Tonic Alertness; Phase Alterness; Vigilance; Selective Attention; Divided Attention; Attention Flexibility) | | n/t | |
| van Dongen-Boomsma et al. (2014)(39) | 6.55±0.65 | 51 | NA-CCT | WMT  (CogMed) | 25 sessions at home (5 per week, 15-mins each) | Investigator | Teacher | ADHD-RS; BRIEF ; CGI-I; CGAS | Digit Span; Knox Cubes; Shortened Raven Coloured Progressive Matrices; SA-DOTS-02K; Shape School; Sentences WPPSI-RN | | n/t | |
| van der Oord et al. (2014)(6) | 9.79±1.04 | 43 | WLC | MPT  (Braingame Brian) | 25 sessions at home (n/r, 40-mins per session) | Parent | Teacher | BRIEF ; DBD-RS | n/t | | n/t | |
| ***Adults*** |  |  |  |  |  |  |  |  |  | |  | |
| Dentz et al. (2020b)(40) | 38.91±12.76 | 55 | NA-CCT | WMT  (CogMed) | 25 sessions at home (5 per week, 30-45-mins each) | Self | Self | CAARS; Brown Attention Deficit Disorder | Digit Span, Letter-Number Sequencing; Matrix Reasoning; Corsi Block-Tapping | | n/t | |
| Dotare et al. (2020)(41) | 18-30 | 76 | NA-CCT | WMT  (Dual N-Back) | >18 sessions at home (n/r, 13-mins each) | n/a | n/a | n/t | Attention Network Task | | n/t | |
| Jaquerod et al. (2020)(42) | 22.2±0.61 | 65 | NA-CCT | WMT  (Dual N-Back) | >18 sessions at home (n/r, 13-mins each) | n/a | n/a | n/t | Probability Gambling Task; Digit Span; Corsi Block-Tapping; Trained Dual n-back | | n/t | |
|  |  |  |  |  |  |  |  |  |  | |  | |
| Liu et al. (2016)(43)^2,3^ | 18-35 | 98 | WLC | WMT  (CogMed: RM Version) | 25 sessions at home (5 per week, 45-mins each) | n/a | n/a | n/r | Delayed match-to-sample-task | |  | |
| Liu et al. (2017)(44)^2,3^ | 18-35 | 92 | WLC | WMT  (CogMed: RM Version) | 25 sessions at home (5 per week, 45-mins each) | n/a | n/a | n/r | GNG Task | |  | |
| Mawjee (45,46)^2,3^ | 23.4±4.6 | 30 | WLC | WMT  (CogMed: RM Version) | 25 sessions at home (5 per week, 45-mins each) | Self | n/a | ASRS; CFQ; BDEFS-SF | Digit Span; Digit Span Sequencing; CANTAB (Spatial Span; Spatial WM; Pattern Recognition Memory); WRAML-2 (Finger Windows); adapted Kahneman's WM Task | | n/t | |
| Mawjee et al. (46)^2,3^ | 23.7±3.4 | 64 | WLC | WMT  (CogMed: RM Version) | 25 sessions at home (5 per week, 45-mins each) | Self | n/a | ASRS; CFQ; BDEFS-SF;  Grit Scale | Digit Span; Digit Span Sequencing; CANTAB (Spatial Span, Spatial WM, Pattern Recognition Memory Task); WRAML-2 (Finger Windows); A Quick Test of Cognitive Speed | | WJ-III Math Fluency; TOWE-II | |
| Salmi et al. (2020)(47) | Adults | 44 | AC  (Bejewelled-II Game) | WMT  (in house dual n-back) | 15 sessions at lab/home (3 per week, 30-mins each) | Self | n/a | ASRS | CPT; Digit Span; Digit & Visuospatial /Running Sequence Span Task; Trained dual n-back | | n/t | |
| Stern et al. (2016)(48) | 37.2±10.15 | 60 | NA-CCT | MPT (*AttenFocus*) | 48-60 sessions at home (4-5 per week, 20-mins each) | Self | n/a | ASRS; BRIEF_;_ COPM; AAQoL | Integneuro (Memory Recall; Memory Recognition; Digit Span; Span of Visual Memory; CPT; Switching Attention; Choice Reaction Time; Time Estimation; Verbal Interference, Verbal Fluency; Semantic Fluency; Maze; GNG; Emotion Recognition) | |  | |
| Virta et al. (2010)(8) | 21-49 | 46 | WLC | MPT  (In house CCT) | 20 sessions at lab (2 per week, 60-mins each) | n/a | n/a | ASRS; BADDS; BDI-II; CGI; SCL-90; Q-Les-Q | CCT Task (Circle-letter sequencing; CPT plus mental arithmetic; Digit arrangement; Digit letter search); CNS Vital Signs Battery (Verbal & Visual Memory; Finger Tapping; Symbol Digit Coding; Stroop Task, Shifting Attention; CPT). | | n/t | |
| Woltering et al. (2019)(49)^2,3^ | 18-35 | 92 | WLC | WMT  (CogMed: RM Version) | 25 sessions at home (5 per week, 45-mins each) | Self | n/a | ASRS | CANTAB Pattern Recognition; Change Detection Task; Digit Span | | n/t | |
| **^1^**at enrolment; ^2^To improve homogeneity across trials, we excluded CCT arms from the meta-analyses that were inconsistent with the other studies in the meta-analysis. This meant excluding the “software with attention monitoring” arm from Johnston et al. (2012); the “shortened-length treatment” arm from Mawjee et al. (2014, 2015), Liu et al. (2016, 2017), and Woltering et al. (2019); the “computer-assisted instruction” arm from Rabiner et al. (2010); and the neurofeedback arm from Steiner et al. (2011); ^3^Several reports of the same study; ^4^at follow-up only; ^5^treatment arms only; ^6^outcome not included in any analysis. Bikic et al (2017) an ADHD total score that was conflated with ODD symptoms, and subscales were not available after person correspondence. Dovis et al. (2015) did not report GEC. Johnstone et al. (2010; 2012) did not provide data following reasonable requests. In Kollins et al. (2020), Akili Interactive Labs, Inc did not provide data upon reasonable request; ^7^Only Letter-word identification, Word Attack, Calculation, and Math Fluency were administered at all assessment timepoint; AAQoL; AC, active control; ADHD-RS, ADHD-Rating Scale; APRS, Academic Performance Rating Scale; APQ, activity perception questionnaire; AT, attention training; AWMA, Automated Working Memory Assessment; ASRS, Adult ADHD Self-Report Scale; BADDS, Brown Attention-Deficit Disorder Scale; BASC, Behavior Assessment System for Children; BDEFS-SF, Barkley Deficits in Executive Functioning Scale–Short Form; BDI-II, Beck Depression Inventory; BRIEF, Behavior Rating Inventory of Executive Function; BOSS, Behavioral Observation of Students in Schools; CANTAB, Cambridge Neuropsychological Test Automated Battery; CAARS, Conners’ Adult ADHD Rating Scale; CBCL, Child Behaviour Checklist; CCT, computerised cognitive training; CFQ, Cognitive Failures Questionnaire; COPM, Canadian Occupational Performance Measure; CPT, continuous performance task; CGAS, Children's Global Assessment Scale; CGI, clinical global impression; CRS-R, Conners’ Rating Scale-Revised; DBD-RS, Disruptive Behaviour Disorder-Rating Scale; DIBELS, Dynamic Indicators of Basic Early Literacy Skills; D-KEFS, Delis–Kaplan Executive Function System; GNG, Go/No-Go; HSQ, Home Situations Questionnaire; LIPS-R, Leiter International Performance Scale-Revised; MPT, multi-process training; n/a, not available as rated was not blinded; NA-CCT, non-adaptive equivalent of the CCT; n/r, not reported; n/t, not tested; PedsQL, Pediatric Quality of Life Inventory; SCL-90 (S), Symptom Checklist-90; SDQ, strengths and difficulties questionnaire; SKAMP, Swanson, Kotkin, Agler, M-Flynn, and Pelham Rating Scale; SPSRQ, Sensitivity to Punishment and Sensitivity to Reward Questionnaire; SNAP-IV, Swanson, Nolan, and Pelham Rating Scale; SWAN, Strengths & Weaknesses of ADHD and Normal behaviour; TAP Battery, Test of Attentional Performance Battery; TAU, treatment-as-usual; TONI, Test of Nonverbal Intelligence; TOVA, Test of Visual Attention; TOWE-II, Test of Word Reading Efficiency-II; TMT, trail making task; TRF, teacher report form; WCST, Wisconsin Card Sorting Task; WFI-RS, Weiss Functional Impairment Rating Scale; WIAT-II, Wechsler Individual Achievement Test 2nd Edition; WJ-II, Woodcock Johnson Tests 2nd Edition; WM, working memory; WM-RS, working memory-rating scale; WMT, working memory training; WRAT-4, Wide Range Achievement Test 4; WRAML, Wide Range Assessment of Memory and Learning; Q-Les-Q, Quality of Life Enjoyment and Satisfaction Questionnaire; IT, Inhibitory Control Training | | | | | | | | | | | | |
| Supplementary Table 4. Studies excluded from the meta-analysis, with reasons. | | | | | | | | | | | |  |
| **Paper** | | | | | | | **Reasons for exclusion** | | | **As reported in PRISMA 2020 Flowchart** | |  |
| Abikoff H, Gallagher R, Wells KC, Murray DW, Huang L, Petkova E. Remediating Organizational Functioning in Children With ADHD: Immediate and Long-Term Effects From a Randomized Controlled Trial. J Consult Clin Psychol 2013;81:113-28. | | | | | | | Does not meet definition of cognitive training according to meta-analysis protocol | | | Non-CCT | |  |
| Abikoff H, Gittelman R. Hyperactive children treated with stimulants. Is cognitive training a useful adjunct? Arch Gen Psychiatry 1985;42:953-961. | | | | | | | Does not meet definition of cognitive training according to meta-analysis protocol | | | Non-CCT | |  |
| Ackermann, S., Halfon, O., Fornari, E., Urben, S., & Bader, M. (2018). Cognitive Working Memory Training (CWMT) in adolescents suffering from Attention-Deficit/Hyperactivity Disorder (ADHD): A controlled trial taking into account concomitant medication effects. Psychiatry Res, 269, 79-85. doi:10.1016/j.psychres.2018.07.036 | | | | | | | No control with ADHD | | | No control | |  |
| Azami, S., Moghadas, A., Sohrabi-Esmrood, F., Nazifi, M., Mirmohamad, M., Hemmati, F., . . . Lakes, K. (2016). A pilot randomized controlled trial comparing computer-assisted cognitive rehabilitation, stimulant medication, and an active control in the treatment of ADHD. Child Adolesc Ment Health, 21(4), 217-224. doi:10.1111/camh.12157 | | | | | | | Active control had adaptive component | | | No control | |  |
| Barimani, S., Asadi, J., & Khajevand, A. (2018). A Comparison between the Effectiveness of Game Therapy and Emotional Intelligence Training on Social Compatibility and Communicative Skills of Exceptional Primary School Hyperactive and Deaf Children. International Journal of Pediatrics-Mashhad, 6(5), 7653-7666. doi:10.22038/ijp.2017.27514.2374 | | | | | | | No definitive diagnosis of ADHD among inclusion criteria | | | No ADHD diagnosis | |  |
| Beck, S. J., Hanson, C. A., Puffenberger, S. S., Benninger, K. L., & Benninger, W. B. (2010). A controlled trial of working memory training for children and adolescents with ADHD. J Clin Child Adolesc Psychol, 39(6), 825-836. doi:10.1080/15374416.2010.517162 | | | | | | | Non-randomised | | | Non-randomised | |  |
| Benzing, V., & Schmidt, M. (2017). Cognitively and physically demanding exergaming to improve executive functions of children with attention deficit hyperactivity disorder: a randomised clinical trial. Bmc Pediatrics, 17, 8. doi:10.1186/s12887-016-0757-9 | | | | | | | Protocol | | | Other | |  |
| Benzing, V., & Schmidt, M. (2019). The effect of exergaming on executive functions in children with ADHD: A randomized clinical trial. Scand J Med Sci Sports, 29(8), 1243-1253. doi:10.1111/sms.13446 | | | | | | | Does not meet definition of cognitive training according to meta-analysis protocol | | | Non-CCT | |  |
| Bul, K. C. M., Doove, L. L., Franken, I. H. A., Van Der Oord, S., Kato, P. M., & Maras, A. (2018). A serious game for children with Attention Deficit Hyperactivity Disorder: Who benefits the most? Plos One, 13(3). doi:http://dx.doi.org/10.1371/journal.pone.0193681 | | | | | | | Crossover trial | | | Non-parallel RCT | |  |
| Bul, K. C. M., Franken, I. H. A., Van der Oord, S., Kato, P. M., Danckaerts, M., Vreeke, L. J., . . . Maras, A. (2015). Development and user satisfaction of "Plan-It Commander," a serious game for children with ADHD. [References]. Games for Health, 4(6), 502-512. doi:http://dx.doi.org/10.1089/g4h.2015.0021 | | | | | | | Non-randomised | | | Non-randomised | |  |
| Bul, K.C., et al., Behavioral Outcome Effects of Serious Gaming as an Adjunct to Treatment for Children With Attention-Deficit/Hyperactivity Disorder: A Randomized Controlled Trial. Journal of Medical Internet Research, 2016. 18(2): p. 16. | | | | | | | Crossover trial | | | Non-parallel RCT | |  |
| Capodieci, A., Gola, M. L., Cornoldi, C., & Re, A. M. (2018). Effects of a working memory training program in preschoolers with symptoms of attention-deficit/hyperactivity disorder. J Clin Exp Neuropsychol, 40(1), 17-29. doi:10.1080/13803395.2017.1307946 | | | | | | | Does not meet definition of cognitive training according to meta-analysis protocol | | | Non-CCT | |  |
| Cho, B.-H., Ku, J., Jang, D., Lee, J., Oh, M., Kim, H., . . . Kim, S. (2002). Clinical test for Attention Enhancement System. Studies in Health Technology & Informatics, 85, 89-95. Retrieved from https://proxy.library.nyu.edu/login?url=http://ovidsp.ovid.com/ovidweb.cgi?T=JS&CSC=Y&NEWS=N&PAGE=fulltext&D=med4&AN=15458066 | | | | | | | No participant inclusion criteria | | | Other | |  |
| Davis, N. O., Bower, J., & Kollins, S. H. (2018). Proof-of-concept study of an at-home, engaging, digital intervention for pediatric ADHD. PLoS One, 13(1), e0189749. doi:10.1371/journal.pone.0189749 | | | | | | | Non-randomised | | | Non-randomised | |  |
| Davis, N., et al. (2020). "5.27 Stars-Adjunct: Akl-T01, a Home-Based Digital Intervention as an Adjunct to Stimulant Medication for Pediatric Adhd: Academic Performance and Relation to Objective Measures of Attention." Journal of the American Academy of Child and Adolescent Psychiatry 59(10 Supplement): S157-S158. | | | | | | | Poster | | | Other | |  |
| Douglas VI, Parry P, Marton P, Garson C. Assessment of a cognitive training program for hyperactive children. J Abnorm Child Psychol 1976;4:389-410. | | | | | | | Non-randomised | | | Non-randomised | |  |
| Dovis, S., Maric, M., Prins, P. J. M., & Van der Oord, S. (2019). Does executive function capacity moderate the outcome of executive function training in children with ADHD? Atten Defic Hyperact Disord, 11(4), 445-460. doi:10.1007/s12402-019-00308-5 | | | | | | | Re-analysis of Dovis et al. (2015) | | | Other | |  |
| Estrada-Plana, V., Esquerda, M., Mangues, R., March-Llanes, J., & Moya-Higueras, J. (2019). A Pilot Study of the Efficacy of a Cognitive Training Based on Board Games in Children with Attention-Deficit/Hyperactivity Disorder: A Randomized Controlled Trial. Games Health J, 8(4), 265-274. doi:10.1089/g4h.2018.0051 | | | | | | | Does not meet definition of cognitive training according to meta-analysis protocol | | | Non-CCT | |  |
| Gaynor A, Whitman J, Bessette K, Stevens M. Effects of intensive working memory treatment on brain activity in adolescent combined-subtype ADHD. Biological Psychiatry. Conference: 68th Annual Scientific Convention and Meeting of the Society of Biological Psychiatry, SOBP. 2013;73(9 SUPPL. 1). | | | | | | | Not controlled | | | No control | |  |
| Gibson, B. S., Gondoli, D. M., Johnson, A. C., Steeger, C. M., Dobrzenski, B. A., & Morrissey, R. A. (2011). Component analysis of verbal versus spatial working memory training in adolescents with ADHD: a randomized, controlled trial. Child Neuropsychol, 17(6), 546-563. doi:10.1080/09297049.2010.551186 | | | | | | | Both arms cognitive training | | | No control | |  |
| Gibson, B. S., Gondoli, D. M., Kronenberger, W. G., Johnson, A. C., Steeger, C. M., & Morrissey, R. A. (2013). Exploration of an adaptive training regimen that can target the secondary memory component of working memory capacity. Memory & Cognition, 41(5), 726-737. doi:10.3758/s13421-013-0295-8 | | | | | | | No ADHD diagnosis | | | No ADHD diagnosis | |  |
| Goodwin, A., et al. (2021). "INTERSTAARS: Attention training for infants with elevated likelihood of developing ADHD: A proof-of-concept randomised controlled trial." Transl Psychiatry 11(1): 644. | | | | | | | No definitive diagnosis of ADHD among inclusion criteria | | | No ADHD diagnosis | |  |
| Gropper, R. J., Gotlieb, H., Kronitz, R., & Tannock, R. (2014). Working memory training in college students with ADHD or LD. J Atten Disord, 18(4), 331-345. doi:10.1177/1087054713516490 | | | | | | | Mixed sample of ADHD and LD | | | Other | |  |
| Hajiheidary, F., et al. (2020). Comparison of the effectiveness of cognitive rehabilitation programs of Brain training and Cogni Plus on cognitive skills of students with Attention Deficit Hyperactivity Disorder. 2nd International Serious Games Symposium (ISGS), Tehran, IRAN, Ieee. | | | | | | | Both arms cognitive training | | | No control | |  |
| Halperin, J. M., et al. (2020). "Training Executive, Attention, and Motor Skills (TEAMS): a Preliminary Randomized Clinical Trial of Preschool Youth with ADHD." Journal of Abnormal Child Psychology 48(3): 375-389. | | | | | | | Does not meet definition of cognitive training according to meta-analysis protocol | | | Non-CCT | |  |
| Halperin, J. M., Marks, D. J., Chacko, A., Bedard, A. C., O'Neill, S., Curchack-Lichtin, J., . . . Berwid, O. G. (2020). Training Executive, Attention, and Motor Skills (TEAMS): a Preliminary Randomized Clinical Trial of Preschool Youth with ADHD. Journal of Abnormal Child Psychology, 48(3), 375-389. doi:10.1007/s10802-019-00610-w | | | | | | | Does not meet definition of cognitive training according to meta-analysis protocol | | | Non-CCT | |  |
| Hannesdottir, D. K., Ingvarsdottir, E., & Bjornsson, A. (2017). The OutSMARTers Program for Children With ADHD: A Pilot Study on the Effects of Social Skills, Self-Regulation, and Executive Function Training. Journal of Attention Disorders, 21(4), 353-364. doi:10.1177/1087054713520617 | | | | | | | Does not meet definition of cognitive training according to meta-analysis protocol | | | Non-CCT | |  |
| Holmes J, Gathercole S, Place M, Dunning DL, Hilton KA, Elliott JG. Working memory deficits can be overcome: impacts of training and medicaton on working memory in children with ADHD. Appl Cogn Psychol 2009; DOI: 10.1002/acp. | | | | | | | Not controlled | | | No control | |  |
| Hosainzadeh Maleki, Z., Mashhadi, A., Soltanifar, A., Moharreri, F., & Ghanaei Ghamanabad, A. (2014). Barkley's Parent Training Program, Working Memory Training and their Combination for Children with ADHD: Attention Deficit Hyperactivity Disorder. Iran J Psychiatry, 9(2), 47-54. Retrieved from https://www.ncbi.nlm.nih.gov/pmc/articles/PMC4300465/pdf/IJPS-9-47.pdf | | | | | | | Not controlled | | | No control | |  |
| Jalilvand, M. and R. Souri (2021). "Effectiveness of motor activity-based executive function training on working memory and sustained attention of children with attention-deficit/hyperactivity disorder. [Farsi (Iranian)]. [References]." Advances in Cognitive Science 22(4): 84-93. | | | | | | | Does not meet definition of cognitive training according to meta-analysis protocol | | | Non-CCT | |  |
| Joekar, S., Amiri, S., Joekar, S., Birashk, B., & Aghebati, A. (2017). Effectiveness of a Visual Attention Training Program on the Reduction of ADHD Symptoms in Preschool Children at Risk for ADHD in Isfahan: A Pilot Study. Iranian Journal of Psychiatry and Behavioral Sciences, 11(4), 6. doi:10.5812/ijpbs.7862 | | | | | | | No ADHD diagnosis; cognitive training not computerised | | | Non-CCT | |  |
| Johnstone, S. J., Roodenrys, S. J., Johnson, K., Bonfield, R., & Bennett, S. J. (2017). Game-based combined cognitive and neurofeedback training using Focus Pocus reduces symptom severity in children with diagnosed AD/HD and subclinical AD/HD. Int J Psychophysiol, 116, 32-44. doi:10.1016/j.ijpsycho.2017.02.015 | | | | | | | Does not meet definition of cognitive training according to meta-analysis protocol | | | Non-CCT | |  |
| Jonkman, L. M., Hurks, P. P., & Schleepen, T. M. J. (2016). Effects of memory strategy training on performance and event-related brain potentials of children with ADHD in an episodic memory task. Neuropsychological Rehabilitation, 26(5-6), 910-941. doi:https://dx.doi.org/10.1080/09602011.2015.1070735 | | | | | | | Non-randomised | | | Non-randomised | |  |
| Kajka, N. and A. Kulik (2021). "The Influence of Metacognitive Strategies on the Improvement of Reaction Inhibition Processes in Children with ADHD." International Journal of Environmental Research and Public Health 18(3): 9. | | | | | | | Does not meet definition of cognitive training according to meta-analysis protocol | | | Non-CCT | |  |
| Karatekin C. Improving antisaccade performance in adolescents with attention-deficit/hyperactivity disorder (ADHD). Exp Brain Res 2006;174:324-341. | | | | | | | Non-randomised | | | Non-randomised | |  |
| Kerns, K.A., Eso, K., Thompson, J., 1999. Investigation of a direct intervention for improving attention in young children with ADHD. Developmental Neuropsychology 16, 273–295. | | | | | | | Non-randomised | | | Non-randomised | |  |
| Khalili Kermani, F., Mohammadi, M. R., Yadegari, F., Haresabadi, F., & Sadeghi, S. M. (2016). Working Memory Training in the Form of Structured Games in Children with Attention Deficit Hyperactivity Disorder. Iran J Psychiatry, 11(4), 224-233. Retrieved from https://www.ncbi.nlm.nih.gov/pmc/articles/PMC5206324/pdf/IJPS-11-224.pdf | | | | | | | Does not meet definition of cognitive training according to meta-analysis protocol | | | Non-CCT | |  |
| Klingberg T, Forssberg H, Westerberg H. Training of working memory in children with ADHD. J Clin Exp Neuropsychol 2002;24:781-791. | | | | | | | Non-randomised | | | Non-randomised | |  |
| Kofler, M. J., Wells, E. L., Singh, L. J., Soto, E. F., Irwin, L. N., Groves, N. B., . . . Lonigan, C. J. (2020). A randomized controlled trial of central executive training (CET) versus inhibitory control training (ICT) for ADHD. J Consult Clin Psychol, 88(8), 738-756. doi:10.1037/ccp0000550 | | | | | | | Both arms cognitive training with no clearly defined control group | | | No control | |  |
| Kray, J., Karbach, J., Haenig, S., & Freitag, C. (2011). Can task-switching training enhance executive control functioning in children with attention deficit/-hyperactivity disorder? Front Hum Neurosci, 5, 180. doi:10.3389/fnhum.2011.00180 | | | | | | | Crossover trial | | | Non-parallel RCT | |  |
| Lim CG, Lee TS, Guan C, et al. A Brain-Computer Interface Based Attention Training Program for Treating Attention Deficit Hyperactivity Disorder. PloS One 2012;7:e46692. | | | | | | | Not controlled | | | No control | |  |
| Lloyd A, Brett D, Wesnes K. Coherence training in children with attention-deficit hyperactivity disorder: cognitive functions and behavioral changes. Altern Ther Health Med 2010;16:34-42. | | | | | | | Does not meet definition of cognitive training according to meta-analysis protocol | | | Non-CCT | |  |
| Lotfi, S., et al. (2020). "Effects of computerized cognitive training for children with dyslexia: An ERP study." Journal of Neurolinguistics 55: 10. | | | | | | | No ADHD diagnosis | | | No ADHD diagnosis | |  |
| Margherio, S. M., et al. "Cost-Effectiveness of a Training Intervention for Adolescents with ADHD." Journal of Clinical Child and Adolescent Psychology: 15. | | | | | | | Does not meet definition of cognitive training according to meta-analysis protocol | | | Non-CCT | |  |
| McDermott, A. F., Rose, M., Norris, T., & Gordon, E. (2020). A Novel Feed-Forward Modeling System Leads to Sustained Improvements in Attention and Academic Performance. J Atten Disord, 24(10), 1443-1456. doi:10.1177/1087054715623044 | | | | | | | Neurofeedback | | | Non-CCT | |  |
| Menezes, A., Dias, N. M., Trevisan, B. T., Carreiro, L. R. R., & Seabra, A. G. (2015). Intervention for executive functions in attention deficit and hyperactivity disorder. Arquivos de Neuro-psiquiatria, 73, 227-236. | | | | | | | Does not meet definition of cognitive training according to meta-analysis protocol | | | Non-CCT | |  |
| Mezzacappa E, Buckner JC. Working memory training for children with attention problems or hyperactivity: a school-based pilot study. School Mental Health 2010; 202-208. | | | | | | | Not controlled | | | No control | |  |
| Minder, F., Zuberer, A., Brandeis, D., & Drechsler, R. (2018). Informant-related effects of neurofeedback and cognitive training in children with ADHD including a waiting control phase: a randomized-controlled trial. Eur Child Adolesc Psychiatry, 27(8), 1055-1066. doi:10.1007/s00787-018-1116-1 | | | | | | | Both arms treatment (neurofeedback, CCT); neurofeedback was not described as control | | | No control | |  |
| Mohammadi, M. R., Soleimani, A. A., Farahmand, Z., Keshavarzi, S., & Ahmadi, N. (2014). A comparison of effectiveness of regulation of working memory function and methylphenidate on remediation of attention deficit hyperactivity disorder (ADHD). Iran J Psychiatry, 9(1), 25-30. Retrieved from [https://www.ncbi.nlm.nih.gov/pmc/articles/PMC4277604/pdf/IJPS-9-25.pdf](https://eur03.safelinks.protection.outlook.com/?url=https%3A%2F%2Fwww.ncbi.nlm.nih.gov%2Fpmc%2Farticles%2FPMC4277604%2Fpdf%2FIJPS-9-25.pdf&data=04%7C01%7Csamuel.westwood%40kcl.ac.uk%7C216a7dbd48a9408fdb8f08d8d1f69ed6%7C8370cf1416f34c16b83c724071654356%7C0%7C0%7C637490204452946702%7CUnknown%7CTWFpbGZsb3d8eyJWIjoiMC4wLjAwMDAiLCJQIjoiV2luMzIiLCJBTiI6Ik1haWwiLCJXVCI6Mn0%3D%7C1000&sdata=HmiGkv%2B0qBxTLLS3rrwpMA%2Btt7O1yYTWbNgT46G0CdU%3D&reserved=0) | | | | | | | Unclear if adaptive, authors did not respond to requests to clarify | | | Other | |  |
| Moore SF, Cole SD. Cognitive self-mediation training with hyperkinetic children. Bull Psychon Soc 1978;18-20 | | | | | | | Does not meet definition of cognitive training according to meta-analysis protocol | | | Non-CCT | |  |
| Moore, A. L., Carpenter, D. M., 2nd, Miller, T. M., & Ledbetter, C. (2018). Clinician-delivered cognitive training for children with attention problems: effects on cognition and behavior from the ThinkRx randomized controlled trial. Neuropsychiatr Dis Treat, 14, 1671-1683. doi:10.2147/ndt.S165418 | | | | | | | No ADHD diagnosis | | | No ADHD diagnosis | |  |
| Nejati, V. (2020). Cognitive rehabilitation in children with attention deficit- hyperactivity disorder: Transferability to untrained cognitive domains and behavior. Asian J Psychiatr, 49, 101949. doi:10.1016/j.ajp.2020.101949 | | | | | | | Excluded because the data provided by the author did not answer our request | | | Other | |  |
| Nejati, V. (2021). "Program for attention rehabilitation and strengthening (PARS) improves executive functions in children with attention deficit- hyperactivity disorder (ADHD)." Res Dev Disabil 113: 103937. | | | | | | | Does not meet definition of cognitive training according to meta-analysis protocol | | | Non-CCT | |  |
| O'Connell, R. G., Bellgrove, M. A., Dockree, P. A., & Robertson, I. H. (2006). Cognitive remediation in ADHD: Effects of periodic non-contingent alerts on sustained attention to response. Neuropsychological Rehabilitation, 16(6), 653-665. doi:10.1080/09602010500200250 | | | | | | | Does not meet definition of cognitive training according to meta-analysis protocol | | | Non-CCT | |  |
| de Oliveira Rosa V, Rosa Franco A, Abrahão Salum Júnior G, Moreira-Maia CR, Wagner F, Simioni A, de Fraga Bassotto C, R Moritz G, Schaffer Aguzzoli C, Buchweitz A, Schmitz M, Rubia K, Paim Rohde LA. Effects of computerized cognitive training as add-on treatment to stimulants in ADHD: a pilot fMRI study. Brain Imaging Behav. 2020 Oct;14(5):1933-1944. doi: 10.1007/s11682-019-00137-0. PMID: 31218531. | | | | | | | Did not share data | | | Other | |  |
| Pahlevanian, A., Alirezaloo, N., Naghel, S., Alidadi, F., Nejati, V., & Kianbakht, M. (2017). Neurofeedback Associated with Neurocognitive-Rehabilitation Training on Children with Attention-Deficit/Hyperactivity Disorder (ADHD). International Journal of Mental Health and Addiction, 15(1), 100-109. doi:10.1007/s11469-015-9621-7 | | | | | | | Does not meet definition of cognitive training according to meta-analysis protocol | | | Non-CCT | |  |
| Pan, C. Y., Chu, C. H., Tsai, C. L., Lo, S. Y., Cheng, Y. W., & Liu, Y. J. (2016). A racket-sport intervention improves behavioral and cognitive performance in children with attention-deficit/hyperactivity disorder. Research in developmental disabilities, 57, 1-10. | | | | | | | Does not meet definition of cognitive training according to meta-analysis protocol | | | Non-CCT | |  |
| Papazian O, Alfonso I, Luzondo RJ, Araguez N. Training of executive function in preschool children with combined attention deficit hyperactivity disorder: a prospective, controlled and randomized trial. Rev Neurol 2009;48 Suppl 2:S119-S122. | | | | | | | No usable outcome at Time 1 | | | Other | |  |
| Perra, O., et al. (2020). "Training attention control of very preterm infants: Protocol for a feasibility study of the Attention Control Training (ACT)." Pilot and Feasibility Studies 6(1) (no pagination). | | | | | | | No ADHD diagnosis | | | No ADHD diagnosis | |  |
| Perra, O., et al. (2021). "Very preterm infants engage in an intervention to train their control of attention: results from the feasibility study of the Attention Control Training (ACT) randomised trial." Pilot and Feasibility Studies 7(1) (no pagination). | | | | | | | No ADHD diagnosis | | | No ADHD diagnosis | |  |
| Prins PJ, Dovis S, Ponsioen A, Ten Brink E, Van der Oord S. Does computerized working memory training with game elements enhance motivation and training efficacy in children with ADHD? Cyberpsychology, Behavior, and Social Networking 2011; 14, 115-122. | | | | | | | Both arms working memory training | | | No control | |  |
| Prins, P. J., Dovis, S., Ponsioen, A., ten Brink, E., & van der Oord, S. (2011). Does computerized working memory training with game elements enhance motivation and training efficacy in children with ADHD? Cyberpsychol Behav Soc Netw, 14(3), 115-122. doi:10.1089/cyber.2009.0206 | | | | | | | Both arms working memory training | | | No control | |  |
| Qian, X., Loo, B. R. Y., Castellanos, F. X., Liu, S., Koh, H. L., Poh, X. W. W., . . . Zhou, J. (2018). Brain-computer-interface-based intervention re-normalizes brain functional network topology in children with attention deficit/hyperactivity disorder. Transl Psychiatry Psychiatry, 8(1), 149. doi:https://dx.doi.org/10.1038/s41398-018-0213-8 | | | | | | | Neurofeedback | | | Non-CCT | |  |
| Qian, Y., Chen, M., Shuai, L., Cao, Q. J., Yang, L., & Wang, Y. F. (2017). Effect of an Ecological Executive Skill Training Program for School-aged Children with Attention Deficit Hyperactivity Disorder: A Randomized Controlled Clinical Trial. Chin Med J (Engl), 130(13), 1513-1520. doi:10.4103/0366-6999.208236 | | | | | | | Does not meet definition of cognitive training according to meta-analysis protocol | | | Non-CCT | |  |
| Qian, Y., Fan, Z. L., Gao, B. L., Margaret, S., Cao, Q. J., Li, F., & Yang, L. Efficacy and acceptability of a second dose of ecological executive skills training for children with ADHD: a randomized controlled study and follow-up. European Child & Adolescent Psychiatry, 15. doi:10.1007/s00787-020-01571-y | | | | | | | Does not meet definition of cognitive training according to meta-analysis protocol | | | Non-CCT | |  |
| Rajabi, S., Pakize, A., & Moradi, N. (2020). Effect of combined neurofeedback and game-based cognitive training on the treatment of ADHD: A randomized controlled study. Appl Neuropsychol Child, 9(3), 193-205. doi:10.1080/21622965.2018.1556101 | | | | | | | Does not meet definition of cognitive training according to meta-analysis protocol | | | Non-CCT | |  |
| Rapport MD, Loo S, Isaacs P, Goya S, Denney C, Scanlan S. Methylphenidate and attentional training. Comparative effects on behavior and neurocognitive performance in twin girls with attention-deficit/hyperactivity disorder. Behav Modif 1996;20:428-430. | | | | | | | Case report | | | Non-parallel RCT | |  |
| Re, A. M., Capodieci, A., & Cornoldi, C. (2015). Effect of training focused on executive functions (attention, inhibition, and working memory) in preschoolers exhibiting ADHD symptoms. Front Psychol, 6, 1161. doi:10.3389/fpsyg.2015.01161 | | | | | | | Does not meet definition of cognitive training according to meta-analysis protocol | | | Non-CCT | |  |
| Rodrigo-Yanguas, M., et al. (2021). "A Virtual Reality Game (The Secret Trail of Moon) for Treating Attention-Deficit/Hyperactivity Disorder: Development and Usability Study." JMIR Serious Games 9(3): e26824. | | | | | | | No usuable outcomes | | | Other | |  |
| Rodrigo-Yanguas, M., et al. (2021). "A Virtual Reality Serious Videogame Versus Online Chess Augmentation in Patients with Attention Deficit Hyperactivity Disorder: A Randomized Clinical Trial." Games Health J 10(4): 283-292. | | | | | | | Protocol | | | Other | |  |
| Rosa, V. D., Schmitz, M., Moreira-Maia, C. R., Wagner, F., Londero, I., Bassotto, C. D., . . . Rohde, L. A. P. (2017). Computerized cognitive training in children and adolescents with attention deficit/hyperactivity disorder as add-on treatment to stimulants: feasibility study and protocol description. Trends in Psychiatry and Psychotherapy, 39(2), 65-76. doi:10.1590/2237-6089-2016-0039 | | | | | | | Case series | | | Non-parallel RCT | |  |
| Sadeghi, M., et al. (2020). "Examining the impact of motivation on working memory training in youth with ADHD." Journal of the Canadian Academy of Child and Adolescent Psychiatry 29(1): 4-14. | | | | | | | Subset of only treatment participants reported in Sandberg & McAuley (2020) | | | Other | |  |
| Salomone, S., Fleming, G. R., Shanahan, J. M., Castorina, M., Bramham, J., O'Connell, R. G., & Robertson, I. H. (2015). The effects of a Self-Alert Training (SAT) program in adults with ADHD. Frontiers in Human Neuroscience, 9, 14. doi:10.3389/fnhum.2015.00045 | | | | | | | Does not meet definition of cognitive training according to meta-analysis protocol | | | Non-CCT | |  |
| Semrud-Clikeman M, Nielsen KH, Clinton A, Sylvester L, Parle N, Connor RT. An intervention approach for children with teacher- and parent-identified attentional difficulties. J Learn Disabil 1999;32:581-590. | | | | | | | Non-randomised | | | Non-randomised | |  |
| Shaffer, R. J., Jacokes, L. E., Cassily, J. F., Greenspan, S. I., Tuchman, R. F., & Stemmer, P. J., Jr. (2001). Effect of interactive metronome training on children with ADHD. Am J Occup Ther, 55(2), 155-162. doi:10.5014/ajot.55.2.155 | | | | | | | Does not meet definition of cognitive training according to meta-analysis protocol | | | Non-CCT | |  |
| Shuai, L., Daley, D., Wang, Y. F., Zhang, J. S., Kong, Y. T., Tan, X., & Ji, N. (2017). Executive Function Training for Children with Attention Deficit Hyperactivity Disorder. Chinese Medical Journal, 130(5), 549-558. doi:10.4103/0366-6999.200541 | | | | | | | Does not meet definition of cognitive training according to meta-analysis protocol | | | Non-CCT | |  |
| Shuai, L., et al. (2021). "Executive Function Training for Preschool Children With ADHD: A Randomized Controlled Trial." Journal of Attention Disorders 25(14): 2037-2047. | | | | | | | Does not meet definition of cognitive training according to meta-analysis protocol | | | Non-CCT | |  |
| Smith, S. D., Crowley, M. J., Ferrey, A., Ramsey, K., Wexler, B. E., Leckman, J. F., & Sukhodolsky, D. G. (2019). Effects of Integrated Brain, Body, and Social (IBBS) intervention on ERP measures of attentional control in children with ADHD. Psychiatry Res, 278, 248-257. doi:10.1016/j.psychres.2019.06.021 | | | | | | | Does not meet definition of cognitive training according to meta-analysis protocol | | | Non-CCT | |  |
| Steeger, C. M., Gondoli, D. M., Gibson, B. S., & Morrissey, R. A. (2016). Combined cognitive and parent training interventions for adolescents with ADHD and their mothers: A randomized controlled trial. Child Neuropsychol, 22(4), 394-419. doi:10.1080/09297049.2014.994485 | | | | | | | No eligible control | | | No control | |  |
| Stevenson, C. S., Whitmont, S., Bornholt, L., Livesey, D., & Stevenson, R. J. (2002). A cognitive remediation programme for adults with Attention Deficit Hyperactivity Disorder. Aust N Z J Psychiatry, 36(5), 610-616. doi:10.1046/j.1440-1614.2002.01052.x | | | | | | | Does not meet definition of cognitive training according to meta-analysis protocol | | | Non-CCT | |  |
| Tamm, L., & Nakonezny, P. A. (2015). Metacognitive executive function training for young children with ADHD: a proof-of-concept study. Atten Defic Hyperact Disord, 7(3), 183-190. doi:10.1007/s12402-014-0162-x | | | | | | | Does not meet definition of cognitive training according to meta-analysis protocol | | | Non-CCT | |  |
| Tamm, L., Epstein, J. N., Peugh, J. L., Nakonezny, P. A., & Hughes, C. W. (2013). Preliminary data suggesting the efficacy of attention training for school-aged children with ADHD. Dev Cogn Neurosci, 4, 16-28. doi:10.1016/j.dcn.2012.11.004 | | | | | | | Does not meet definition of cognitive training according to meta-analysis protocol | | | Non-CCT | |  |
| Tamm, L., Hughes, C., Ames, L., Pickering, J., Silver, C. H., Stavinoha, P., . . . Emslie, G. (2010). Attention training for school-aged children with ADHD: results of an open trial. J Atten Disord, 14(1), 86-94. doi:10.1177/1087054709347446 | | | | | | | Non-randomised | | | Non-randomised | |  |
| Tullo, D., Guy, J., Faubert, J., & Bertone, A. (2018). Training with a three-dimensional multiple object-tracking (3D-MOT) paradigm improves attention in students with a neurodevelopmental condition: a randomized controlled trial. Developmental Science, 21(6), 11. doi:10.1111/desc.12670 | | | | | | | Mixed sample | | | Other | |  |
| van der Donk, M. L. A., van Viersen, S., Hiemstra-Beernink, A. C., Tjeenk-Kalff, A. C., van der Leij, A., & Lindauer, R. J. L. (2017). Individual Differences in Training Gains and Transfer Measures: An Investigation of Training Curves in Children with Attention-Deficit/Hyperactivity Disorder. Applied Cognitive Psychology, 31(3), 302-314. doi:10.1002/acp.3327 | | | | | | | Not controlled | | | No control | |  |
| van der Donk, M. L., Hiemstra-Beernink, A. C., Tjeenk-Kalff, A. C., van der Leij, A. V., & Lindauer, R. J. (2013). Interventions to improve executive functioning and working memory in school-aged children with AD(H)D: a randomised controlled trial and stepped-care approach. BMC Psychiatry, 13, 23. doi:10.1186/1471-244x-13-23 | | | | | | | Protocol | | | Other | |  |
| van der Donk, M. L., Hiemstra-Beernink, A. C., Tjeenk-Kalff, A. C., van der Leij, A., & Lindauer, R. J. (2016). Predictors and Moderators of Treatment Outcome in Cognitive Training for Children With ADHD. J Atten Disord. doi:10.1177/1087054716632876 | | | | | | | Both arms cognitive training | | | No control | |  |
| van der Donk, M., Hiemstra-Beernink, A. C., Tjeenk-Kalff, A., van der Leij, A., & Lindauer, R. (2015). Cognitive training for children with ADHD: a randomized controlled trial of cogmed working memory training and 'paying attention in class'. Front Psychol, 6, 1081. doi:10.3389/fpsyg.2015.01081 | | | | | | | Both arms cognitive training | | | No control | |  |
| Vanzin, L., et al. (2020). "Does ACT-Group Training Improve Cognitive Domain in Children with Attention Deficit Hyperactivity Disorder? A Single-Arm, Open-Label Study." Behaviour Change 37(1): 33-44. | | | | | | | Non-randomised | | | Non-randomised | |  |
| Wennberg, B., Janeslatt, G., Kjellberg, A., & Gustafsson, P. A. (2018). Effectiveness of time-related interventions in children with ADHD aged 9-15 years: a randomized controlled study. European Child & Adolescent Psychiatry, 27(3), 329-342. doi:10.1007/s00787-017-1052-5 | | | | | | | Does not meet definition of cognitive training according to meta-analysis protocol | | | Non-CCT | |  |
| Wexler, B. E., et al. (2021). "An integrated program of computer-presented and physical cognitive training exercises for children with attention-deficit/hyperactivity disorder." Psychol Med 51(9): 1524-1535. | | | | | | | Crossover trial | | | Non-parallel RCT | |  |
| Wexler, B. E., Vitulano, L. A., Moore, C., Katsovich, L., Smith, S. D., Rush, C., . . . Leckman, J. F. (2020). An integrated program of computer-presented and physical cognitive training exercises for children with attention-deficit/hyperactivity disorder. Psychol Med, 1-12. doi:10.1017/s0033291720000288 | | | | | | | Crossover trial | | | Non-parallel RCT | |  |
| Wiguna, T., et al. (2021). "Developing and feasibility testing of the Indonesian computer-based game prototype for children with attention deficit/hyperactivity disorder." Heliyon 7(7): 8. | | | | | | | Non-randomised | | | Non-randomised | |  |
| Zhang, D.-W., et al. (2021). "Comparing the transfer effects of three neurocognitive training protocols in children with attention-deficit/hyperactivity disorder: A single-case experimental design." Behaviour Change(Pagination). | | | | | | | Non-randomised | | | Non-randomised | |  |

| 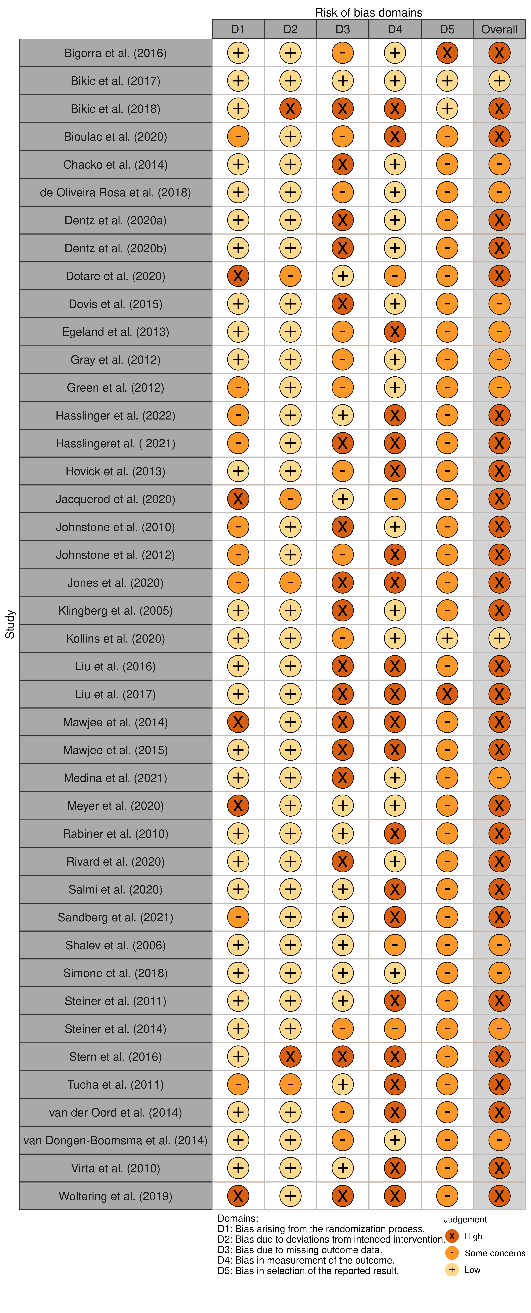 | 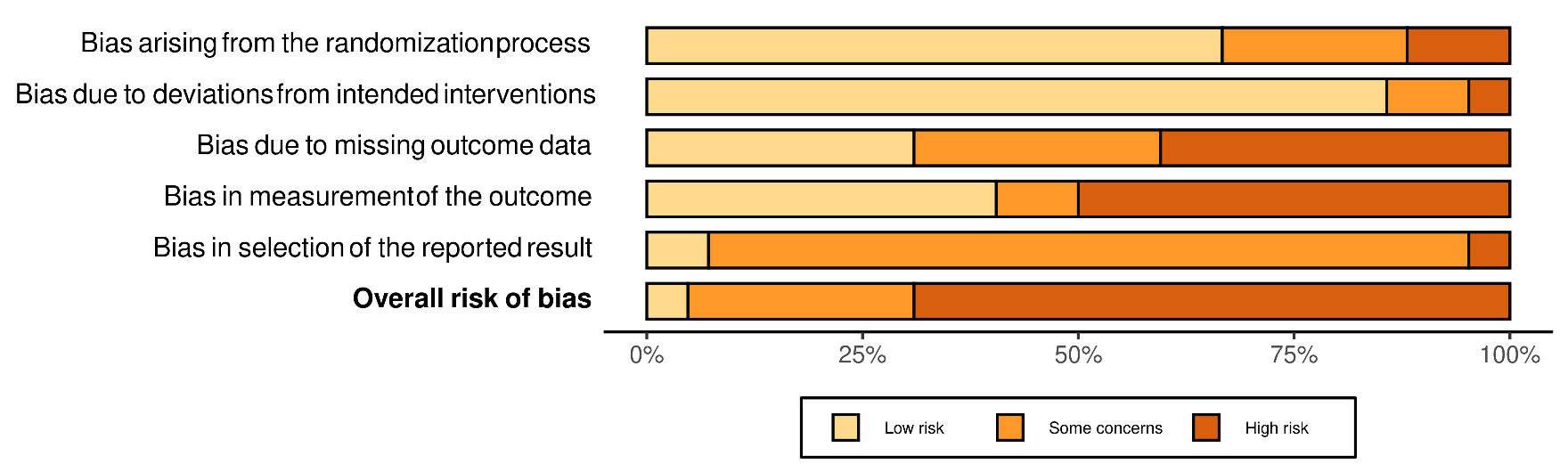 |
| --- | --- |
| Supplement Figure 2. Results from the Cochrane Risk of Bias 2.0 Tool. NB. detailed ratings of each paper are available upon request. For Domain 3, we judged reports as having low, some concerns, or high risks of bias if <5%, 5-10%, or >10% of data were missing after participants started the CCT protocol. | |

| Supplementary Table 5. A list of all proximally and probably blinded outcome measures of ADHD symptoms and executive functioning | | | | | | | | | | | | | | | | | | |
| --- | --- | --- | --- | --- | --- | --- | --- | --- | --- | --- | --- | --- | --- | --- | --- | --- | --- | --- |
| **Author** | | **Outcome** | **Dependent Variable** | | | | **Follow-Up Timepoint** | | | **Setting** | **Blinded (Y/N)** | | **Control** | | **Mprox** | | **Pblind** | |
| Bigorra et al. (2016) | | CRS-R(50) | ADHD Index | | | | Post-Ax | | | Home | Y | | NAC | | Parent | | Teacher | |
| Bigorra et al. (2016) | | CRS-R(50) | Cognitive Problems/Inattention | | | | Post-Ax | | | Home | Y | | NAC | | Parent | | Teacher | |
| Bigorra et al. (2016) | | BRIEF(51) | GEC | | | | Post-Ax | | | Home | Y | | NAC | | Parent | | Teacher | |
| Bigorra et al. (2016) | | CRS-R(50) | Hyperactivity/Impulsivity | | | | Post-Ax | | | Home | Y | | NAC | | Parent | | Teacher | |
|  | |  |  | | | |  | | |  |  | |  | |  | |  | |
|  | |  |  | | | |  | | |  |  | |  | |  | |  | |
|  | |  |  | | | |  | | |  |  | |  | |  | |  | |
| Bikic et al. (2018) | | ADHD-RS(52,53) | Combined Inattention & Hyperactivity/Impulsivity | | | | Post-Ax | | | Home | N | | TAU | | Parent | | Teacher (exc) | |
| Bikic et al. (2018) | | BRIEF(51) | GEC | | | | Post-Ax | | | Home | N | | TAU | | Parent | | Teacher (exc) | |
| Bikic et al. (2018) | | ADHD-RS(52,53) | Hyperactivity/Impulsivity | | | | Post-Ax | | | Home | N | | TAU | | Parent | | Teacher (exc) | |
| Bikic et al. (2018) | | ADHD-RS(52,53) | Inattention | | | | Post-Ax | | | Home | N | | TAU | | Parent | | Teacher (exc) | |
| Bioulac et al. (2020) | | ADHD-RS(54) | Hyperactivity/Impulsivity | | | | Post-Ax | | | Clinic/Lab | N | | AC | | Parent | | Parent (exc) | |
| Bioulac et al. (2020) | | ADHD-RS(54) | Inattention | | | | Post-Ax | | | Clinic/Lab | N | | AC | | Parent | | Parent (exc) | |
| Bioulac et al. (2020) | | ADHD-RS(54) | Total Score | | | | Post-Ax | | | Clinic/Lab | N | | AC | | Parent | | Parent (exc) | |
| Chacko et al. (2014) | | DBD-RS(55) | Combined Inattention & Hyperactivity/Impulsivity | | | | Post-Ax | | | Home | Y | | NAC | | Parent | | Teacher | |
| Chacko et al. (2014) | | DBD-RS(55) | Hyperactivity/Impulsivity | | | | Post-Ax | | | Home | Y | | NAC | | Parent | | Teacher | |
| Chacko et al. (2014) | | DBD-RS(55) | Inattention | | | | Post-Ax | | | Home | Y | | NAC | | Parent | | Teacher | |
| de Oliveira Rosa et al. (2018) | | SNAP-IV(56) | Combined Inattention & Hyperactivity/Impulsivity | | | | Post-Ax | | | Lab | Y | | AC | | Parent | | Teacher | |
| de Oliveira Rosa et al. (2018) | | SNAP-IV(56) | Hyperactivity/Impulsivity | | | | Post-Ax | | | Lab | Y | | AC | | Parent | | Teacher | |
| de Oliveira Rosa et al. (2018) | | SNAP-IV(56) | Inattention | | | | Post-Ax | | | Lab | Y | | AC | | Parent | | Teacher | |
| Dentz et al. (2020a) | | BRIEF(51) | GEC | | | | Post-Ax | | | Home | Y | | NAC | | Parent | | Parent | |
| Dentz et al. (2020a) | | BRIEF(51) | GEC | | | | FU-Ax | | | Home | Y | | NAC | | Parent | | Parent | |
| Dentz et al. (2020a) | | Conners 3AI(57) | Hyperactivity/Impulsivity | | | | Post-Ax | | | Home | Y | | NAC | | Parent | | Parent | |
| Dentz et al. (2020a) | | Conners 3AI(57) | Inattention | | | | Post-Ax | | | Home | Y | | NAC | | Parent | | Parent | |
| Dentz et al. (2020a) | | Conners 3AI(57) | Total | | | | Post-Ax | | | Home | Y | | NAC | | Parent | | Parent | |
| Dentz et al. (2020b) | | CAARS(58) | ADHD Index | | | | Post-Ax | | | Home | Y | | NAC | | Self | | Self | |
| Dentz et al. (2020b) | | CAARS(58) | Hyperactivity/Restlessness | | | | Post-Ax | | | Home | Y | | NAC | | Self | | Self | |
| Dentz et al. (2020b) | | CAARS(58) | Inattention/Memory Problems | | | | Post-Ax | | | Home | Y | | NAC | | Self | | Self | |
| Dovis et al. (2015) | | DBD-RS(55) | Combined Inattention & Hyperactivity/Impulsivity | | | | Post-Ax | | | Home | Y | | NAC | | Parent | | Teacher | |
| Dovis et al. (2015) | | DBD-RS(55) | Combined Inattention & Hyperactivity/Impulsivity | | | | FU-Ax | | | Home | Y | | NAC | | Parent | | Teacher | |
| Dovis et al. (2015) | | DBD-RS(55) | Hyperactivity/Impulsivity | | | | Post-Ax | | | Home | Y | | NAC | | Parent | | Teacher | |
| Dovis et al. (2015) | | DBD-RS(55) | Hyperactivity/Impulsivity | | | | FU-Ax | | | Home | Y | | NAC | | Parent | | Teacher | |
| Dovis et al. (2015) | | DBD-RS(55) | Inattention | | | | Post-Ax | | | Home | Y | | NAC | | Parent | | Teacher | |
| Dovis et al. (2015) | | DBD-RS(55) | Inattention | | | | FU-Ax | | | Home | Y | | NAC | | Parent | | Teacher | |
| Egeland et al. (2013) | | BRIEF(51) | GEC | | | | Post-Ax | | | School | N | | TAU | | Teacher | | Parent (exc) | |
| Egeland et al. (2013) | | BRIEF(51) | GEC | | | | FU-Ax | | | School | N | | TAU | | Teacher | | Parent (exc) | |
| Egeland et al. (2013) | | ADHD-RS(54) | Hyperactivity/Impulsivity | | | | Post-Ax | | | School | N | | TAU | | Teacher | | Parent (exc) | |
| Egeland et al. (2013) | | ADHD-RS(54) | Hyperactivity/Impulsivity | | | | FU-Ax | | | School | N | | TAU | | Teacher | | Parent (exc) | |
| Egeland et al. (2013) | | ADHD-RS(54) | Inattention | | | | Post-Ax | | | School | N | | TAU | | Teacher | | Parent (exc) | |
| Egeland et al. (2013) | | ADHD-RS(54) | Inattention | | | | FU-Ax | | | School | N | | TAU | | Teacher | | Parent (exc) | |
| Egeland et al. (2013) | | ADHD-RS(54) | Total Score | | | | Post-Ax | | | School | N | | TAU | | Teacher | | Parent (exc) | |
| Egeland et al. (2013) | | ADHD-RS(54) | Total Score | | | | FU-Ax | | | School | N | | TAU | | Teacher | | Parent (exc) | |
| Gray et al. (2012) | | SWAN(59) | Hyperactivity/Impulsivity | | | | Post-Ax | | | School | N | | AC | | Teacher | | Parent (exc) | |
| Gray et al. (2012) | | SWAN(59) | Inattention | | | | Post-Ax | | | School | N | | AC | | Teacher | | Parent (exc) | |
| Gray et al. (2012) | | SWAN(59) | Total | | | | Post-Ax | | | School | N | | AC | | Teacher | | Parent (exc) | |
| Green et al. (2012) | | CPRS-R* | ADHD Index | | | | Post-Ax | | | Home | Y | | NAC | | Parent | | Parent | |
| Green et al. (2012) | | CPRS-R* | DSM Hyperpactivity/Impulsivity | | | | Post-Ax | | | Home | Y | | NAC | | Parent | | Parent | |
| Green et al. (2012) | | CPRS-R* | DSM Inattention | | | | Post-Ax | | | Home | Y | | NAC | | Parent | | Parent | |
| Hasslinger et al. (2021) | | CRS-3(60) | ADHD Index | | | | Post-Ax | | | Clinic | N | | TAU | | Parent | | Teacher (exc) | |
| Hasslinger et al. (2021) | | CRS-3(60) | ADHD Index | | | | FU-Ax | | | Clinic | N | | TAU | | Parent | | Teacher (exc) | |
| Hasslinger et al. (2021) | | CRS-3(60) | DSM Hyperpactivity/Impulsivity | | | | Post-Ax | | | Clinic | N | | TAU | | Parent | | Teacher (exc) | |
| Hasslinger et al. (2021) | | CRS-3(60) | DSM Hyperpactivity/Impulsivity | | | | FU-Ax | | | Clinic | N | | TAU | | Parent | | Teacher (exc) | |
| Hasslinger et al. (2021) | | CRS-3(60) | DSM Inattention | | | | Post-Ax | | | Clinic | N | | TAU | | Parent | | Teacher (exc) | |
| Hasslinger et al. (2021) | | CRS-3(60) | DSM Inattention | | | | FU-Ax | | | Clinic | N | | TAU | | Parent | | Teacher (exc) | |
| Hasslinger et al. (2021) | | BRIEF(51) | GEC | | | | Post-Ax | | | Clinic | N | | TAU | | Parent | | Teacher (exc) | |
| Hasslinger et al. (2021) | | BRIEF(51) | GEC | | | | FU-Ax | | | Clinic | N | | TAU | | Parent | | Teacher (exc) | |
| Jones et al. (2020) | | CPRS-R:L(61,62) | ADHD Index | | | | FU-Ax | | | Home/Lab | Y | | AC | | Parent | | Parent | |
| Jones et al. (2020) | | CPRS-R:L(61,62) | DSM - Hyperactivity | | | | FU-Ax | | | Home/Lab | Y | | AC | | Parent | | Parent | |
| Jones et al. (2020) | | CPRS-R:L(61,62) | DSM - Inattentiion | | | | FU-Ax | | | Home/Lab | Y | | AC | | Parent | | Parent | |
| Jones et al. (2020) | | BRIEF(51) | GEC | | | | FU-Ax | | | Home/Lan | Y | | AC | | Parent | | Parent | |
| Klingberg et al. (2005) | | CPRS-R/T:S(61) | Combined (Inattention & Hyperactivity/Impulsivity) | | | | Post-Ax | | | Home/School | Y | | NAC | | Parent | | Teacher | |
| Klingberg et al. (2005) | | CPRS-R/T:S(61) | Combined (Inattention & Hyperactivity/Impulsivity) | | | | FU-Ax | | | Home/School | Y | | NAC | | Parent | | Teacher | |
| Klingberg et al. (2005) | | CPRS-R/T:S(61) | Hyperactivity/Impulsivity | | | | Post-Ax | | | Home/School | Y | | NAC | | Parent | | Teacher | |
| Klingberg et al. (2005) | | CPRS-R/T:S(61) | Hyperactivity/Impulsivity | | | | FU-Ax | | | Home/School | Y | | NAC | | Parent | | Teacher | |
| Klingberg et al. (2005) | | CPRS-R/T:S(61) | Inattention | | | | Post-Ax | | | Home/School | Y | | NAC | | Parent | | Teacher | |
| Klingberg et al. (2005) | | CPRS-R/T:S(61) | Inattention | | | | FU-Ax | | | Home/School | Y | | NAC | | Parent | | Teacher | |
| Kollins et al. (2020) | | BRIEF(51) | GEC | | | | Post-Ax | | | Home | N | | AC | | Parent | | Parent | |
| Medina et al. (2021) | | EDAH(63) | ADHD Index | | | | Post-Ax | | | Home | Y | | AC | | Parent | | Parent | |
| Medina et al. (2021) | | EDAH(63) | DSM Hyperactivity/Impulsivity | | | | Post-Ax | | | Home | Y | | AC | | Parent | | Parent | |
| Medina et al. (2021) | | EDAH(63) | DSM Inattention | | | | Post-Ax | | | Home | Y | | AC | | Parent | | Parent | |
| Medina et al. (2021) | | BRIEF* | GEC | | | | Post-Ax | | | Home | Y | | AC | | Parent | | Parent | |
| Meyer et al. (2020) | | CTRS-R(64) | Combined (Inattention & Hyperactivity) | | | | Post-Ax | | | Home | Y | | AC | | Teacher (exc) | | Teacher | |
| Meyer et al. (2020) | | SNAP-IV | Combined (Inattention & Hyperactivity/Impulsivity) | | | | Post-Ax | | | Home | Y | | AC | | Parent | | Parent (exc) | |
| Meyer et al. (2020) | | SNAP-IV(65) | Hyperacitivity/Impulsivity | | | | Post-Ax | | | Home | Y | | AC | | Parent | | Parent (exc) | |
| Meyer et al. (2020) | | SNAP-IV(65) | Hyperactivity | | | | Post-Ax | | | Home | Y | | AC | | Teacher (exc) | | Teacher | |
| Meyer et al. (2020) | | SNAP-IV(65) | Inattention | | | | Post-Ax | | | Home | Y | | AC | | Parent | | Parent (exc) | |
| Meyer et al. (2020) | | SNAP-IV(65) | Inattention | | | | Post-Ax | | | Home | Y | | AC | | Teacher (exc) | | Teacher | |
| Rabiner et al. (2010) | | CTRS-R:L(50) | DSM Inattention | | | | Post-Ax | | | School | N | | WLC | | Teacher | | Teacher (exc) | |
| Salmi et al. (2020) | | ASRS^14^ | Total Score | | | | Post-Ax | | | Lab/Home | N | | AC | | Self | | Self (exc) | |
| Sandberg et al. (2021) | | CBCL(66) | ADHD Total | | | | Post-Ax | | | Hospital | N | | WLC | | Parent | | Parent (exc) | |
| Sandberg et al. (2021) | | BRIEF(67) | GEC | | | | Post-Ax | | | Clinic | N | | TAU | | Parent | | Teacher (exc) | |
| Shalev et al. (2006) | | ADHD-RS(54) | Combined (Inattention & Hyperactivity/Impulsivity) | | | | Post-Ax | | | Lab | Y | | AC | | Parent | | Parent | |
| Shalev et al. (2006) | | ADHD-RS(54) | Hyperactivity/Impulsivitiy | | | | Post-Ax | | | Lab | Y | | AC | | Parent | | Parent | |
| Shalev et al. (2006) | | ADHD-RS(54) | Inattention | | | | Post-Ax | | | Lab | Y | | AC | | Parent | | Parent | |
| Steiner et al. (2011) | | CRS-R(50,62,68) | ADHD Index | | | | Post-Ax | | | School | N | | WLC | | Parent #1 Mother | | Blinded Teacher | |
| Steiner et al. (2011) | | BRIEF(51) | GEC | | | | Post-Ax | | | School | N | | AC | | Parent #1 | | Parent #1 (exc) | |
| Steiner et al. (2011) | | CRS-R(50,62,68) | Hyperactivity | | | | Post-Ax | | | School | N | | WLC | | Parent #1 Mother | | Blinded Teacher | |
| Steiner et al. (2011) | | CRS-R(50,62,68) | Memory Problems/Inattention | | | | Post-Ax | | | School | N | | WLC | | Parent #1 Mother | | Blinded Teacher | |
| Steiner et al. (2014) | | BOSS(69) | Combined (Motor/Verbal & Passive Off-Task) | | | | Post-Ax | | | School | N | | WLC | | na | | Blinded Independent Observer | |
| Steiner et al. (2014) | | Conners 3P(70) | DSM-IV Inattention | | | | Post-Ax | | | School | N | | WLC | | Parent | | Parent (exc) | |
| Steiner et al. (2014) | | BRIEF(51) | GEC | | | | Post-Ax | | | School | N | | WLC | | Parent | | Parent (exc) | |
| Steiner et al. (2014) | | BOSS(69) | Motor/Verbal Off-Task | | | | Post-Ax | | | School | N | | WLC | | na | | Blinded Independent Observer | |
| Steiner et al. (2014) | | BOSS(69) | Passive Off-Task | | | | Post-Ax | | | School | N | | WLC | | na | | Blinded Independent Observer | |
| Stern et al. (2016) | | BRIEF(71,72) | GEC | | | | Post-Ax | | | Home | N | | WLC | | Self | | Self (exc) | |
| Stern et al. (2016) | | ASRS-v1(73) | Total Score | | | | Post-Ax | | | Home | N | | WLC | | Self | | Self (exc) | |
| van der Oord et al. (2014) | | DBD-RS(55) | Combined Inattention & Hyperactivity/Impulsivity | | | | Post-Ax | | | Home | N | | WLC | | Parent | | Teacher (exc) | |
| van der Oord et al. (2014) | | BRIEF(51) | GEC | | | | Post-Ax | | | Home | N | | WLC | | Parent | | Parent (exc) | |
| van der Oord et al. (2014) | | DBD-RS(55) | Hyperactivity/Impulsivity | | | | Post-Ax | | | Home | N | | WLC | | Parent | | Teacher (exc) | |
| van der Oord et al. (2014) | | DBD-RS(55) | Inattention | | | | Post-Ax | | | Home | N | | WLC | | Parent | | Teacher (exc) | |
| van Dongen-Boomsma et al. (2014) | | BRIEF(51,74) | GEC | | | | Post-Ax | | | Home | nr | | NAC | | Parent | | Teacher | |
| van Dongen-Boomsma et al. (2014) | | ADHD-RS(54) | Hyperactivity/Impulsivity | | | | Post-Ax | | | Home | nr | | NAC | | Investigator | | Teacher | |
| van Dongen-Boomsma et al. (2014) | | ADHD-RS(54) | Inattention | | | | Post-Ax | | | Home | nr | | NAC | | Investigator | | Teacher | |
| van Dongen-Boomsma et al. (2014) | | ADHD-RS(54) | Total Score | | | | Post-Ax | | | Home | nr | | NAC | | Investigator | | Teacher | |
| Virta et al. (2010) | | ASRS-v1(73) | Total | | | | Post-Ax | | | Clinic/Lab | N | | AC | | Self | | Blinded Indepedent Evaluator (no data available) | |
| Woltering et al. (2019) | | ASRS(75) | Total | | | | Post-Ax | | | Home | N | | WLC | | Self | | Self (exc) | |
| Legend. AC, active control; ADHD-RS, ADHD-Rating Scale; ASRS, Adult ADHD Self-Report Scale; BOSS, Behavioral Observation of Students in Schools; BRIEF, Behavior Rating Inventory of Executive Function; CBCL, Child Behaviour Checklist; CP/TRS-R:L, Conners’ Parent/Teacher Questionnaire–Revised: Long Form; CPR/TS-R:S, Conners’ Parent/Teacher Questionnaire–Revised: Short Form; CRS-3, Conners Rating Scale 3rd Edition; CRS-3, Conners Rating Scale 3rd Edition; CRS-R, Conners' Rating Scale - Revised; DBD-RS, Disruptive Behavior Disorder Rating Scales; DSM, Diagnostic Statistical Manual of Mental Healthy Disorders; EDAH, Evaluation of Attention Deficit and Hyperactivity Disorder; exc, excluded because non-blinded; GEC, Global Executive Composite; NAC, non-adaptive control; nr, not reported; SNAP-IV, Swanson, Nolan, and Pelham–IV Questionnaire; SWAN, Strengths and Weaknesses of ADHD-symptoms and Normal-behavior; TAU, treatment-as-usual; WLC, waiting list control. *no reference given | | | | | | | | | | | | | | | | | | |
| Supplementary Table 6. Summary of results showing pooled standardized mean differences (SMD; with Hedges’ *g* adjustment) between treatment and control arms for MPROX measures of ADHD symptoms and executive functions collected at the first assessment after the final CCT session and at follow-up (*M*, 6-months; range, 3- to 6-months). Significant values are bolded. | | | | | | | | | | | | | | | | |  |  |
|  | | |  |  |  | | **Effect Size Estimate** | | | | | | **Heterogeneity** | | | |  |  |
| **Outcome** | | | **Trials Included** | **Study N** | **Total N** | | **SMD** | **95%CI** | | | ***p*** | | **I^2^** | | ***p**** | |  |  |
| **ADHD Symptoms** | | |  |  |  | |  |  | | |  | |  | |  | |  |  |
| ADHD Total | | | All | 24 | 1133 | | **0.17** | **0.03 to 0.31** | | | **0.02** | | **42** | | **0.02** | |  |  |
|  | | | Active control | 17 | 743 | | 0.09 | -0.07 to 0.25 | | | 0.27 | | 34 | | 0.09 | |  |  |
|  | | | MED | 9 | 392 | | 0.13 | -0.10 to 0.35 | | | 0.27 | | 42 | | 0.09 | |  |  |
|  | | | WMT | 13 | 697 | | 0.02 | -0.13 to 0.17 | | | 0.57 | | 2 | | 0.42 | |  |  |
|  | | | MPT | 8 | 325 | | **0.34** | **0.08 to 0.60** | | | **0.01** | | 48 | | 0.06 | |  |  |
|  | | | Children/adolescents | 19 | 936 | | **0.17** | **-0.00 to 0.34** | | | **0.05** | | **54** | | **0.003** | |  |  |
|  | | | Adults | 5 | 197 | | 0.16 | -0.13 to 0.44 | | | 0.28 | | 0 | | 0.90 | |  |  |
|  | | | Non-commercially funded | 20 | 971 | | 0.13 | -0.03 to 0.30 | | | 0.10 | | 48 | | **0.008** | |  |  |
| Inattention | | | All | 21 | 1061 | | **0.28** | **0.13 to 0.42** | | | **<0.001** | | 28 | | 0.11 | |  |  |
|  | | | Active control | 15 | 663 | | **0.23** | **0.04 to 0.42** | | | **0.02** | | 32 | | 0.11 | |  |  |
|  | | | MED | 8 | 417 | | 0.26 | -0.03 to 0.55 | | | 0.08 | | **52** | | **0.04** | |  |  |
|  | | | WMT | 10 | 564 | | **0.23** | **0.02 to 0.43** | | | **0.03** | | 30 | | 0.17 | |  |  |
|  | | | MPT | 7 | 334 | | **0.35** | **0.11 to 0.59** | | | **0.004** | | 27 | | 0.22 | |  |  |
|  | | | Children/adolescents | 20 | 957 | | **0.27** | **0.12 to 0.41** | | | **<0.001** | | 30 | | 0.1 | |  |  |
|  | | | Adults | 1 | na | | na | na | | | na | | na | | na | |  |  |
|  | | | Non-commercially funded | 18 | 939 | | **0.23** | **0.08 to 0.38** | | | **0.002** | | 25 | | 0.17 | |  |  |
| Hyperactivity/ Impulsivity | | | All | 19 | 937 | | 0.09 | -0.04 to 0.21 | | | 0.2 | | 0 | | 0.71 | |  |  |
|  | | | Active control | 15 | 663 | | 0.01 | -0.15 to 0.16 | | | 0.93 | | 0 | | 0.96 | |  |  |
|  | | | MED | 7 | 308 | | -0.02 | -0.24 to 0.21 | | | 0.88 | | 0 | | 0.48 | |  |  |
|  | | | WMT | 10 | 562 | | 0.02 | -0.15 to 0.19 | | | 0.82 | | 0 | | 0.97 | |  |  |
|  | | | MPT | 6 | 264 | | 0.24 | -0.04 to 0.51 | | | 0.09 | | 20 | | 0.28 | |  |  |
|  | | | Children/adolescents | 18 | 893 | | 0.09 | -0.05 to 0.22 | | | 0.21 | | 0 | | 0.64 | |  |  |
|  | | | Adults | 1 | na | | na | na | | | na | | na | | na | |  |  |
|  | | | Non-commercially funded | 16 | 815 | | 0.07 | -0.07 to 0.21 | | | 0.35 | | 0 | | 0.54 | |  |  |
| **Neuropsychological** | | |  |  |  | |  |  | | |  | |  | |  | |  |  |
| BRIEF-GEC | | | All | 13 | 914 | | 0.08 | -0.05 to 0.21 | | | 0.24 | | 0 | | 0.76 | |  |  |
|  | | | Active control | 7 | 531 | | -0.02 | -0.19 to 0.15 | | | 0.78 | | 0 | | 0.86 | |  |  |
|  | | | MED | 4 | na | | na | na | | | na | | na | | na | |  |  |
|  | | | WMT | 6 | 323 | | 0.04 | -0.17 to 0.26 | | | 0.69 | | 0 | | 0.8 | |  |  |
|  | | | MPT | 7 | 591 | | 0.1 | -0.06 to 0.26 | | | 0.24 | | 0 | | 0.44 | |  |  |
|  | | | Children/adolescents | 12 | 875 | | 0.08 | -0.05 to 0.22 | | | 0.22 | | 0 | | 0.70 | |  |  |
|  | | | Adults | 1 | na | | na | na | | | na | | na | | na | |  |  |
|  | | | Non-commercially funded | 10 | 524 | | 0.12 | -0.05 to 0.29 | | | 0.16 | | 0 | | 0.69 | |  |  |
| **Follow-Up Outcomes** | | |  |  |  | |  |  | | |  | |  | |  | |  |  |
| ADHD Total | | | All | 7 | 387 | | 0.15 | -0.06 to 0.36 | | | 0.16 | | 23 | | 0.25 | |  |  |
|  | | | Active control | 4 | na | | na | na | | | na | | na | | na | |  |  |
|  | | | MED | 4 | na | | na | na | | | na | | na | | na | |  |  |
|  | | | WMT | 6 | 326 | | 0.18 | -0.08 to 0.4 | | | 0.17 | | 33 | | 0.19 | |  |  |
|  | | | MPT | 1 | na | | na | na | | | na | | na | | na | |  |  |
|  | | | Children/adolescents | 7 | 387 | | 0.15 | -0.06 to 0.36 | | | 0.16 | | 23 | | 0.25 | |  |  |
|  | | | Adults | 0 | na | | na | na | | | na | | na | | na | |  |  |
|  | | | Non-commercially funded | 6 | 347 | | 0.10 | -0.10 to 0.30 | | | 0.32 | | 11 | | 0.35 | |  |  |
| Inattention | | | All | 6 | 347 | | 0.16 | -0.05 to 0.37 | | | 0.14 | | 0 | | 0.42 | |  |  |
|  | | | Active control | 4 | na | | na | na | | | na | | na | | na | |  |  |
|  | | | MED | 3 | na | | na | na | | | na | | na | | na | |  |  |
|  | | | WMT | 5 | 286 | | 0.17 | -0.10 to 0.43 | | | 0.22 | | 19 | | 0.29 | |  |  |
|  | | | MPT | 1 | na | | na | na | | | na | | na | | na | |  |  |
|  | | | Children/adolescents | 6 | 347 | | 0.16 | -0.05 to 0.37 | | | 0.14 | | 0 | | 0.42 | |  |  |
|  | | | Adults | 0 | na | | na | na | | | na | | na | | na | |  |  |
|  | | | Non-commercially funded | 6 | 347 | | 0.16 | -0.05 to 0.37 | | | 0.14 | | 0 | | 0.42 | |  |  |
| Hyperactivity/Impulsivity | | | All | 6 | 347 | | 0.07 | -0.14 to 0.28 | | | 0.50 | | 0 | | 0.76 | |  |  |
|  | | | Active control | 4 | na | | na | na | | | na | | na | | na | |  |  |
|  | | | MED | 3 | na | | na | na | | | na | | na | | na | |  |  |
|  | | | WMT | 5 | 286 | | 0.09 | -0.14 to 0.32 | | | 0.44 | | 0 | | 0.65 | |  |  |
|  | | | MPT | 1 | na | | na | na | | | na | | na | | na | |  |  |
|  | | | Children/adolescents | 6 | 347 | | 0.07 | -0.14 to 0.28 | | | 0.50 | | 0 | | 0.76 | |  |  |
|  | | | Adults | 0 | na | | na | na | | | na | | na | | na | |  |  |
|  | | | Non-commercially funded | 6 | 347 | | 0.07 | -0.14 to 0.28 | | | 0.50 | | 0 | | 0.76 | |  |  |
| BRIEF-GEC | | | All | 6 | 321 | | **0.24** | **0.02 to 0.45** | | | **0.03** | | 0 | | 0.8 | |  |  |
|  | | | Active control | 3 | na | | na | na | | | na | | na | | na | |  |  |
|  | | | MED | 1 | na | | na | na | | | na | | na | | na | |  |  |
|  | | | WMT | 6 | 321 | | **0.24** | **0.02 to 0.45** | | | **0.03** | | 0 | | 0.8 | |  |  |
|  | | | MPT | 0 | na | | na | na | | | na | | na | | na | |  |  |
|  | | | Children/adolescents | 6 | 321 | | **0.24** | **0.02 to 0.45** | | | **0.03** | | 0 | | 0.8 | |  |  |
|  | | | Adults | 0 | na | | na | na | | | na | | na | | na | |  |  |
|  | | | Non-commercially funded | 5 | 281 | | 0.18 | -0.05 to 0.40 | | | 0.12 | | 0 | | 1.00 | |  |  |
| *p-values from Q – i.e., the chi-squared test statistic; ADHD, attention-deficit/hyperactivity disorder; BRIEF-GEC, Behavior Rating Inventory of Executive Function - Global Executive Composite; CI, Confidence Intervals; I^2^, percentage of between-study variation across SMDs that is due to heterogeneity rather than chance; MED, only a minority (i.e., <30%) of participants were receiving medication; MPROX, most proximal; MPT, multi-process training; N, sample size; SMD, Hedges’ *g*; WMT, working memory training | | | | | | | | | | | | | | | | |  |  |

| **Combined Inattention & Hyperactivity/Impulsivity Symptoms** |
| --- |
| 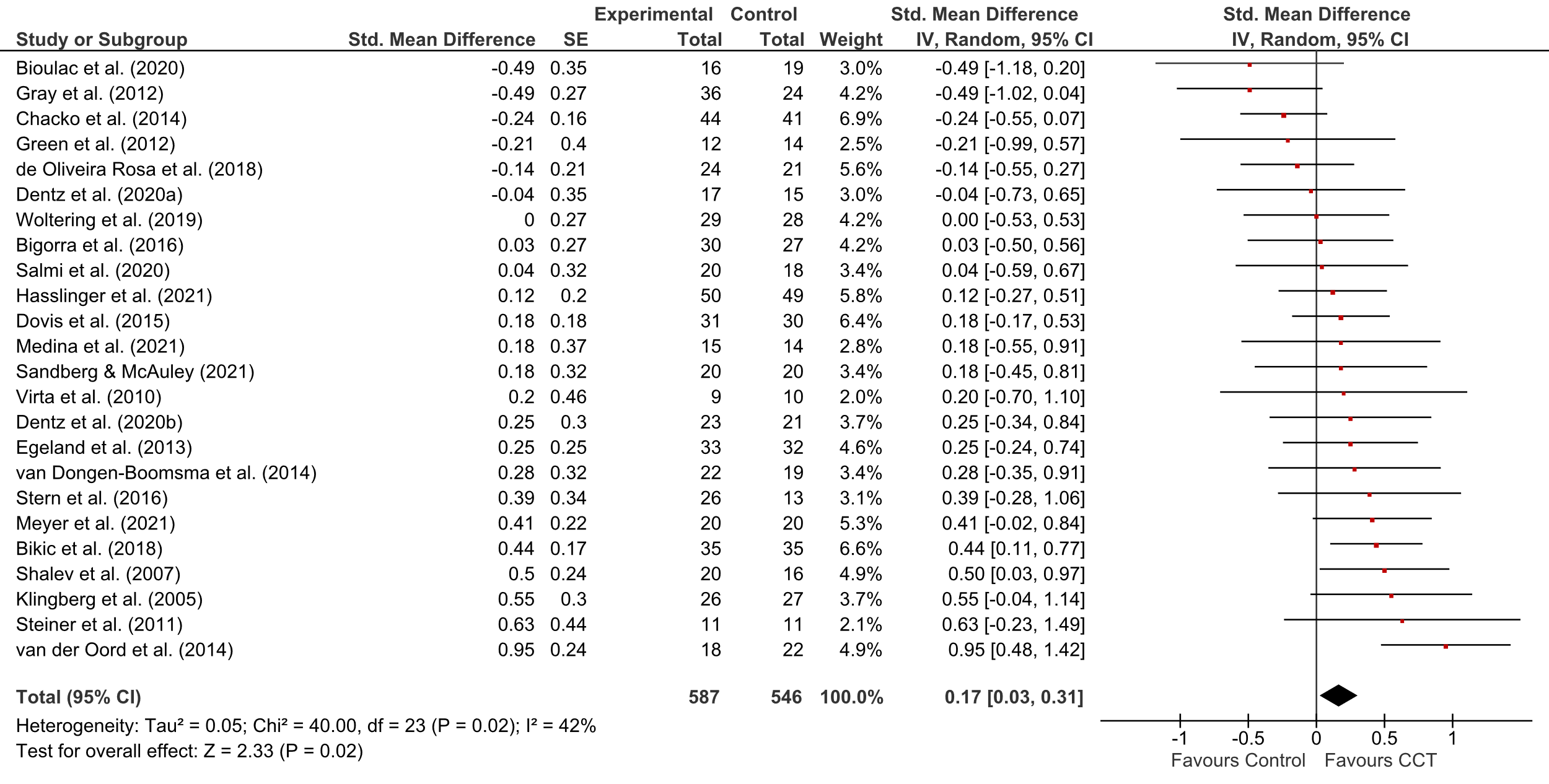 |
| **Inattention Symptoms** |
| 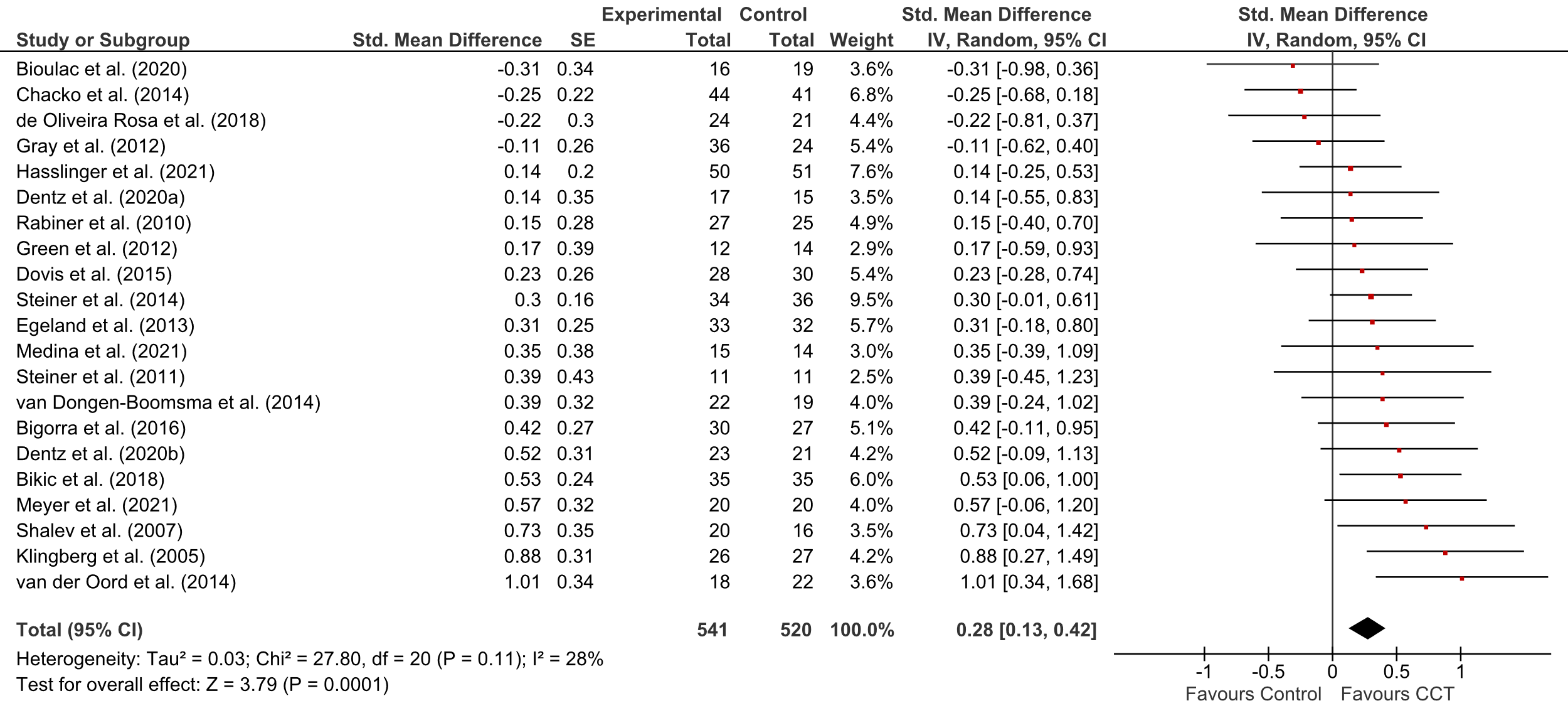 |
| **Hyperactivity/Impulsivity Symptoms** |
| 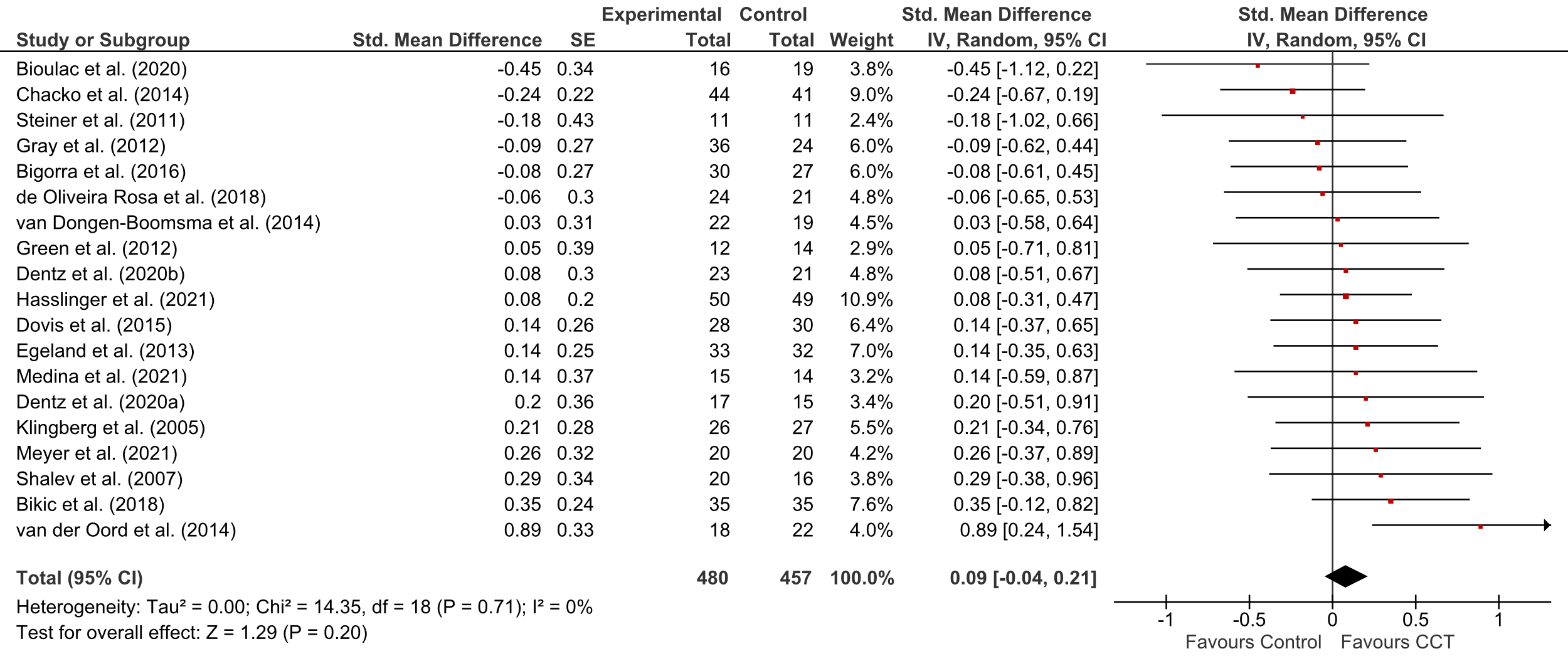 |
| *Supplementary Figure 3. Forest plots for meta-analysis of effects MPROX outcome measures of ADHD Total, Inattention or Hyperactivity/Impulsivity symptoms at the first assessment post treatment. Note. CCT = Computerised Cognitive Training, SE = Standard Error, Std. = Standardised* |

| **Combined Inattention & Hyperactivity/Impulsivity Symptoms** |
| --- |
| 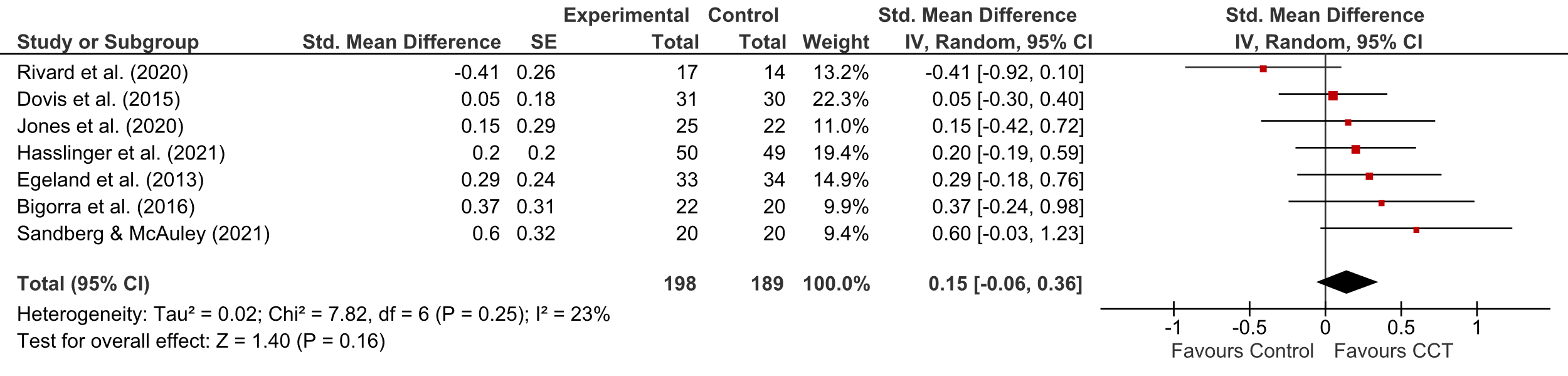 |
| **Inattention Symptoms** |
| 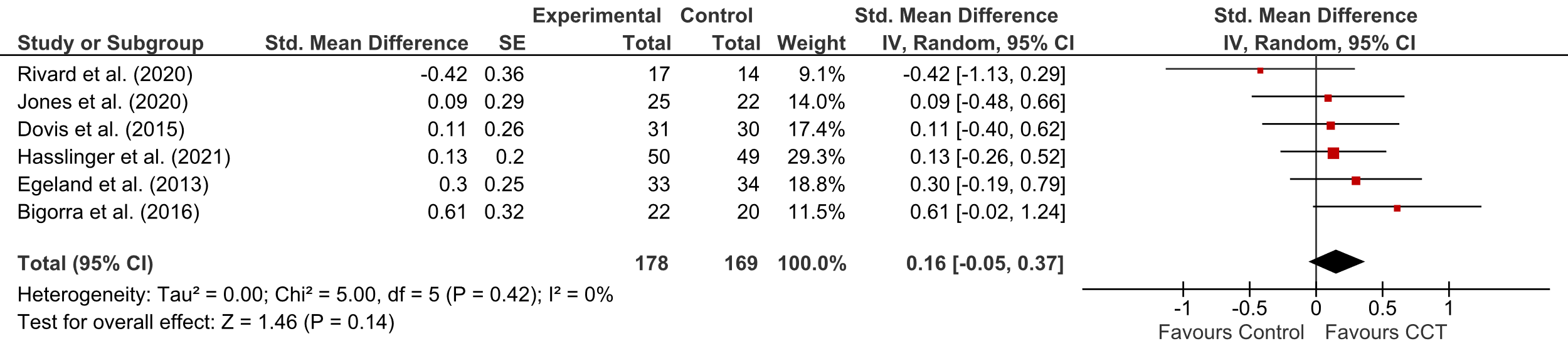 |
| **Hyperactivity/Impulsivity Symptoms** |
| 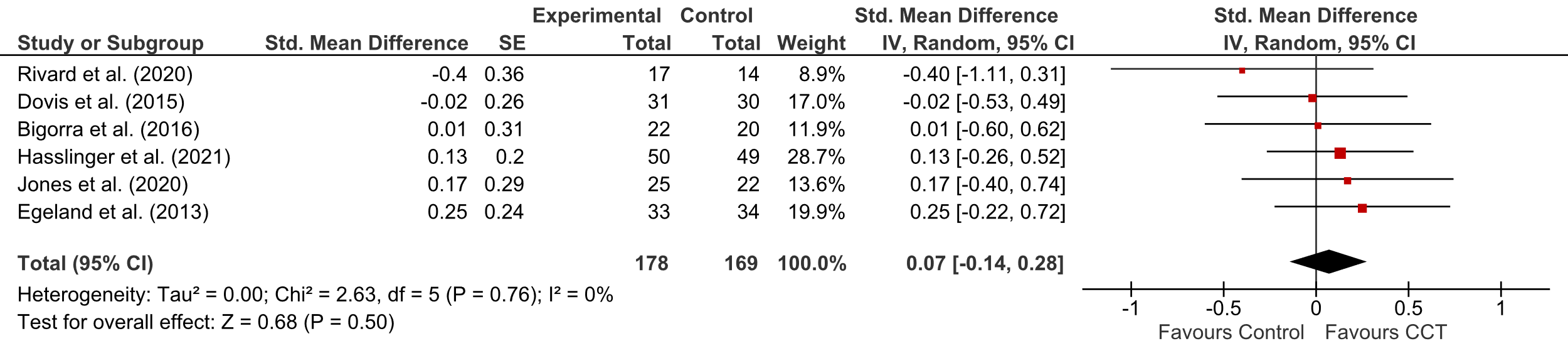 |
| *Supplementary Figure 4. Forest plots for meta-analysis of effects MPROX outcome measures of ADHD Total, Inattention or Hyperactivity/Impulsivity symptoms at follow-up (M, 6-months; range, 3- to 6-months). Note. CCT = Computerised Cognitive Training, SE = Standard Error, Std. = Standardised* |

| Supplementary Table 7. Summary of results showing pooled standardized mean differences (SMD; with Hedges’ *g* adjustment) between treatment and control arms for laboratory measures of neuropsychological and academic outcomes measured at follow-up (*M*, 6-months; range, 3- to 6-months). Significant values are bolded. | | | | | | | | |
| --- | --- | --- | --- | --- | --- | --- | --- | --- |
|  |  |  |  | **Effect Size Estimate** | | | **Heterogeneity** | |
| **Outcome** | **Trials Included** | **Study N** | **Total N** | **SMD** | **95%CI** | ***p*** | **I^2^** | ***p**** |
| **Neuropsychological** |  |  |  |  |  |  |  |  |
| Attention | All | 5 | 314 | **0.08** | **-0.22 to 0.38** | **0.61** | 42 | 0.14 |
|  | Active control | 2 | na | na | na | na | na | na |
|  | MED | 4 | na | na | na | na | na | na |
|  | WMT | 4 | na | na | na | na | na | na |
|  | MPT | 1 | na | na | na | na | na | na |
|  | Children/adolescents | 5 | 314 | **0.08** | **-0.22 to 0.38** | **0.61** | 42 | 0.14 |
|  | Adults | 0 | na | na | na | na | na | na |
|  | Non-commercially funded | 0 | na | na | na | na | na | na |
| Motor Inhibition | All | 7 | 427 | **0.24** | **0.05 to 0.43** | **0.01** | 0 | 0.61 |
|  | Active control | 4 | na | na | na | na | na | na |
|  | MED | 5 | 324 | 0.19 | -0.03 to 0.41 | 0.08 | 0 | 0.52 |
|  | WMT | 5 | 296 | 0.20 | -0.03 to 0.43 | 0.08 | 0 | 0.52 |
|  | MPT | 2 | na | na | na | na | na | na |
|  | Children/adolescents | 7 | 427 | **0.24** | **0.05 to 0.43** | **0.01** | 0 | 0.61 |
|  | Adults | 0 | na | na | na | na | na | na |
|  | Non-commercially funded | 7 | 427 | **0.24** | **0.05 to 0.43** | **0.01** | 0 | 0.61 |
| Verbal WM | All | 6 | 360 | **0.32** | **0.15 to 0.49** | **<0.001** | 0 | 0.94 |
|  | Active control | 4 | na | na | na | na | na | na |
|  | MED | 4 | na | na | na | na | na | na |
|  | WMT | 5 | 299 | **0.32** | **0.14 to 0.50** | **<0.001** | 0 | 0.88 |
|  | MPT | 1 | na | na | na | na | na | na |
|  | Children/adolescents | 6 | 360 | **0.32** | **0.15 to 0.49** | **<0.001** | 0 | 0.94 |
|  | Adults | 0 | na | na | na | na | na | na |
|  | Non-commercially funded | 5 | 319 | **0.30** | **0.13 to 0.48** | **<0.001** | 0 | 0.94 |
| **Academic** |  |  |  |  |  |  |  |  |
| Reading Comprehension | All | 5 | 134 | **0.26** | **0.00 to 0.52** | **0.05** | 0 | 0.43 |
|  | Active control | na | na | na | na | na | na | na |
|  | MED | na | na | na | na | na | na | na |
|  | WMT | na | na | na | na | na | na | na |
|  | MPT | na | na | na | na | na | na | na |
|  | Children/adolescents | na | na | na | na | na | na | na |
|  | Adults | na | na | na | na | na | na | na |
|  | Non-commercially funded | na | na | na | na | na | na | na |
| *p-values from Q – i.e., the chi-squared test statistic; ADHD, attention-deficit/hyperactivity disorder; CI, Confidence Intervals; I^2^, percentage of between-study variation across SMDs that is due to heterogeneity rather than chance; MED, only a minority (i.e., <30%) of participants were receiving medication; MPT, multi-process training; N, sample size; SMD, Hedges’ *g*;; WM, working memory; WMT, working memory training | | | | | | | | |

| **Neurocognitive Outcomes** | |
| --- | --- |
| BRIEF – GEC | BRIEF – GEC at follow-up |
| 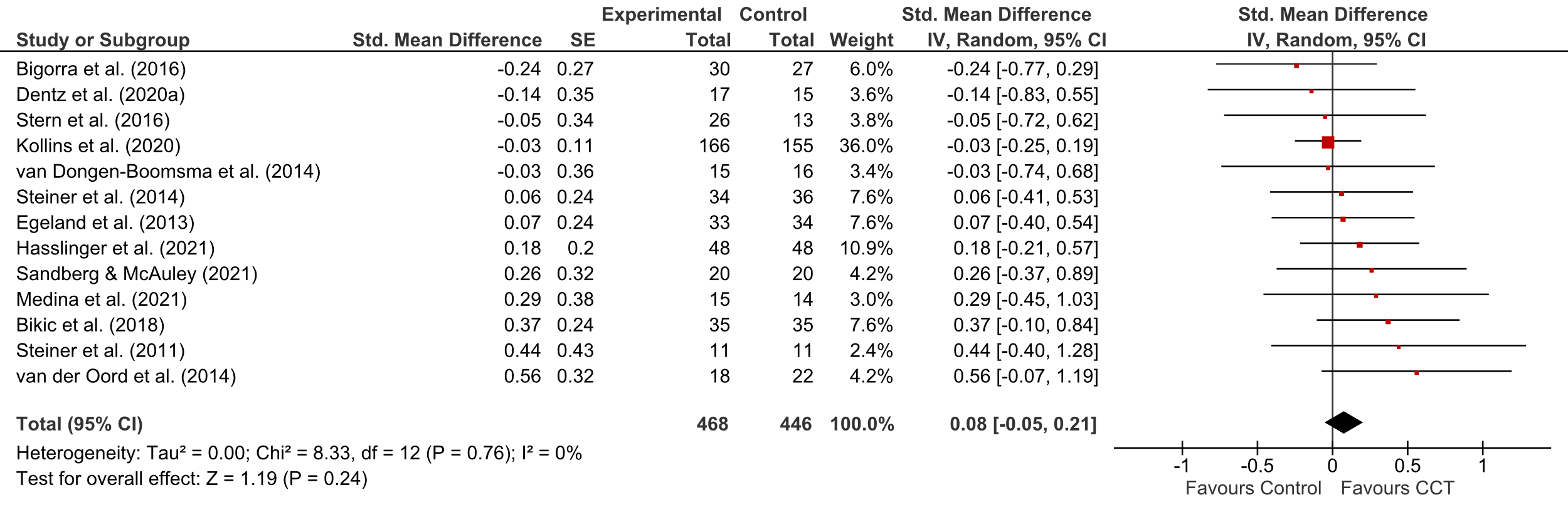 | 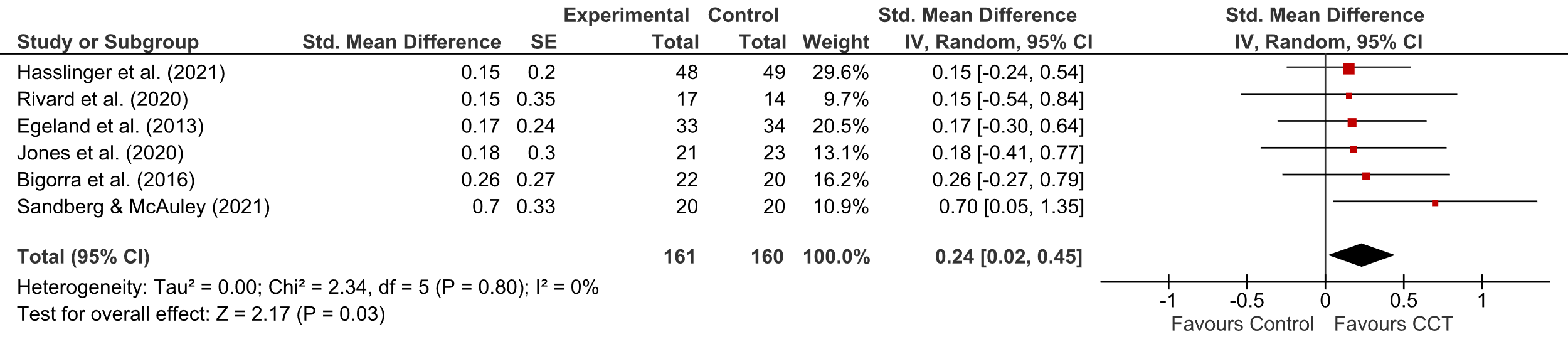 |
| Attention | Attention at follow-up |
| 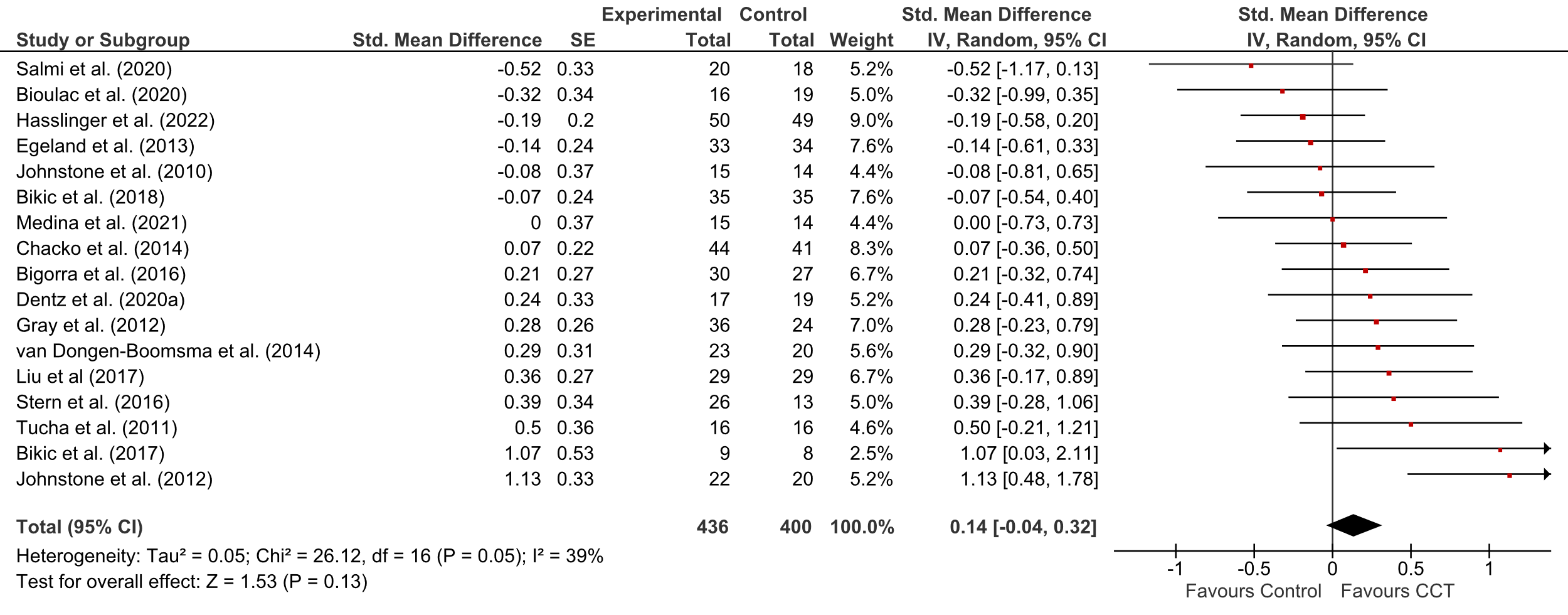 | 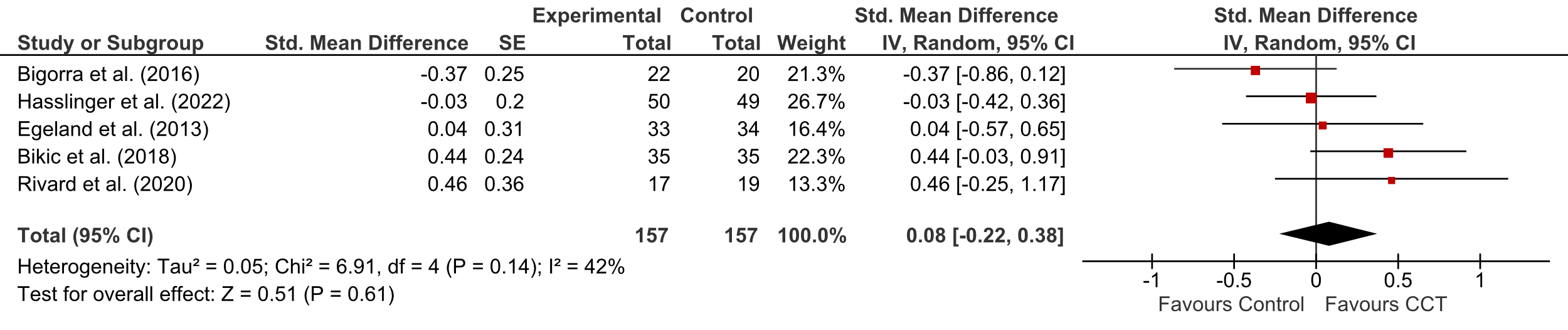 |
| Interference Inhibtion | Motor Inhibtion |
| 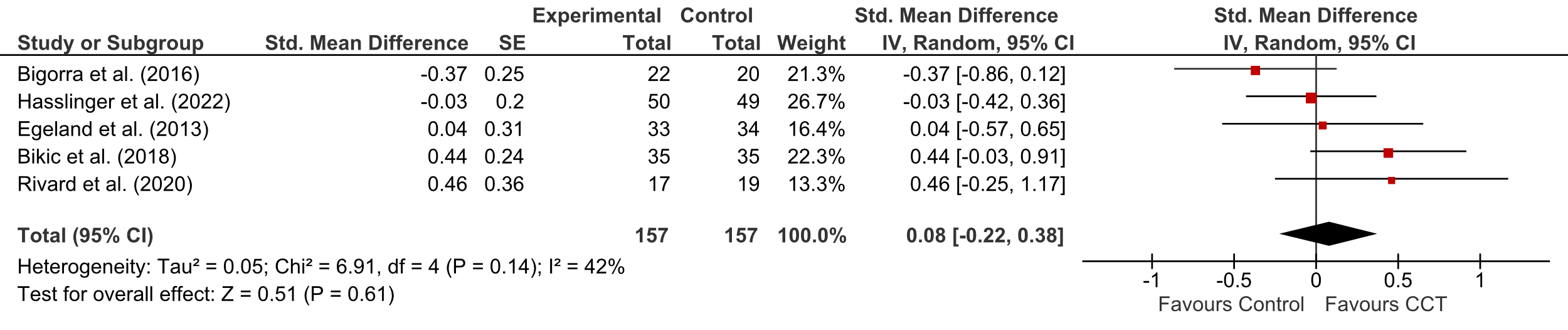 | 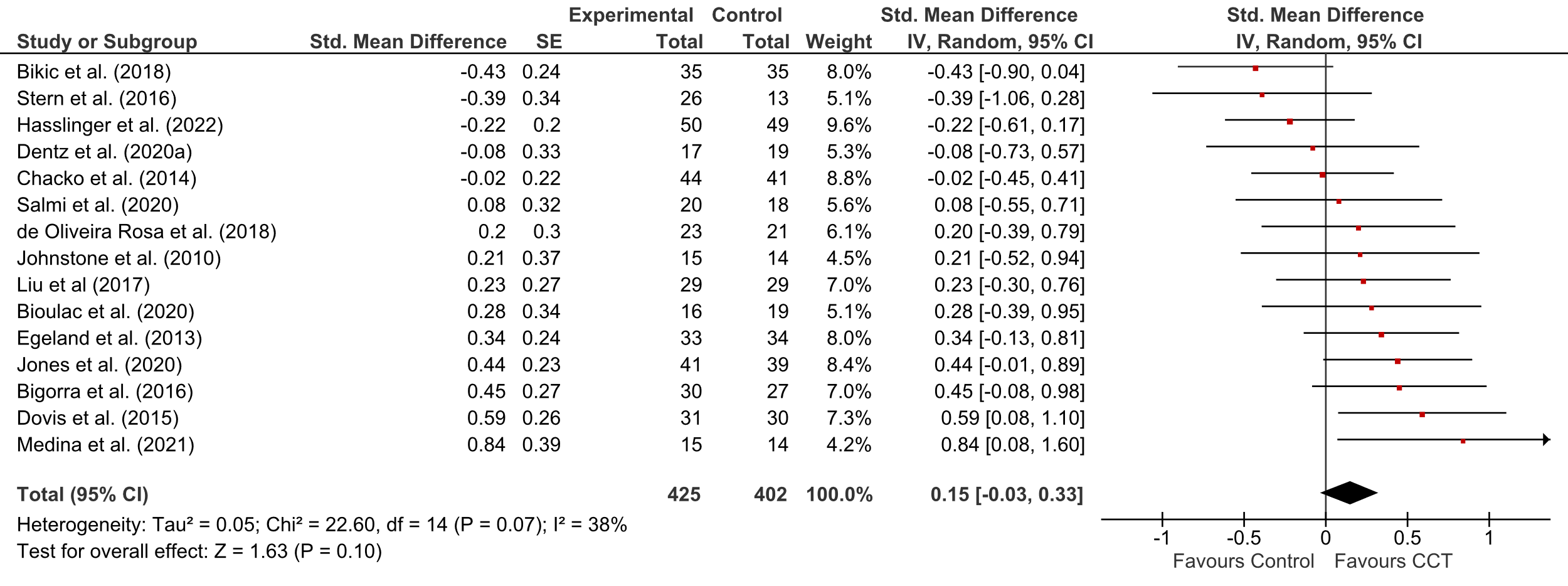 |
| Motor Inhibitition at follow-up | Non-Verbal Reasoning |
| 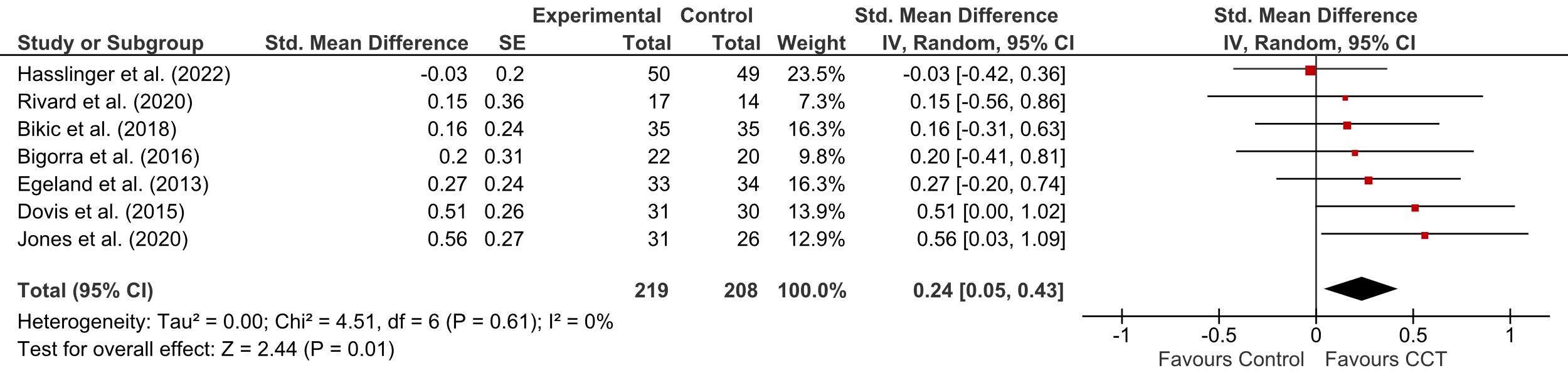 | 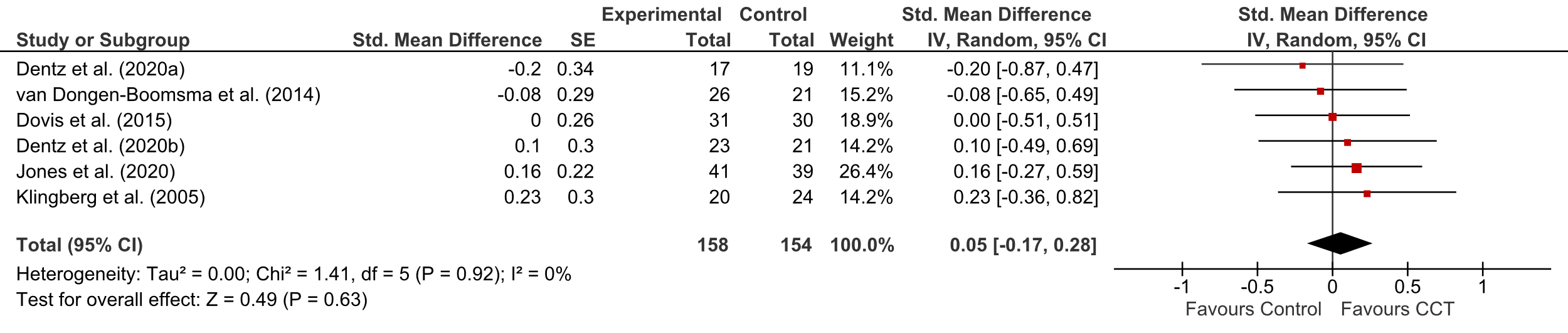 |
| Processing Speed | Set-Shifting |
| 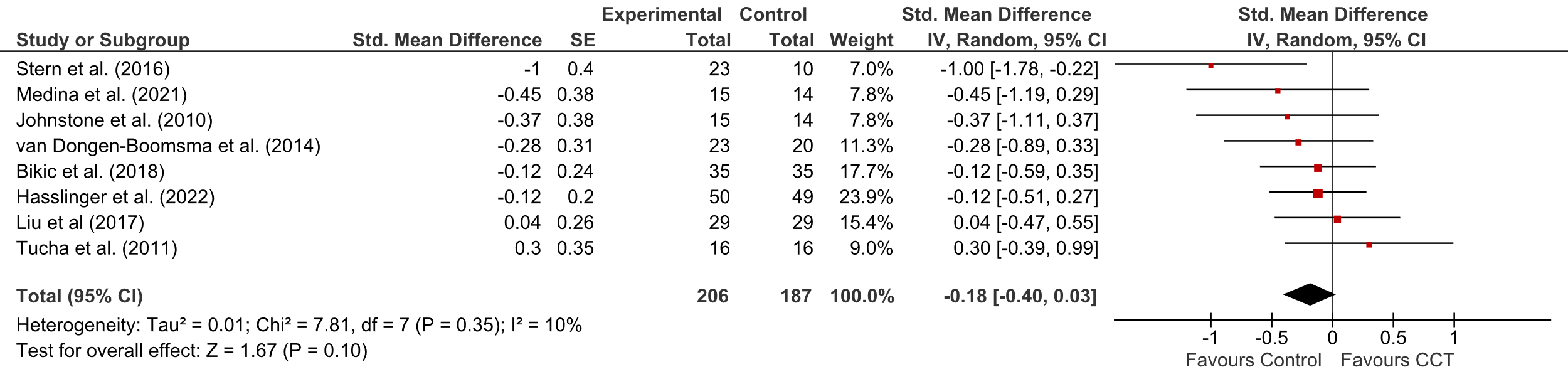 | 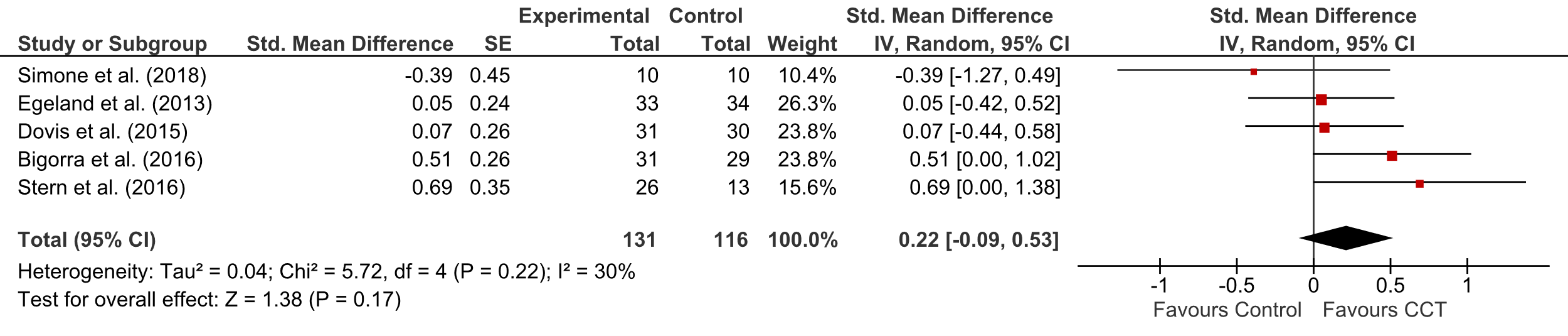 |
| Verbal WM at Follow-Up |  |
| 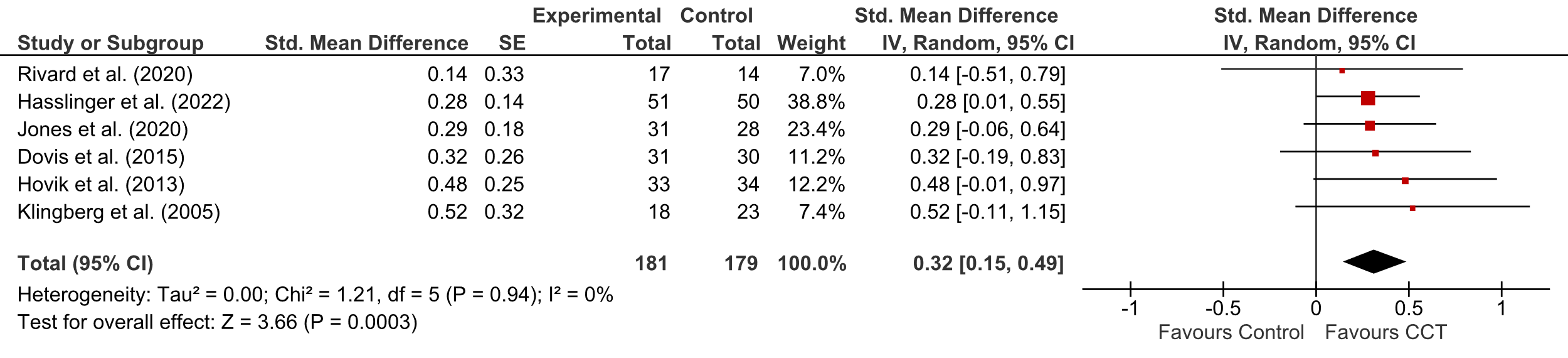 |  |
| Supplementary Figure 5. Forest plots for meta-analysis of effects MPROX and laboratory measures of neuropsychological processes. Note. Std. Mean Difference = Standardised Mean Difference with small sample adjustment | |

| **Academic Outcomes** | |
| --- | --- |
| Arithmetic Ability | Reading Comprehension |
| 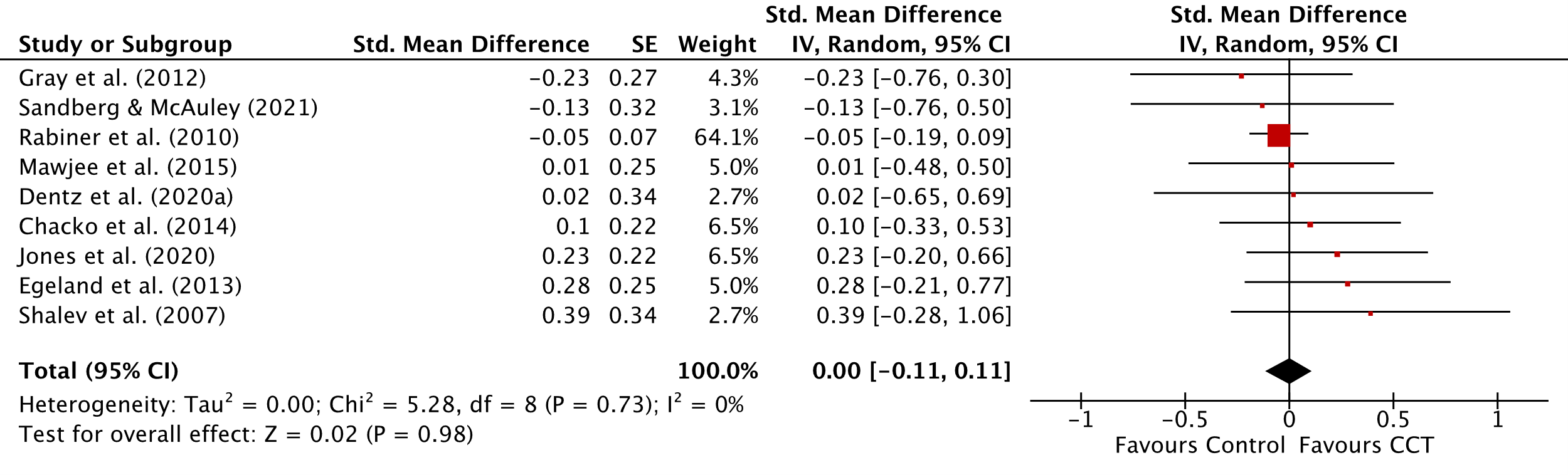 | 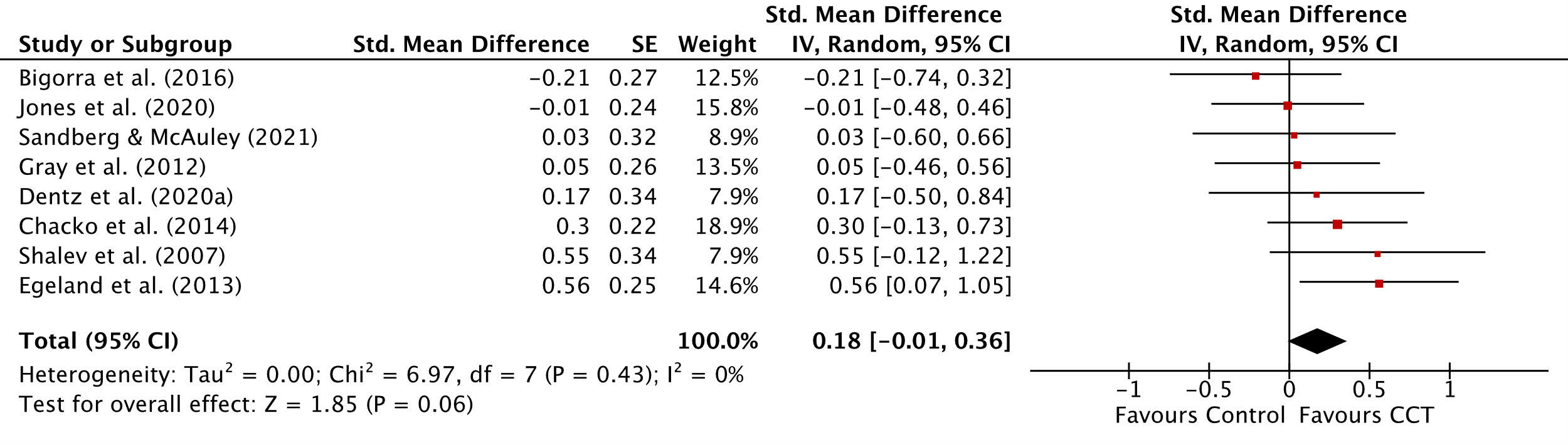 |
| Reading Comprehension – Follow-Up | Reading Fluency |
| 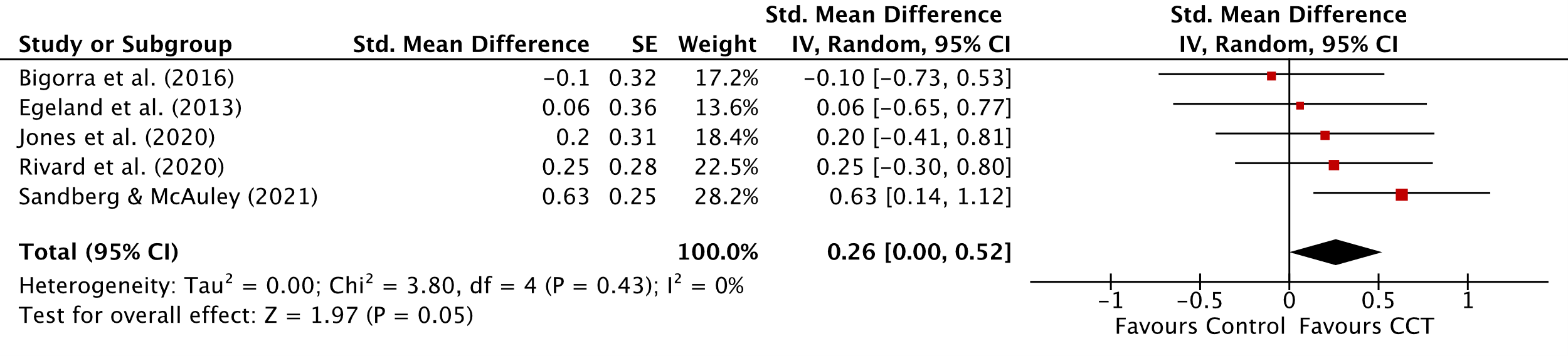 | **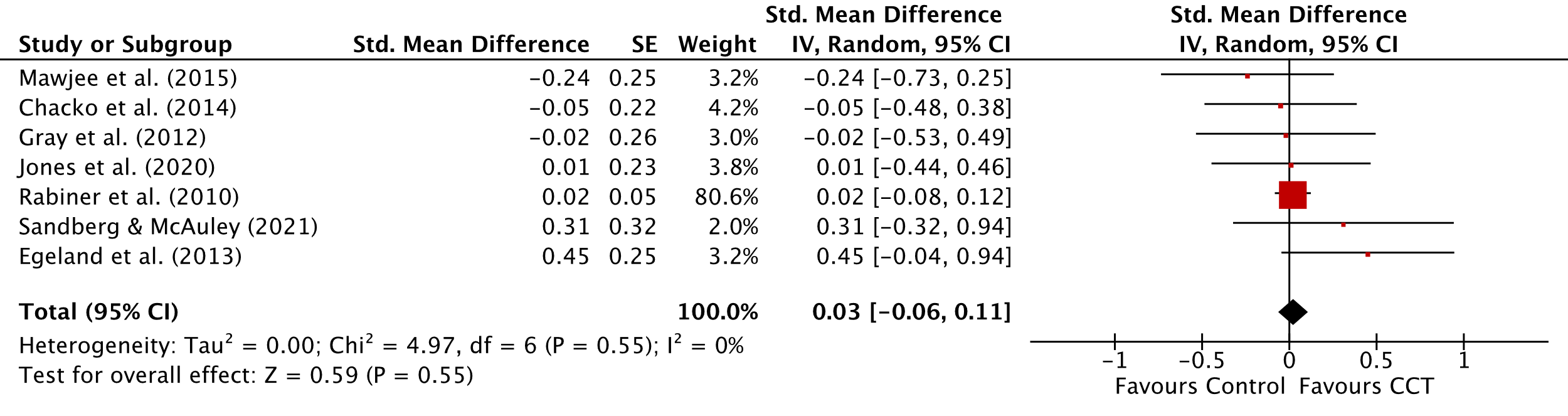** |
| Supplementary Figure 6. Forest plots for meta-analysis of effects academic outcomes. Note. Std. Mean Difference = Standardised Mean Difference with small sample adjustment | |

| **Supplementary Table 8. All neurocognitive outcome measures and the corresponding dependent variable included in meta-analyses grouped by cognitive domain** | | |
| --- | --- | --- |
| **Author** | **Outcome** | **Dependent Variable** |
| **Attention Outcomes** | | |
| Bigorra et al. (2016) | Conners' CPT-II | Omissions |
| Bikic et al. (2017) | CANTAB-Rapid Visual Information Processing | Probability of hit |
| Bikic et al. (2018) | CANTAB-Rapid Visual Information Processing | Probability of hit |
| Bioulac et al. (2020) | Conners' CPT-II | Omissions |
| Chacko et al. (2014) | A-X CPT | Omissions |
| Dentz et al. (2020a) | Conners' CPT-II -II | Omissions |
| Egeland et al. (2013) | Conners' CPT-II -II | Omissions |
| Gray et al. (2012) | D2 Test of Attention | Total Errors (Oms&Coms) |
| Hasslinger et al. (2022) | Conners' CPT-II -II | Omissions |
| Johnstone et al. (2010) | GNG Task | Omissions |
| Johnstone et al. (2012) | Oddball | % Target Correct |
| Liu et al. (2017) | GNG Task | Go Acc |
| Medina et al. (2021) | CPT | Omissions |
| Salmi et al. (2020) | CPT | Omissions |
| Stern et al. (2016) | GNG Task | Omissions |
| Tucha et al. (2011) | TAP Battery Vigilance Task | Omissions |
| van Dongen-Boomsma et al. (2014) | SA-DOTS-02K | RTV controlling for no. of Hits |
|  |  |  |
| **Attention Outcomes at follow-up** | | |
| Bigorra et al. (2016) | Conners' CPT-II | Omissions |
| Bikic et al. (2018) | CANTAB-Rapid Visual Information Processing | Probability of hit |
| Bikic et al. (2018) | CANTAB-Rapid Visual Information Processing | Probability of hit |
| Egeland et al. (2013) | Conners' CPT-II | Omissions |
| Hasslinger et al. (2022) | Conners' CPT-II | Omissions |
| Rivard et al. (2020) | Conners' CPT-II | Omissions |
| **Interference Inhibition** | | |
| de Oliveira Rosa et al. (2018) | NIH-Flanker | Incongruent Trials RTs |
| Dotare et al. (2020) | Attentional Network Task (aka Flanker Task) | Flanker RT Effect (No-cue Incongruent-Congruent) |
| Dovis et al. (2015) | Stroop Colour & Word Test | Stroop RT Effect |
| Egeland et al. (2013) | D-KEFS Color Word Interference Condition Trials | CW Interference Time |
| Johnstone et al. (2012) | Flanker Task | Incongruent Trial RTs |
| Klingberg et al. (2005) | Stroop Task Incongruent Trials | Accuracy |
| Medina et al. (2021) | Stroop Task | Congruent-Incongruent |
| van Dongen-Boomsma et al. (2014) | Day-Night Stroop task | Completion Time (Congruent-Incongruent controlling for correct responses) |
| **Motor Inhibition** | | |
| Bigorra et al. (2016) | Conners' CPT-II | Commissions |
| Bikic et al. (2018) | CANTAB Stop Task | SSRT last half |
| Bioulac et al. (2020) | CPT | Commissions |
| Chacko et al. (2014) | A-X CPT | Commissions |
| de Oliveira Rosa et al. (2018) | NIH-GNG | Go Acc |
| Dentz et al. (2020a) | CPT | Commissions |
| Dovis et al. (2015) | Stop task | SSRT |
| Egeland et al. (2013) | Conners’ CPT-II | Commissions |
| Hasslinger et al. (2022) | CPT-II | Commissions |
| Johnstone et al. (2010) | GNG | % No-Go Errs |
| Jones et al. (2020) | CPT | Commissions |
| Liu et al. (2017) | GNG | No-Go Acc |
| Medina et al. (2021) | CPT | Commissions |
| Salmi et al. (2020) | CPT | Commissions |
| Stern et al. (2016) | GNG | Commissions |
| **Motor Inhibition at follow-up** | | |
| Bigorra et al. (2016) | Conners' CPT-II | Commissions |
| Bikic et al. (2018) | CANTAB Stop Task | SSRT last half |
| Dovis et al. (2015) | Stop task | SSRT |
| Egeland et al. (2013) | Conners’ CPT-II | Commissions |
| Hasslinger et al. (2022) | CPT-II | Commissions |
| Jones et al. (2020) | CPT | Commissions |
| Rivard et al. (2020) | CPT | Commissions |
| **Non-Verbal Reasoning** | | |
| Dentz et al. (2020a) | Raven's progressive matrices | NR |
| Dentz et al. (2020b) | WAIS-III Matrix Reasoning subtest | NR |
| Dovis et al. (2015) | Raven coloured progressive matrices | Total Correct |
| Jones et al. (2020) | Ravens Standard Progressive Matrices | Total problems solved |
| Klingberg et al. (2005) | Raven's Matrix | Accuracy |
| van Dongen-Boomsma et al. (2014) | Shortened Raven Coloured Progressive Matrices | Total problems solved |
| **Processing Speed** | | |
| Bikic et al. (2018) | 5 Choice RTI | Reaction time (5-choice movement time) |
| Johnstone et al. (2010) | GNG | Go MRTs |
| Liu et al. (2017) | GNG | Go RTs |
| Medina et al. (2021) | CPT | MRTs |
| Stern et al. (2016) | IntegNeuro - Choice RT | MRT |
| Tucha et al. (2011) | Vigilance | MRT |
| van Dongen-Boomsma et al. (2014) | SA-DOTS-02K | Hit MRT controlling for N Hits |
| **Set-Shifting** | | |
| Bigorra et al. (2016) | TMT-Part B | NR |
| Dovis et al. (2015) | TMT | Difference between scaled score (non-switch trials [number-and letter sequencing) minus switch trials [number-letter switching]) |
| Egeland et al. (2013) | TMT (Task 4) | NR |
| Simone et al. (2018) | TMT-B | Time (seconds) |
| Stern et al. (2016) | IntegNeuro - Switching of Attention | Completion Time (digits + Letters) |
| **Verbal WM Tasks** | | |
| Dentz et al. (2020a) | WISC IV - Digit Span (Combined Forward/Backward) | Span Score |
| Dentz et al. (2020b) | WAIS-III Digit Span (Combined Forward/Backward) | Span Length |
| Dovis et al. (2015) | WISC-III Digit Span (Combined Forward/Backward) | Scaled Score |
| Gray et al. (2012) | WISC IV - Digit Span (Combined Forward/Backward) | Standard Score |
| Green et al. (2012) | WISC-IV Digit Span (Combined Forward/Backward) | Standard Score |
| Hasslinger et al. (2022) | WISC-IV/WAIS-IV Digit Span (Combined Forward/Backward) | Scale scores |
| Hovik et al. (2013) | WISC-IV Digit Span (Combined Forward/Backward) | Scale Scores ((Digit Span Forward + Digit Span Backward)/2) |
| Jaquerod 2020a | WAIS-IV Digit Span (Combined Forward/Backward) | normalized score |
| Jones et al. (2020) | WISC-IV Digit Span (Combined Forward/Backward) | Forward |
| Klingberg et al. (2005) | Digit Span (Combined Forward/Backward) | Max Recall |
| Medina et al. (2021) | WISC-IV Digit Span (Combined Forward/Backward) | Span |
| Salmi et al. (2020) | Digit Span (Forward & Backward) | Total Correct |
| Stern et al. (2016) | IntegNeuro - Digit Span (Combined Forward/Backward) | Recall Span |
| van Dongen-Boomsma et al. (2014) | Adapted WISC-III Digit Span (Combined Forward/Backward) | Total Correct |
| Woltering et al. (2019) | WAIS-IV Digit Span (Combined Forward/Backward) | Span Score |
| **Visuospatial WM** | | |
| Dentz et al. (2020a) | Wechsler Spatial Span (Combined Forward/Backward) | Span Score |
| Dentz et al. (2020b) | Corsi Block Tapping Task (Combined Forward/Backward) | Span Score |
| Dovis et al. (2015) | Corsi Block Tapping Task (Combined Forward/Backward) | Total Correct Score |
| Hasslinger et al. (2022) | Block Tapping (Combined Forward/Backward) | Raw scores |
| Klingberg et al. (2005) | Span-Board (Combined Forward/Backward) | Items |
| Mawjee et al. (2014) | CANTAB Spatial Span (Combined Forward/Backward) | Raw Score |
| Mawjee et al. (2015) | CANTAB Spatial Span (Combined Forward/Backward) | Raw Score |
| Medina et al. (2021) | Weschler Corsi Block Tapping (Combined Forward/Backward) | Span |
| van Dongen-Boomsma et al. (2014) | Knox Cubes LDT (Combined Forward/Backward) | Total Correct |
| Legend. Acc, accuracy; AWMA, Automated Working Memory Assessment; CANTAB, Cambridge Neuropsychological Test Automated Battery; CPT, Continuous Performance Task; D-KEFS, Delis–Kaplan Executive Function System; GNG, Go/No-Go; NIH, National Institute of Health; NR, not reported; RTs, mean reaction time; SSRT, stop-signal reaction time; TAP, Test of Attentional Performance Battery; TMT, trail making task; WAIS-III, Wechsler Adult Intelligence Scale 3rd Edition; WISC-IV, Wechsler Intelligence Scale for Children | | |

| **Supplementary Table 9. All academic outcome measures and the corresponding dependent variable included in meta-analyses grouped by academic domain** | | |
| --- | --- | --- |
| **Author** | **Outcome** | **Dependent Variable** |
| **Arithmetic Outcomes** | | |
| Chacko et al. (2014) | WRAT-Math Computation | Standardised Score |
| Dentz et al. (2020a) | WIAT-II Mathematical Reasoning | NR |
| Egeland et al. (2013) | Key Math | composite of Mental computation and Problem-Solving |
| Gray et al. (2012) | WRAT-Math Computation | NR |
| Jones et al. (2020) | WJ-III Math Applied Problem-Solving | NR |
| Mawjee et al. (2015) | Woodcock Johnson Math Fluency | NR |
| Rabiner et al. (2010) | WJ-III Math (composite of Calculation & Math Fluency) | NR |
| Sandberg et al. (2021) | WIAT-III Math | Standard Scores (composite [math problem solving, numerical operations]) |
| Shalev et al. (2007) | Math (In-House) | n/r |
| **Reading Fluency Outcomes** | | |
| Chacko et al. (2014) | WRAT - Word Reading | Standardised Score |
| Egeland et al. (2013) | LOGOS Reading Fluency | % correct |
| Gray et al. (2012) | WRAT - Word Reading | NR |
| Jones et al. (2020) | Woodcock Johnson-III Reading Fluency | NR |
| Mawjee et al. (2015) | Test of Word Reading Efficiency | NR |
| Rabiner et al. (2010) | Woodcock Johnson-III Reading (composite of Letter-word identification & Word Attack) | NR |
| Sandberg & McAuley (2021) | WIAT - Reading Fluency | Standard Scores |
| **Reading Comprehension** | | |
| Bigorra et al. (2016) | Reading Comprehension Test | NR |
| Chacko et al. (2014) | WRAT Sentence Comprehension | Standardised Score |
| Dentz et al. (2020a) | WIAT-II Reading Comprehension | NR |
| Egeland et al. (2013) | LOGOS Word Decoding Quality | % Correct |
| Gray et al. (2012) | WRAT-Sentence Comprehension | NR |
| Jones et al. (2020) | Reading Comprehension | NR |
| Sandberg et al. (2021) | WIAT - Reading Comprehension | Standard Scores |
| Shalev et al. (2007) | Reading Comprehension | NR |
| Legend. WIAT-II, Wechsler Individual Achievement Test 2nd Edition; WJ-II, Woodcock Johnson Tests 2nd Edition; WRAT-4, Wide Range Achievement Test 4; NR, not reported | | |

| Supplementary Table 10. Results of the separate meta-regressions with % of participants medicated, mean age of participants, publication year, or risk of bias rating as a predictor. Significant results are bolded | | | | | | | | |
| --- | --- | --- | --- | --- | --- | --- | --- | --- |
| **Outcome Measure** | **Predictor** | **Study N** | **Coefficient** | **SE** | **Z** | **p** | **95%CI** | |
|  |  |  |  |  |  |  | **Lower** | **Upper** |
| Pblind ADHD | Mean Age | 13 | 0.03 | 0.05 | 0.62 | 0.53 | -0.07 | 0.14 |
|  | Publication Year | 14 | -0.01 | 0.02 | -0.49 | 0.63 | -0.04 | 0.02 |
|  | Risk of Bias | 14 | -0.02 | 0.14 | -0.14 | 0.89 | -0.30 | 0.26 |
| Pblind Inattention | Mean Age | 13 | 0.06 | 0.07 | 0.85 | 0.40 | -0.08 | 0.19 |
|  | Publication Year | 14 | 0.00 | 0.02 | -0.26 | 0.80 | -0.04 | 0.03 |
|  | Risk of Bias | 14 | 0.12 | 0.17 | 0.70 | 0.49 | -0.21 | 0.44 |
| Pblind Hyperactivity/ Impulsitivity | Mean Age | 13 | 0.03 | 0.07 | 0.40 | 0.69 | -0.11 | 0.17 |
|  | Publication Year | 14 | 0.00 | 0.02 | 0.09 | 0.93 | -0.03 | 0.04 |
|  | Risk of Bias | 14 | -0.08 | 0.17 | -0.49 | 0.62 | -0.41 | 0.24 |
| Mprox ADHD Total | Mean Age | 19 | -0.05 | 0.06 | -0.85 | 0.39 | -0.16 | 0.06 |
|  | Publication Year | 24 | -0.02 | 0.02 | -1.05 | 0.29 | -0.05 | 0.02 |
|  | Risk of Bias | 24 | 0.24 | 0.14 | 1.75 | 0.08 | -0.03 | 0.51 |
| Mprox Inattention | Mean Age | 20 | -0.02 | 0.04 | -0.45 | 0.65 | -0.10 | 0.07 |
|  | Publication Year | 21 | -0.02 | 0.02 | -1.20 | 0.23 | -0.05 | 0.01 |
|  | Risk of Bias | 21 | 0.23 | 0.14 | 1.64 | 0.10 | -0.04 | 0.50 |
| Mprox Hyperactivity/ Impulsivity | Mean Age | 18 | 0.02 | 0.04 | 0.47 | 0.64 | -0.06 | 0.10 |
|  | Publication Year | 19 | 0.00 | 0.01 | -0.30 | 0.77 | -0.03 | 0.02 |
|  | Risk of Bias | 19 | 0.13 | 0.13 | 1.01 | 0.31 | -0.13 | 0.39 |
| BRIEF-GEC | Mean Age | 12 | 0.05 | 0.06 | 0.83 | 0.41 | -0.07 | 0.16 |
|  | Publication Year | 13 | -0.01 | 0.02 | -0.47 | 0.64 | -0.05 | 0.03 |
|  | Risk of Bias | 13 | 0.10 | 0.07 | 1.33 | 0.18 | -0.05 | 0.25 |
| Attention | Mean Age | 14 | 0.05 | 0.05 | 1.07 | 0.29 | -0.04 | 0.15 |
|  | Publication Year | 17 | -0.05 | 0.02 | -2.20 | **0.03** | **-0.09** | **-0.01** |
|  | Risk of Bias | 17 | 0.07 | 0.20 | 0.35 | 0.72 | -0.32 | 0.46 |
| Motor Inhibition | Mean Age | 12 | -0.03 | 0.10 | -0.31 | 0.76 | -0.23 | 0.17 |
|  | Publication Year | 15 | -0.02 | 0.03 | -0.51 | 0.61 | -0.07 | 0.04 |
|  | Risk of Bias | 15 | -0.29 | 0.19 | -1.53 | 0.13 | -0.65 | 0.08 |
| Verbal WM | Mean Age | 10 | -0.02 | 0.06 | -0.39 | 0.69 | -0.14 | 0.09 |
|  | Publication Year | 15 | -0.02 | 0.02 | -0.86 | 0.39 | -0.05 | 0.02 |
|  | Risk of Bias | 15 | 0.11 | 0.19 | 0.58 | 0.57 | -0.26 | 0.48 |
| Please note. A meta-regression was only carried out if there were at least 10 trials available. | | | | | | | | |

| **MPROX ADHD Total Symptoms** | **MPROX Inattention Symptoms** | **PBLIND Inattention Symptoms** | **BRIEF-GEC – Follow-Up** |
| --- | --- | --- | --- |
| 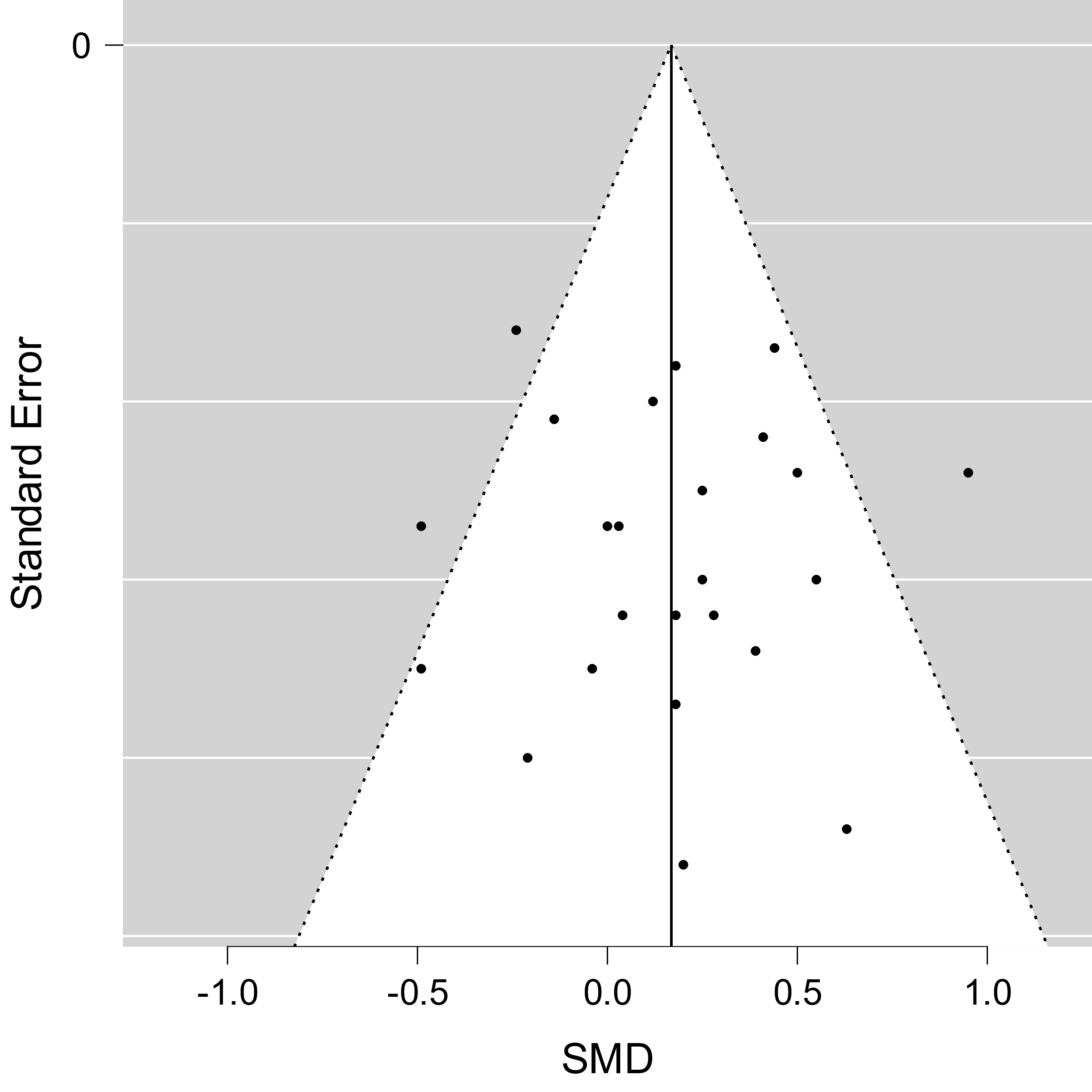 | 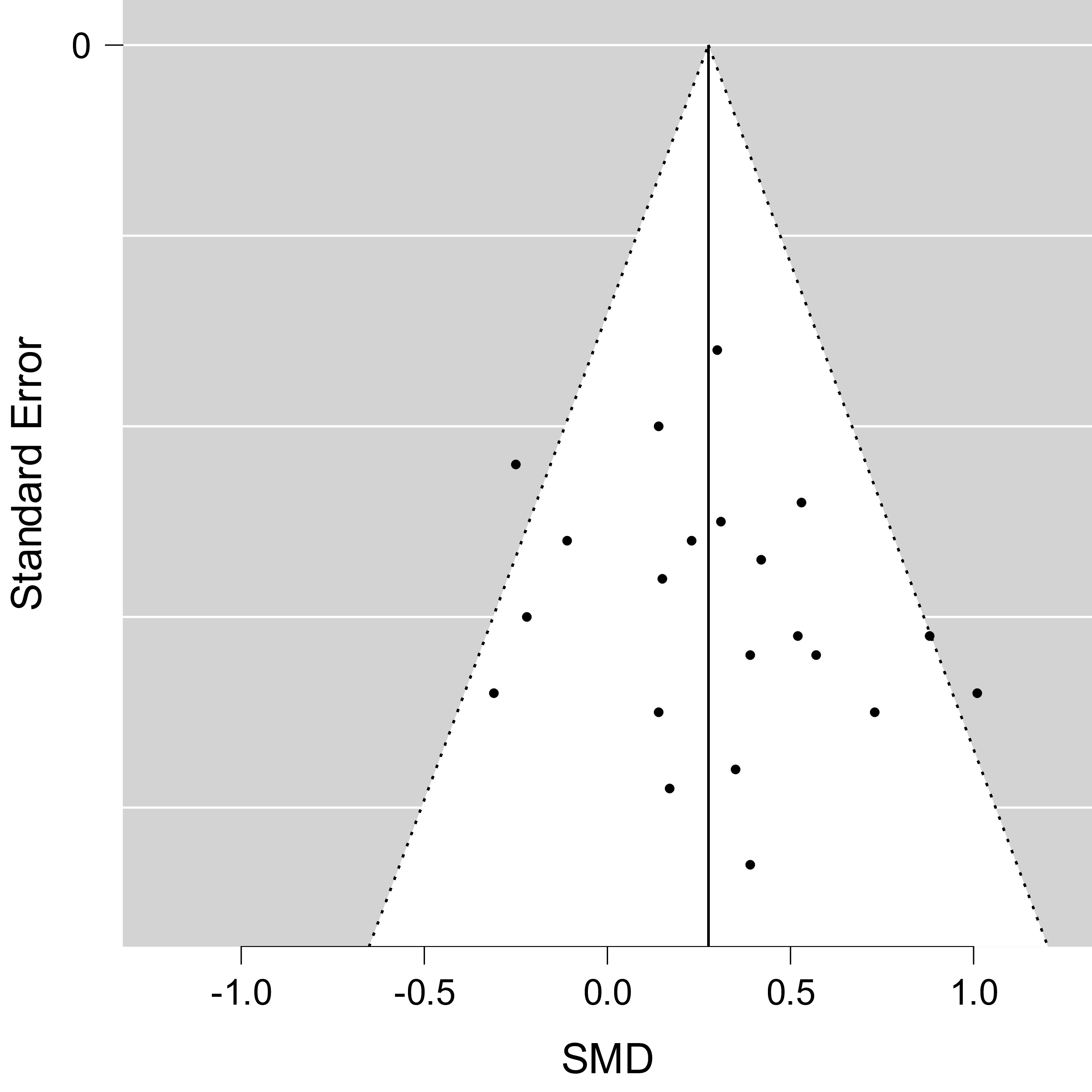 | 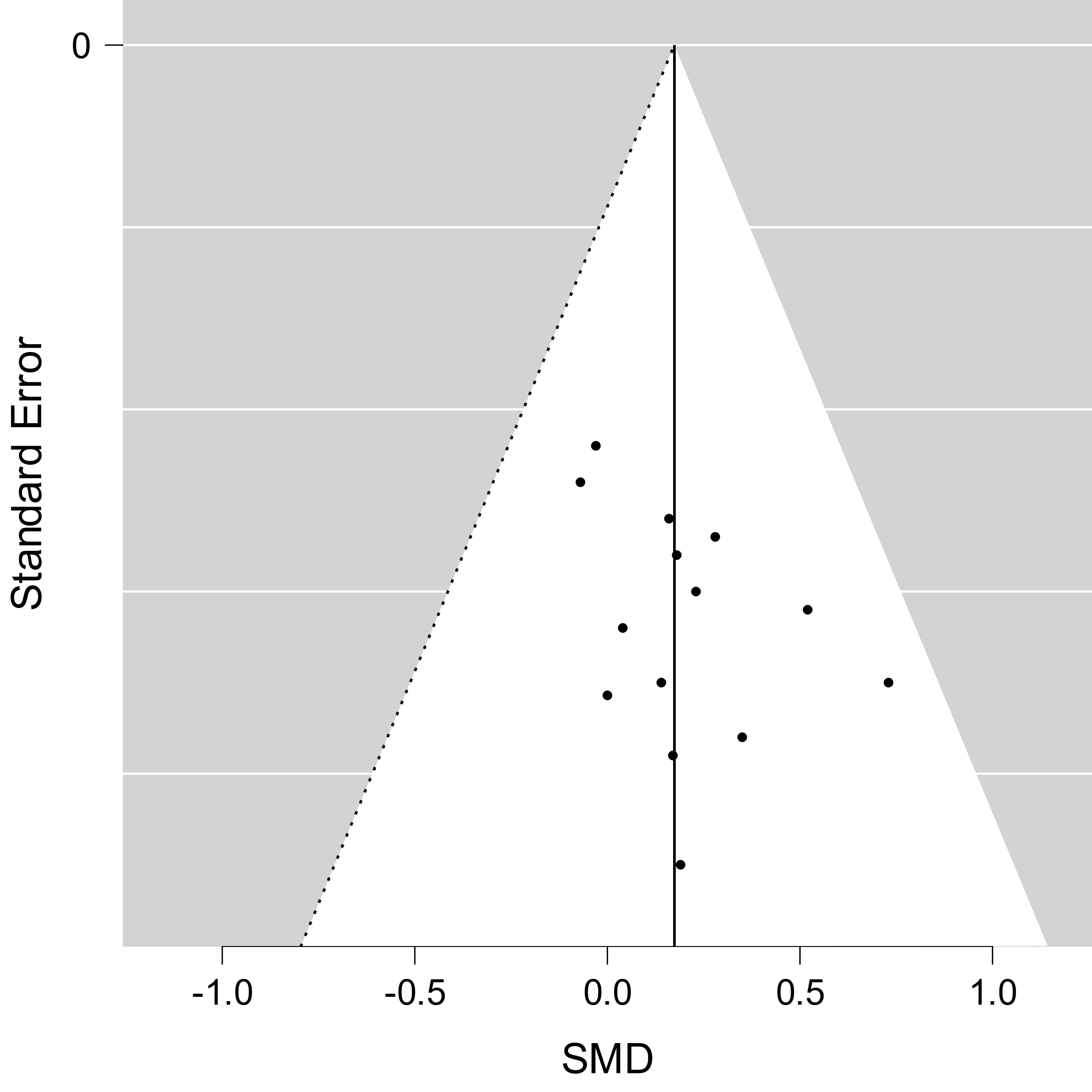 | 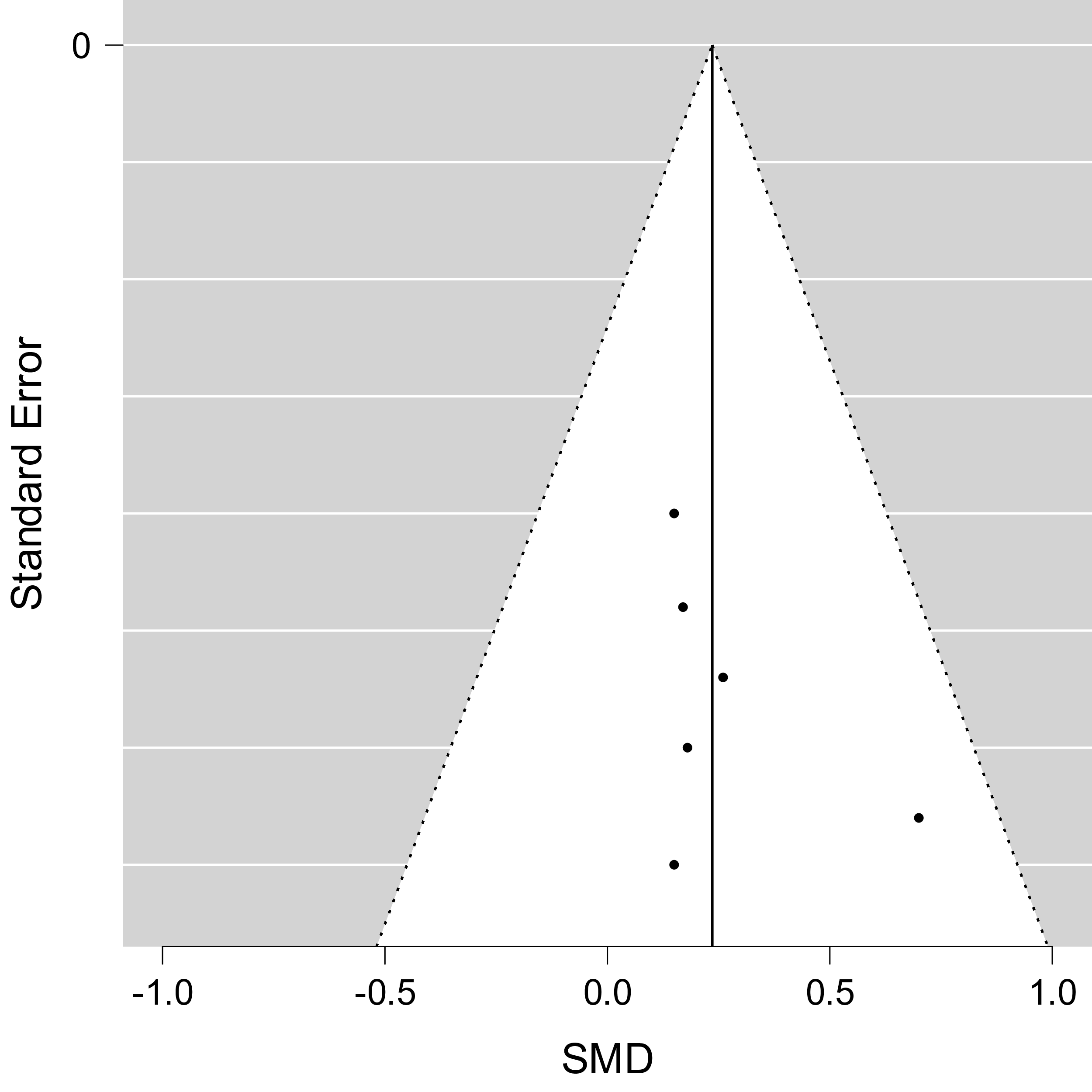 |
| \| **z** \| \| **p** \| \| \| --- \| --- \| --- \| --- \| \|  \| -0.074 \|  \| 0.941 \|  \| | \| **z** \| \| **p** \| \| \| --- \| --- \| --- \| --- \| \|  \| 1.059 \|  \| 0.289 \|  \| | \| **z** \| \| **p** \| \| \| --- \| --- \| --- \| --- \| \|  \| 1.125 \|  \| 0.261 \|  \| | \| **z** \| \| **p** \| \| \| --- \| --- \| --- \| --- \| \|  \| 0.767 \|  \| 0.443 \|  \| |
| **Motor inhibition – Follow-Up** | **Verbal WM** | **Verbal WM – Follow-Up** | **Visuospatial WM** |
| 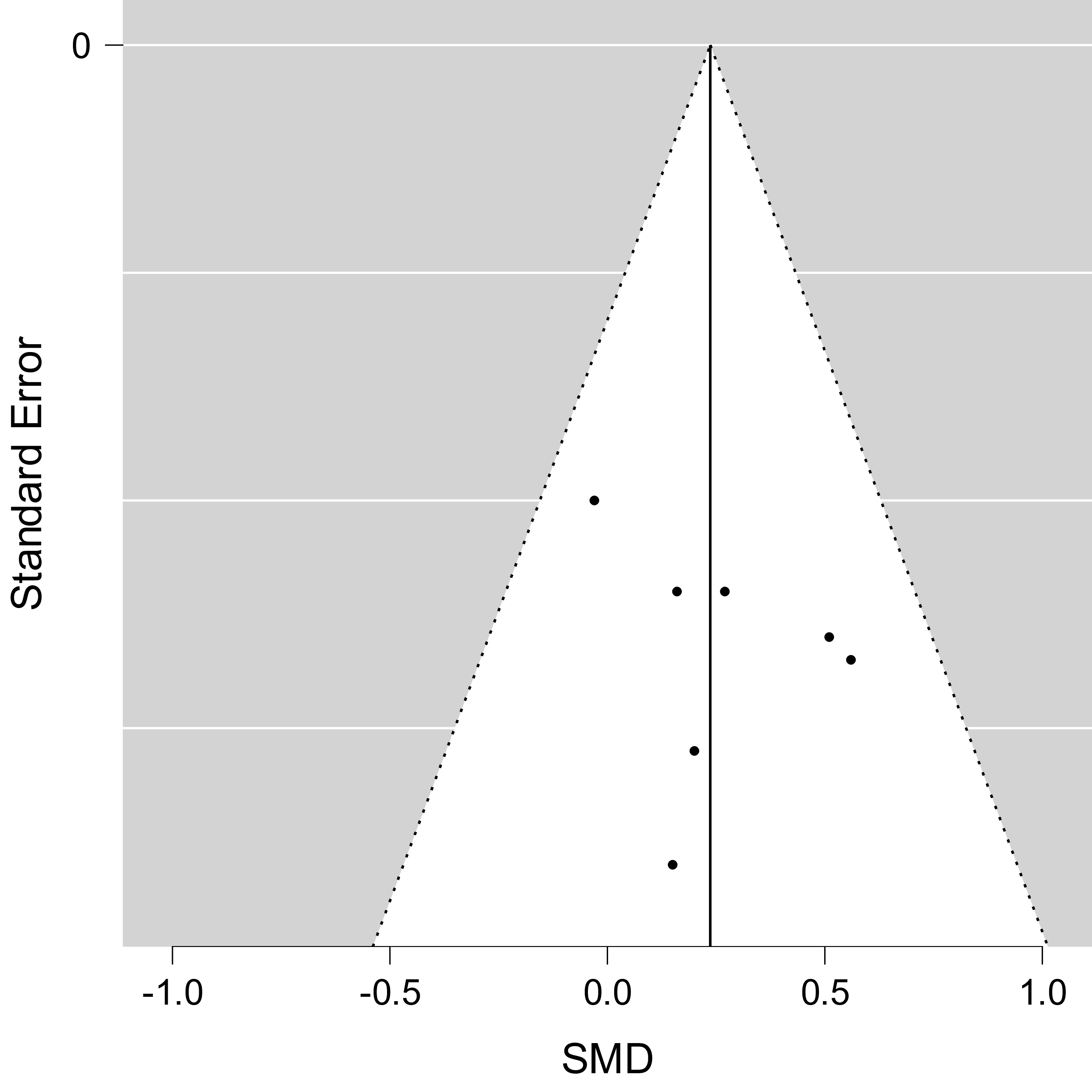 | 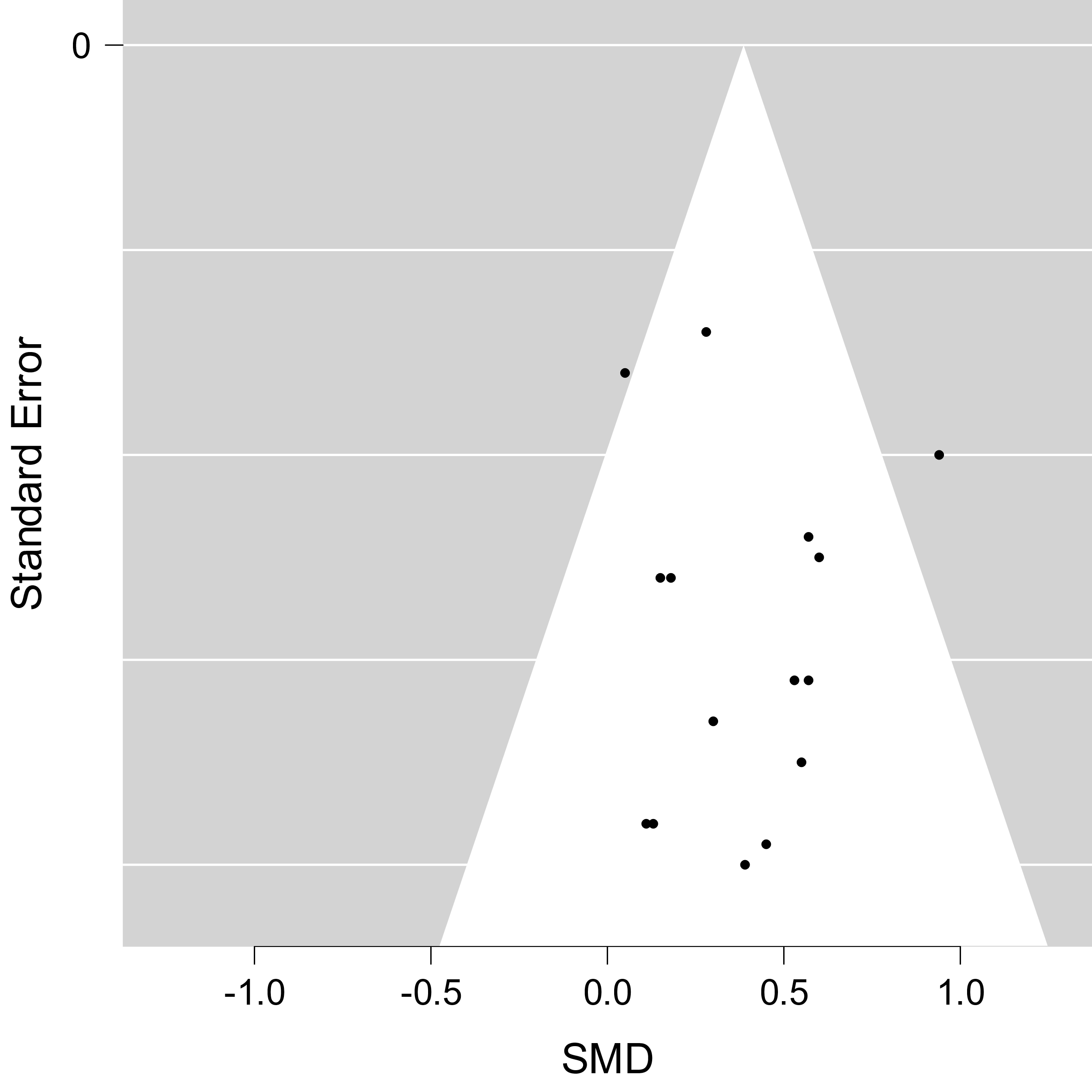 | 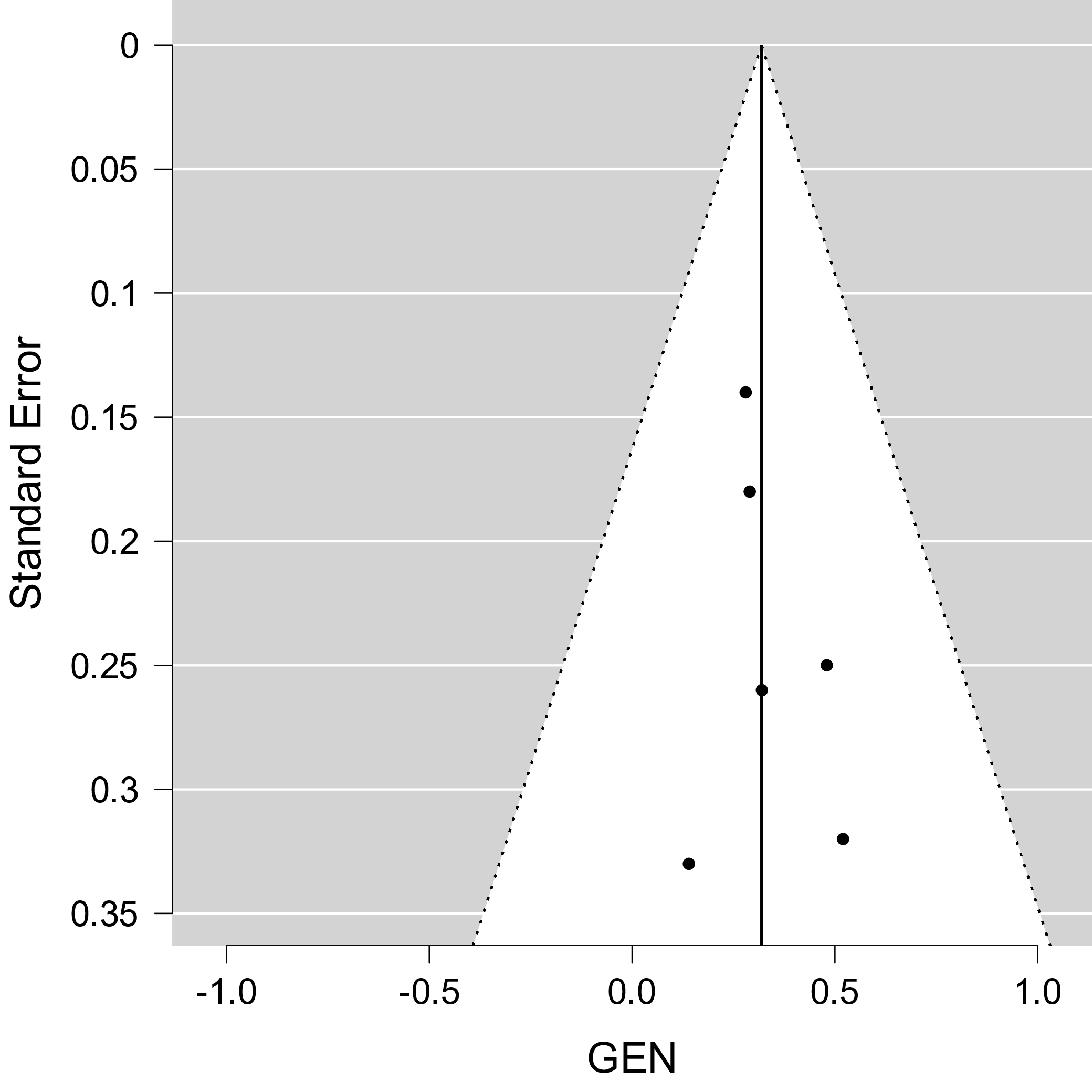 | 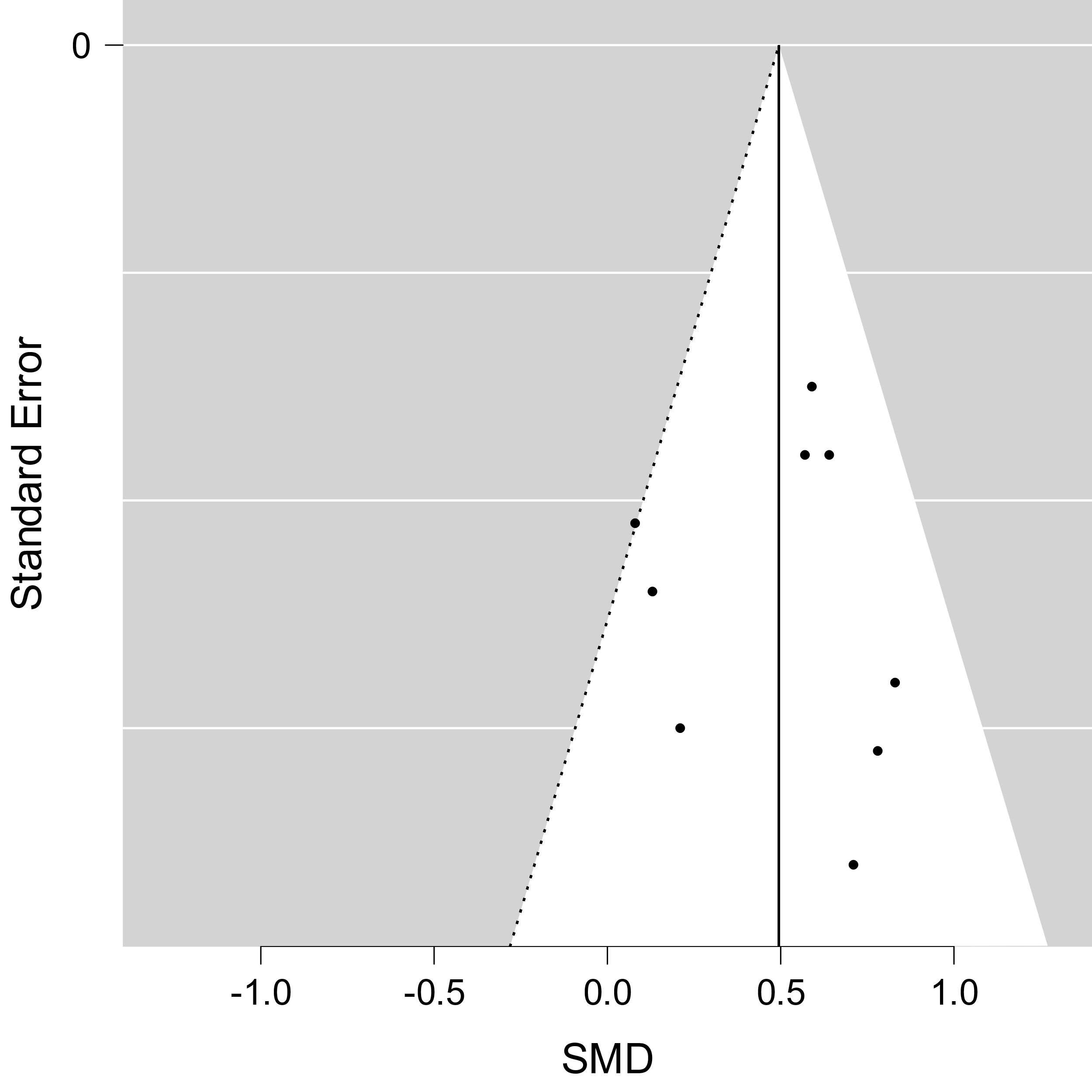 |
| \| **z** \| \| **p** \| \| \| --- \| --- \| --- \| --- \| \|  \| 0.829 \|  \| 0.407 \|  \| | \| **z** \| \| **p** \| \| \| --- \| --- \| --- \| --- \| \|  \| 0.162 \|  \| 0.872 \|  \| | \| **z** \| \| **p** \| \| \| --- \| --- \| --- \| --- \| \|  \| 0.067 \|  \| 1.00 \|  \| | \| **z** \| \| **p** \| \| \| --- \| --- \| --- \| --- \| \|  \| 0.139 \|  \| 0.890 \|  \| |
| **Reading Comprehension –**  **Follow-up** |  |  |  |
| 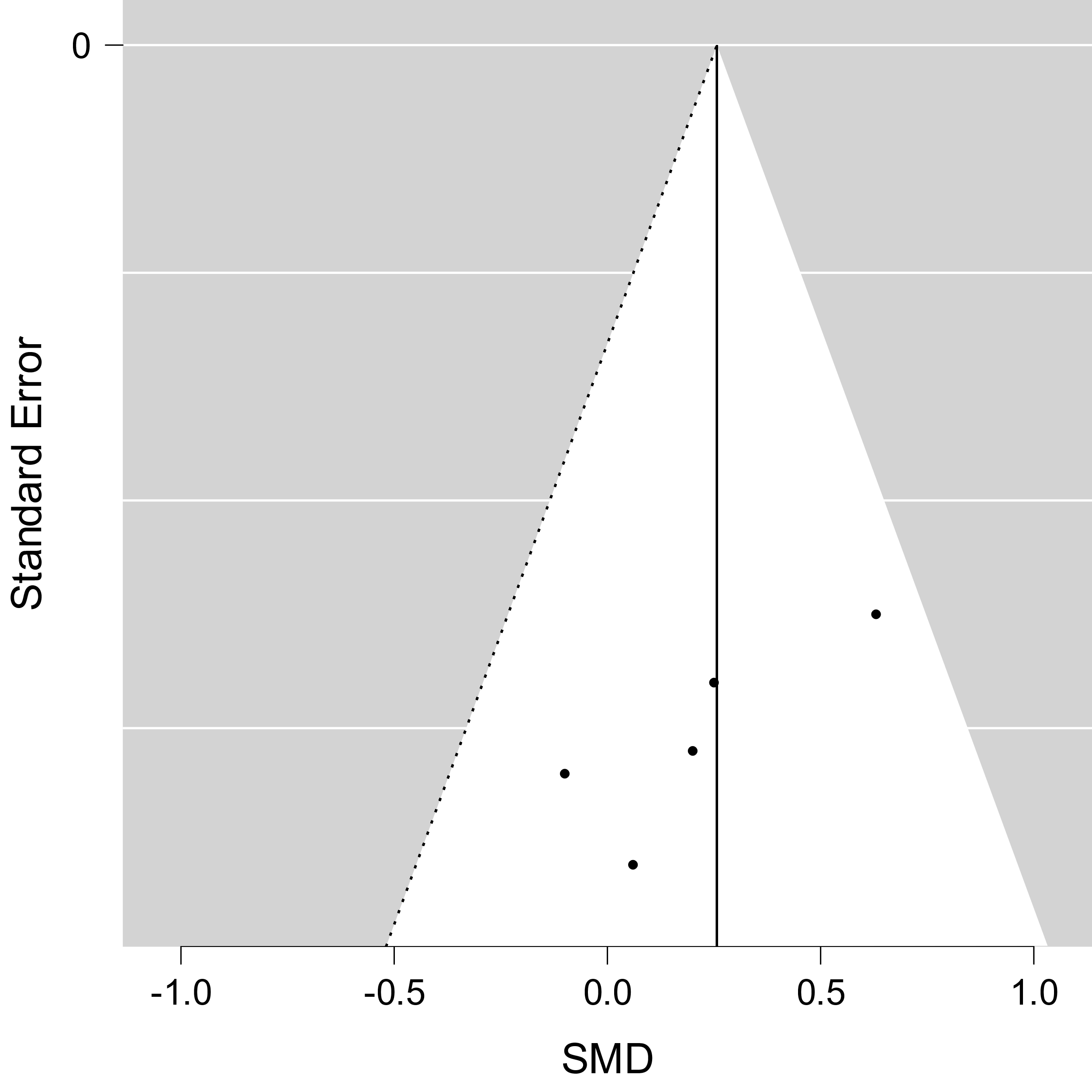 |  |  |  |
| \| **z** \| \| **p** \| \| \| --- \| --- \| --- \| --- \| \|  \| -1.673 \|  \| 0.094 \|  \| |  |  |  |
| *Supplementary Figure 7. Funnel plots and results from regression test for funnel plot asymmetry (i.e., Egger’s test) for meta-analyses that were significant will all trials only.* | | | |

| Supplementary Table 11. Summary of results showing pooled standardized mean differences (SMD; with Hedges’ *g* adjustment) between treatment and control arms for PBLIND measures of ADHD symptoms collected at the first assessment after the final CCT session. Significant values are bolded. | | | | | | | | | |
| --- | --- | --- | --- | --- | --- | --- | --- | --- | --- |
|  |  |  |  |  | **Effect Size Estimate** | | | **Heterogeneity** | |
| **Analysis** | **Outcome** | **Trials Included** | **Study N** | **Total N** | **SMD** | **95%CI** | ***p*** | **I^2^** | ***p**** |
| **Most proximal PBLIND outcomes where available**** | ADHD Total | All | 14 | 640 | 0.10 | -0.04 to 0.25 | 0.17 | 19 | 0.25 |
|  | Inattention | All | 14 | 636 | 0.30 | 0.13 to 0.48 | <0.001 | 23 | 0.21 |
|  | Hyperactivity/ Impulsivity | All | 14 | 637 | 0.05 | -0.11 to 0.20 | 0.55 | 0 | 0.90 |
| **Including Kofler et al. (2020)** | MPROX - ADHD Total | All | 25 | 1187 | **0.17** | **0.04 to 0.31** | **0.01** | **40** | **0.02** |
|  | MPROX - Inattention | All | 22 | 1115 | **0.28** | **0.14 to 0.41** | **<0.001** | 25 | 0.14 |
|  | MPROX - Hyperactivity/ Impulsivity | All | 20 | 991 | 0.09 | -0.04 to 0.22 | 0.16 | 0 | 0.75 |
|  | PBLIND - ADHD Total | All | 15 | 685 | 0.13 | 0.01 to 0.25 | 0.03 | 0 | 0.93 |
|  | PBLIND - Inattention | All | 15 | 685 | 0.18 | 0.03 | 0.34 | 0 | 0.94 |
|  | PBLIND - Hyperactivity/ Impulsivity | All | 15 | 685 | 0.12 | -0.03 to 0.27 | 0.12 | 0 | 0.99 |
|  | Motor Inhibition | All | 16 | 881 | 0.11 | -0.08 to 0.29 | 0.27 | 48 | 0.02 |
| *p-values from Q – i.e., the chi-squared test statistic; **For this analysis, where multiple PBLIND assessments were available, we chose the one most proximal to the intervention delivery – as per the request of the reviewer – rather than the one most distal – as per our pre-specified protocol. ADHD, attention-deficit/hyperactivity disorder; CI, Confidence Intervals; I^2^, percentage of between-study variation across SMDs that is due to heterogeneity rather than chance; N, sample size; SMD, Hedges’ *g*; | | | | | | | | | |

**REFERENCES**

1. Cortese S, Ferrin M, Brandeis D, Buitelaar J, Daley D, Dittmann RW, et al. Cognitive Training for Attention-Deficit/Hyperactivity Disorder: Meta-Analysis of Clinical and Neuropsychological Outcomes From Randomized Controlled Trials. Journal of the American Academy of Child & Adolescent Psychiatry. 2015 Mar;54(3):164–74.

2. Johnstone SJ, Roodenrys S, Blackman R, Johnston E, Loveday K, Mantz S, et al. Neurocognitive training for children with and without AD/HD. Atten Defic Hyperact Disord. 2012;4(1):11–23.

3. Johnstone SJ, Roodenrys S, Phillips E, Watt AJ, Mantz S. A pilot study of combined working memory and inhibition training for children with AD/HD. ADHD Atten Def Hyp Disord. 2010 Mar;2(1):31–42.

4. Rabiner DL, Murray DW, Skinner AT, Malone PS. A Randomized Trial of Two Promising Computer-Based Interventions for Students with Attention Difficulties. J Abnorm Child Psychol. 2010 Jan;38(1):131–42.

5. Steiner NJ, Frenette EC, Rene KM, Brennan RT, Perrin EC. Neurofeedback and cognitive attention training for children with attention-deficit hyperactivity disorder in schools. Journal of developmental and behavioral pediatrics : JDBP. 2014;35(1):18–27.

6. van der Oord S, Ponsioen AJGB, Geurts HM, Ten Brink EL, Prins PJM. A pilot study of the efficacy of a computerized executive functioning remediation training with game elements for children with ADHD in an outpatient setting: outcome on parent- and teacher-rated executive functioning and ADHD behavior. J Atten Disord. 2014 Nov;18(8):699–712.

7. Tamm L, Epstein JN, Peugh JL, Nakonezny PA, Hughes CW. Preliminary data suggesting the efficacy of attention training for school-aged children with ADHD. Developmental cognitive neuroscience. 2013;4:16–28.

8. Virta. Short cognitive behavioral therapy and cognitive training for adults with ADHD &ndash; a randomized controlled pilot study. NDT. 2010 Jul;443.

9. Kollins SH, Cutler AJ, Khattak S, Weiss MD, Donnelly G, Reiz SJL. A randomized double-blind placebocontrolled multicenter study measuring the efficacy and safety of a novel, extended-release formulation of methylphenidate (prc-063) in adolescents with attention-deficit/hyperactivity disorder. Journal of the American Academy of Child and Adolescent Psychiatry. 2016;55(10 Supplement 1):S217–8.

10. Gray SA, Chaban P, Martinussen R, Goldberg R, Gotlieb H, Kronitz R, et al. Effects of a computerized working memory training program on working memory, attention, and academics in adolescents with severe LD and comorbid ADHD: a randomized controlled trial. Journal of child psychology and psychiatry, and allied disciplines. 2012;53(12):1277–84.

11. Hautmann C, Doepfner M, Katzmann J, Schuermann S, Metternich-Kaizman TW, Jaite C, et al. Sequential treatment of ADHD in mother and child (AIMAC study): importance of the treatment phases for intervention success in a randomized trial. BMC PSYCHIATRY. 2018 Dec 13;18.

12. Hasslinger J, Bölte S, Jonsson U. Slow Cortical Potential Versus Live Z-score Neurofeedback in Children and Adolescents with ADHD: A Multi-arm Pragmatic Randomized Controlled Trial with Active and Passive Comparators. Res Child Adolesc Psychopathol [Internet]. 2021 Sep 3 [cited 2022 Mar 15]; Available from: https://link.springer.com/10.1007/s10802-021-00858-1

13. Bigorra A, Garolera M, Guijarro S, Hervás A. Long-term far-transfer effects of working memory training in children with ADHD: a randomized controlled trial. Eur Child Adolesc Psychiatry. 2016 Aug;25(8):853–67.

14. Bikic A, Christensen TØ, Leckman JF, Bilenberg N, Dalsgaard S. A double-blind randomized pilot trial comparing computerized cognitive exercises to Tetris in adolescents with attention-deficit/hyperactivity disorder. Nord J Psychiatry. 2017 Aug;71(6):455–64.

15. Bikic A, Leckman JF, Christensen TØ, Bilenberg N, Dalsgaard S. Attention and executive functions computer training for attention-deficit/hyperactivity disorder (ADHD): results from a randomized, controlled trial. European Child & Adolescent Psychiatry. 2018 Dec;27(12):1563–74.

16. Bioulac S, Micoulaud-Franchi JA, Maire J, Bouvard MP, Rizzo AA, Sagaspe P, et al. Virtual Remediation Versus Methylphenidate to Improve Distractibility in Children With ADHD: A Controlled Randomized Clinical Trial Study. J Atten Disord. 2020 Jan;24(2):326–35.

17. Chacko A, Bedard AC, Marks DJ, Feirsen N, Uderman JZ, Chimiklis A, et al. A randomized clinical trial of Cogmed Working Memory Training in school-age children with ADHD: a replication in a diverse sample using a control condition. JOURNAL OF CHILD PSYCHOLOGY AND PSYCHIATRY. 2014 Mar;55(3):247–55.

18. Dentz A, Guay MC, Gauthier B, Romo L, Parent V. Is the Cogmed program effective for youths with attention deficit/hyperactivity disorder under pharmacological treatment? Applied Cognitive Psychology. 2020;34(3):577–89.

19. Dovis S, Van der Oord S, Wiers RW, Prins PJM. Improving Executive Functioning in Children with ADHD: Training Multiple Executive Functions within the Context of a Computer Game. A Randomized Double-Blind Placebo Controlled Trial. PLOS ONE. 2015;10(4):1–30.

20. de Oliveira Rosa V, Moreira-Maia CR, Wagner F, Simioni A, de Fraga Bassotto C, Moritz GR, et al. Computerized Cognitive Training for ADHD as an Add-On Treatment to Stimulants: A Randomized Clinical Trial. J Atten Disord. 2018 Dec 14;1087054718816818.

21. Egeland J., Aarlien A.K., Saunes B.-K. Few Effects of Far Transfer of Working Memory Training in ADHD: A Randomized Controlled Trial. PLoS ONE. 2013;8(10):e75660.

22. Green CT, Long DL, Green D, Iosif AM, Dixon JF, Miller MR, et al. Will Working Memory Training Generalize to Improve Off-Task Behavior in Children with Attention-Deficit/Hyperactivity Disorder? Neurotherapeutics. 2012 Jul;9(3):639–48.

23. Hasslinger J, Jonsson U, Bölte S. Immediate and Sustained Effects of Neurofeedback and Working Memory Training on Cognitive Functions in Children and Adolescents with ADHD: A Multi-Arm Pragmatic Randomized Controlled Trial. Journal of Attention Disorders. 2021;15.

24. Hasslinger J, Bölte S, Jonsson U. Slow Cortical Potential Versus Live Z-score Neurofeedback in Children and Adolescents with ADHD: A Multi-arm Pragmatic Randomized Controlled Trial with Active and Passive Comparators. Res Child Adolesc Psychopathol. 2022 Apr;50(4):447–62.

25. Hovik K.T., Saunes B.-K., Aarlien A.K., Egeland J. RCT of working memory training in ADHD: Long-term near-transfer effects. PLoS ONE. 2013;8(12):e80561.

26. Johnstone SJ, Roodenrys S, Blackman R, Johnston E, Loveday K, Mantz S, et al. Neurocognitive training for children with and without AD/HD. ADHD Atten Def Hyp Disord. 2012 Mar;4(1):11–23.

27. Jones MR, Katz B, Buschkuehl M, Jaeggi SM, Shah P. Exploring N-Back Cognitive Training for Children With ADHD. J Atten Disord. 2020 Mar;24(5):704–19.

28. Klingberg T, Fernell E, Olesen PJ, Johnson M, Gustafsson P, Dahlström K, et al. Computerized Training of Working Memory in Children With ADHD-A Randomized, Controlled Trial. J AM ACAD CHILD ADOLESC PSYCHIATRY. 2005;10.

29. Kollins SH, DeLoss DJ, Cañadas E, Lutz J, Findling RL, Keefe RSE, et al. A novel digital intervention for actively reducing severity of paediatric ADHD (STARS-ADHD): a randomised controlled trial. The Lancet Digital Health. 2020 Apr;2(4):e168–78.

30. Medina R, Bouhaben J, de Ramón I, Cuesta P, Antón-Toro L, Pacios J, et al. Electrophysiological Brain Changes Associated With Cognitive Improvement in a Pediatric Attention Deficit Hyperactivity Disorder Digital Artificial Intelligence-Driven Intervention: Randomized Controlled Trial. J Med Internet Res. 2021 Nov 26;23(11):e25466.

31. Meyer KN, Santillana R, Miller B, Clapp W, Way M, Bridgman-Goines K, et al. Computer-based inhibitory control training in children with Attention-Deficit/Hyperactivity Disorder (ADHD): Evidence for behavioral and neural impact. PLOS ONE. 2020;25.

32. Rivard C, Dentz A, Romo L, Parent V, Guay MC, Gauthier B. Long term effects of working memory training (Cogmed) among children with ADHD. Neuropsychiatrie de l’Enfance et de l’Adolescence. 2020;68(1):29–38.

33. (Sol) Sandberg S, McAuley T. Hospital-Based Modified Cogmed Working Memory Training for Youth With ADHD. J Atten Disord. 2021 Dec 23;108705472110664.

34. Shalev L, Tsal Y, Mevorach C. Computerized Progressive Attentional Training (CPAT) Program: Effective Direct Intervention for Children with ADHD. Child Neuropsychology. 2007 Jun 11;13(4):382–8.

35. Simone M, Viterbo RG, Margari L, Iaffaldano P. Computer-assisted rehabilitation of attention in pediatric multiple sclerosis and ADHD patients: a pilot trial. BMC Neurol [Internet]. 2018 Jun 8 [cited 2019 Jun 18];18. Available from: https://www.ncbi.nlm.nih.gov/pmc/articles/PMC5992821/

36. Steiner NJ, Sheldrick RC, Gotthelf D, Perrin EC. Computer-Based Attention Training in the Schools for Children With Attention Deficit/Hyperactivity Disorder: A Preliminary Trial. Clin Pediatr (Phila). 2011 Jul;50(7):615–22.

37. Steiner NJ, Frenette EC, Rene KM, Brennan RT, Perrin EC. In-school neurofeedback training for ADHD: sustained improvements from a randomized control trial. Pediatrics. 2014;133(3):483–92.

38. Tucha O, Tucha L, Kaumann G, Konig S, Lange KM, Stasik D, et al. Training of attention functions in children with attention deficit hyperactivity disorder. Atten Defic Hyperact Disord. 2011;3(3):271–83.

39. van Dongen-Boomsma M, Vollebregt MA, Buitelaar JK, Slaats-Willemse D. Working memory training in young children with ADHD: a randomized placebo-controlled trial. Journal of Child Psychology and Psychiatry. 2014 Aug;55(8):886–96.

40. Dentz A, Guay MC, Parent V, Romo L. Working Memory Training for Adults With ADHD. J Atten Disord. 2020 Apr;24(6):918–27.

41. Dotare M, Bader M, Mesrobian SK, Asai Y, Villa AEP, Lintas A. Attention networks in adhd adults after working memory training with a dual n-back task. Brain Sciences. 2020;10(10):1–35.

42. Jaquerod ME, Mesrobian SK, Villa AEP, Bader M, Lintas A. Early Attentional Modulation by Working Memory Training in Young Adult ADHD Patients during a Risky Decision-Making Task. Brain Sciences. 2020 Jan 9;10(1):38.

43. Liu ZX, Glizer D, Tannock R, Woltering S. EEG alpha power during maintenance of information in working memory in adults with ADHD and its plasticity due to working memory training: A randomized controlled trial. Clin Neurophysiol. 2016 Feb;127(2):1307–20.

44. Liu ZX, Lishak V, Tannock R, Woltering S. Effects of working memory training on neural correlates of Go/Nogo response control in adults with ADHD: A randomized controlled trial. Neuropsychologia. 2017 Jan 27;95:54–72.

45. Mawjee K, Woltering S, Lai N, Gotlieb H, Kronitz R, Tannock R. Working Memory Training in ADHD: Controlling for Engagement, Motivation, and Expectancy of Improvement (Pilot Study). J Atten Disord. 2017 Sep;21(11):956–68.

46. Mawjee K, Woltering S, Tannock R. Working Memory Training in Post-Secondary Students with ADHD: A Randomized Controlled Study. PLoS One [Internet]. 2015 Sep 23 [cited 2019 Jun 18];10(9). Available from: https://www.ncbi.nlm.nih.gov/pmc/articles/PMC4580470/

47. Salmi J, Soveri A, Salmela V, Alho K, Leppämäki S, Tani P, et al. Working memory training restores aberrant brain activity in adult attention‐deficit hyperactivity disorder. Hum Brain Mapp. 2020 Dec;41(17):4876–91.

48. Stern A, Malik E, Pollak Y, Bonne O, Maeir A. The Efficacy of Computerized Cognitive Training in Adults With ADHD: A Randomized Controlled Trial. J Atten Disord. 2016 Dec;20(12):991–1003.

49. Woltering S, Gu C, Liu ZX, Tannock R. Visuospatial Working Memory Capacity in the Brain After Working Memory Training in College Students With ADHD: A Randomized Controlled Trial. Journal of attention disorders. 2019;108705471987948.

50. Conners CK. Conners’ Rating Scales--revised: User’s Manual. Multi-Health Systems, Incorporated; 1997.

51. Gioia G, Isquith P, Guy S, Kenworthy L. Behavior rating inventory of executive function. Child Neuropsychology. 2010 Aug 9;September 2000:235–8.

52. Barkley RA, Edwards GH, Robin AL. Defiant Teens: A Clinician’s Manual for Assessment and Family Intervention. New York. Guilford Publications, Inc; 1999.

53. DuPaul GJ, Power TJ, Anastopoulos AD, Reid R. ADHD Rating Scale—IV: Checklists, norms, and clinical interpretation. New York, NY, US: Guilford Press; 1998. viii, 79 p. (ADHD Rating Scale—IV: Checklists, norms, and clinical interpretation).

54. Zhang S, Faries D e., Vowles M, Michelson D. ADHD rating scale IV: psychometric properties from a multinational study as clinician-administered instrument. International Journal of Methods in Psychiatric Research. 2005;14(4):186–201.

55. Pelham WE, Gnagy EM, Greenslade KE, Milich R. Teacher ratings of DSM-III—R symptoms for the disruptive behavior disorders. Journal of the American Academy of Child & Adolescent Psychiatry. 1992;31(2):210–8.

56. Swanson JM, Kraemer HC, Hinshaw SP, Arnold LE, Conners CK, Abikoff HB, et al. Clinical relevance of the primary findings of the MTA: success rates based on severity of ADHD and ODD symptoms at the end of treatment. J Am Acad Child Adolesc Psychiatry. 2001 Feb;40(2):168–79.

57. Conners CK. Conners 3-Parent Short Form. North Tonawanda, NY: Multi-Health Systems Inc[Google Scholar]. 2008;

58. Conners CK, Erhardt D, Sparrow E. Conner’s adult ADHD rating scales: Technical manual. Multi-Health Systems Incorporated (MHS); 1999.

59. Swanson JM, Schuck S, Porter MM, Carlson C, Hartman CA, Sergeant JA, et al. Categorical and Dimensional Definitions and Evaluations of Symptoms of ADHD: History of the SNAP and the SWAN Rating Scales. Int J Educ Psychol Assess. 2012 Apr;10(1):51–70.

60. Thorell LB, Chistiansen H, Hammar M, Berggren S, Zander E, Bölte S. Standardization and cross-cultural comparisons of the Swedish Conners 3® rating scales. Nordic Journal of Psychiatry. 2018 Nov 17;72(8):613–20.

61. Conners CK. Conners’ Rating Scales--revised: CRS-R. Multi-Health Systems North Tonawanda, NJ; 2001.

62. Conners CK, Sitarenios G, Parker JD, Epstein JN. The revised Conners’ Parent Rating Scale (CPRS-R): factor structure, reliability, and criterion validity. J Abnorm Child Psychol. 1998 Aug;26(4):257–68.

63. Farré A, Narbonne J. Assessment of Attention Deficit Hyperactivity Disorder. Madrid: TEA Editions. 2013;

64. Conners CK, Sitarenios G, Parker JD, Epstein JN. Revision and restandardization of the Conners Teacher Rating Scale (CTRS-R): factor structure, reliability, and criterion validity. J Abnorm Child Psychol. 1998 Aug;26(4):279–91.

65. Swanson JM. School-based assessments and interventions for ADD students. KC publishing; 1992.

66. Achenbach TM, Rescorla LA. Manual for the ASEBA school-age forms & profiles: child behavior checklist for ages 6-18, teacher’s report form, youth self-report: an integrated system of multi-informant assessment. University of Vermont, research center for children youth & families; 2001.

67. Gioia GA, Isquith PK, Guy SC, Kenworth L. BRIEF: Behavior rating inventory of executive function. Lutz, FL: Psychological Assessment Resources.;

68. Conners CK, Wells KC, Parker JD, Sitarenios G, Diamond JM, Powell JW. A new self-report scale for assessment of adolescent psychopathology: factor structure, reliability, validity, and diagnostic sensitivity. J Abnorm Child Psychol. 1997 Dec;25(6):487–97.

69. Shapiro ES. Academic skills problems workbook (rev.). New York: Guildford. 2004;

70. Conners CK. Conners third edition (Conners 3). Los Angeles, CA: Western Psychological Services. 2008;

71. Roth RM, Gioia GA, Isquith PK. BRIEF-A: Behavior Rating Inventory of Executive Function--adult Version. Psychological Assessment Resources; 2005.

72. Rotenberg-Shpigelman S, Rapaport R, Stern A, Hartman-Maeir A. Content validity and internal consistency reliability of the Behavior Rating Inventory of Executive Function-Adult Version (BRIEF-A) in Israeli adults with attention-deficit/hyperactivity disorder. Israeli Journal of Occupational Therapy. 2008;17(2):77–96.

73. Kessler RC, Adler L, Ames M, Demler O, Faraone S, Hiripi E, et al. The World Health Organization Adult ADHD Self-Report Scale (ASRS): a short screening scale for use in the general population. Psychol Med. 2005 Feb;35(2):245–56.

74. Smidts DP, Huizinga M. BRIEF executieve functies gedragsvragenlijst: Handleiding. 2010;

75. Gray S, Woltering S, Mawjee K, Tannock R. The Adult ADHD Self-Report Scale (ASRS): utility in college students with attention-deficit/hyperactivity disorder. PeerJ. 2014;2:e324.
